# Design principles for photoswitchable fluorescent dyads

**DOI:** 10.1039/d5sc06258f

**Published:** 2025-10-16

**Authors:** Sili Qiu, Andrew T. Frawley, Kathryn G. Leslie, Xingyu Qiu, Harry L. Anderson

**Affiliations:** a Chemistry Research Laboratory, Department of Chemistry, University of Oxford Oxford OX1 3TA UK andrew.frawley@chem.ox.ac.uk harry.anderson@chem.ox.ac.uk

## Abstract

Photoswitchable fluorophores offer precise optical control for advanced imaging, yet the design criteria for an efficient photoswitchable fluorescent dye remain poorly understood. Here, we synthesize four new FRET-based dyads combining different photoswitches with fluorescent dyes and compare them with three previously reported dyads. Dithienylethene-based systems exhibit fluorescence modulation, but diazocine- and fulgimide-based dyads show minimal quenching, despite large FRET overlap. To explain these discrepancies, we develop a model showing that fluorophore absorption at the photoswitching wavelength can induce dye-mediated back-isomerization, reducing the population of the quenching-state. This model allows accurate prediction of photostationary state distributions across all molecular photoswitches and photoswitchable fluorescent dyads and allows us to identify key design principles for high-contrast photoswitchable fluorescent dyes.

## Abstract 2

Photoswitchable fluorophores offer precise optical control for advanced imaging, yet the design criteria for an efficient photoswitchable fluorescent dye remain poorly understood.

## Graphical abstract

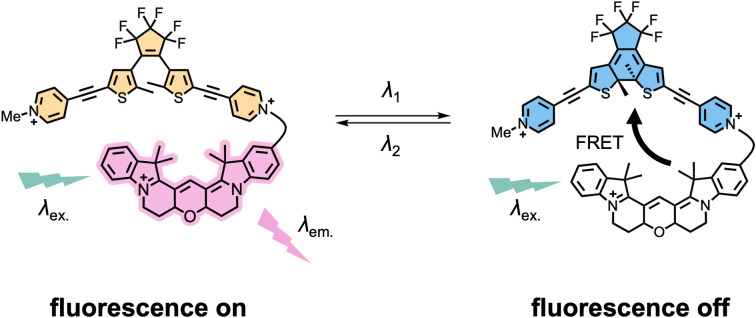

## Introduction

**Fig. 1:**
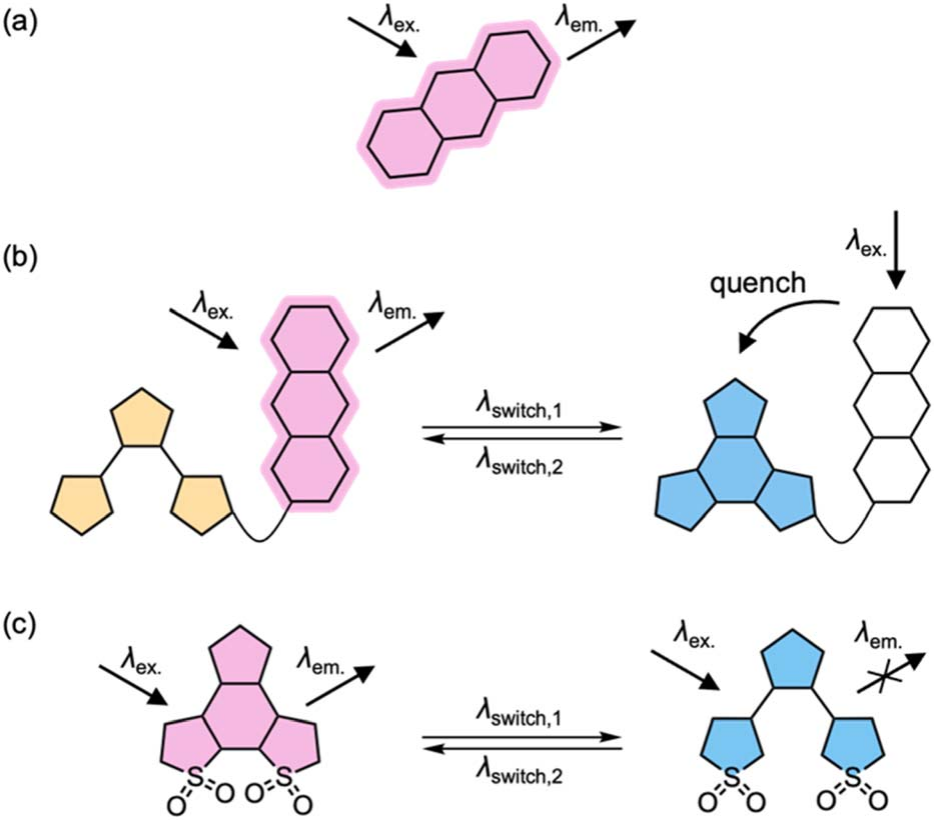
Schematic representation of (a) a conventional fluorophore without external control; (b) a dyad-based photoswitchable fluorescent dye; (c) a single-chromophore photoswitchable fluorescent dye.

Fluorescent dyes are indispensable tools^1–3^ for visualizing cellular structures^4–7^ and monitoring biomolecular interactions and dynamics^8–10^ with spatial and temporal resolution. However, conventional fluorophores lack a mechanism for external control (Fig. 1a), limiting their utility in applications requiring precise spatiotemporal modulation of fluorescence. Stimuli-responsive fluorophores address this limitation by enabling fluorescence activation in response to specific environmental cues.^11–14^ For example, pH-sensitive dyes can become highly emissive under acidic conditions.^1,15,16^ Some dyes emit only upon binding to molecular targets,^17–19^ including recently developed rhodamine derivatives optimized for live-cell labeling *via* a reversible equilibrium between a nonfluorescent spirocyclic state and a fluorescent zwitterionic state.^20^

Photoswitchable fluorophores enable reversible control of fluorescence with light as the external trigger (Fig. 1b and c).^21–34^ These systems switch between an emissive (on, non-quenching, nq) and a non-emissive (off, quenching, q) state upon irradiation at specific wavelengths. They are important in super-resolution microscopy techniques such as reversible saturable optical fluorescence transitions (RESOLFT)^25,35–41^ and single-molecule localization (SLM) microscopy,^42^ where repeated on–off switching of fluorophores is exploited to overcome the diffraction limit.^43^ In single-molecule tracking, photoswitchable dyes also permit dense labeling while selectively activating sparse subsets to prevent signal overlap.^44^

Photoswitching systems rely on molecules that can reversibly interconvert between (meta)stable isomeric forms upon light irradiation.^45^ In systems where fluorescence is externally regulated by such photoswitches, distinct wavelengths are used for photoswitching and fluorescence excitation, enabling independent control over the two molecular components.^34–37,46,47^ This is typically achieved through dyad architectures in which a non-emissive photoswitch quenches or restores the fluorescence of a covalently-linked fluorophore *via* mechanisms such as Förster resonance energy transfer (FRET) or photoinduced electron transfer (PET).^48^ Alternatively, some systems employ a single chromophore that is intrinsically fluorescent in one isomeric state but non-fluorescent in the other (Fig. 1c).^24,25,41,49–51^ In these systems, the emissive isomer's excited state can decay either through fluorescence emission or *via* competing photoisomerization.^51,52^

One major advantage of the dyad approach (Fig. 1b) is that it offers a modular strategy, enabling independent optimization of the photoswitch and the fluorescent dye. It requires the photoswitch to have distinct absorption profiles in its two states, and the fluorophore's emission must be efficiently quenched in the off state while remaining unquenched in the on state. Achieving effective FRET-based quenching depends on spectral overlap between the emission of the fluorescent dye and the absorption of the photoswitch in its quenching state.

Here, we report the design, synthesis, and characterization of four photoswitchable dyads employing FRET-based quenching (Fig. 3 later). Using a modular strategy, we combined different photoswitches with selected fluorescent dyes and systematically evaluated their photochemical and photophysical properties. Only the dithienylethene (DTE) systems paired with Cy3 and Cy3B dyes exhibited efficient on-off switching, while the diazocine (DAZ)-rhodamine B (RhoB) and fulgimide (FULG)-Atto-590 (RhoX) systems showed minimal quenching in the off state, despite significant spectral overlap between the emission of the fluorescent dye and the absorption of the quenching-state photoswitch—conditions that should support efficient FRET. To account for these discrepancies, we investigated the underlying photophysical and photochemical behavior of each dyad and developed a model that links photoswitch and fluorophore absorption at the switching wavelength, and the ratio of forward to reverse photoswitching quantum yields to the resulting photostationary state distribution (PSD) of the photoswitch on its own or in a dyad. To validate this model, we further applied it to three previously studied spironaphthoxazine (SO)-based dyads with high switching contrast (Fig. 3 later).^35–37^ The model accurately accounted for their outstanding performance. Guided by this insight, we propose general design principles for engineering dyad-based photoswitchable fluorescent dyes that achieve high-efficiency fluorescence modulation.

## Results and discussion

### Selection of the photoswitch

Designing an effective photoswitchable fluorescent dye requires careful selection of the photoswitch component to ensure reliable modulation of fluorescence. An ideal photoswitch must possess several key properties. First, good absorption spectral separation between its two isomeric states is essential: the quenching state must absorb strongly in the region overlapping the emission of the fluorescent dye, while the non-quenching state should not. Such absorption band separation will also favor a high PSD in both directions under irradiation, ensuring efficient and complete conversion between states for strong on–off contrast. Second, high photoswitching quantum yields in the forward direction enable rapid switching with low light doses while moderate reverse photoswitching quantum yield avoids photoreversion after a single FRET event. Third, fatigue resistance is critical to maintain performance over many switching cycles without degradation. Fourth, visible-light addressability (>400 nm) for both directions of photoswitching is preferred to minimize photodamage and maximize compatibility with biological imaging microscopes. Finally, the switch should have large oscillator strengths leading to intense absorption bands (high molar absorption coefficients) for efficient excitation and efficient FRET.

**Fig. 2:**
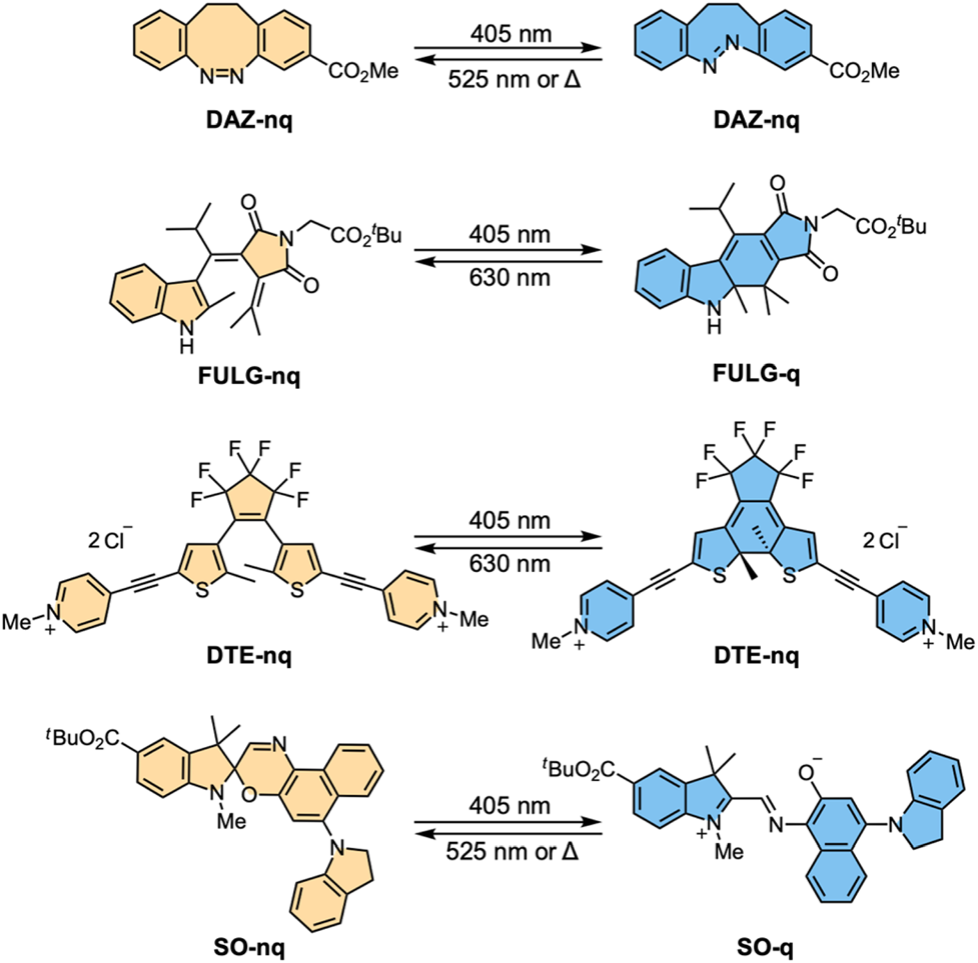
Photoswitching of DAZ, FULG, DTE and previously reported SO;^35^ nq denotes the non-quenching state, and q denotes the quenching state.

Based on these criteria, three photoswitches were selected for this study (Fig. 2): a diazocine (DAZ), a fulgimide (FULG), and a dithienylethene (DTE). Diazocines are bridged azobenzene derivatives that undergo reversible *Z*–*E* isomerization upon irradiation.^53^ Fulgimides and dithienylethenes switch between open and closed forms *via* a 6π-electrocyclization mechanism.^54^ Their closed isomers exhibit strong visible absorption, ensuring good spectral separation and bistability.^55^ For each class, we synthesized a capped analogue (Fig. 2) as a reference compound for analyzing the photoswitching properties. The diazocine and fulgimide photoswitches were prepared as alkyl esters at their carboxylic acid positions. For dithienylethene, the pyridine end groups were methylated to form *N*-methylpyridinium units, mimicking the dicationic character of the targeted DTE structures.^56^ We compared a previously reported spironaphthoxazine (SO) photoswitch capped with an alkyl ester which demonstrated outstanding photoswitching behavior (Fig. 2).^35–37^

The photophysical and photochemical properties of each photoswitch were evaluated for their suitability for incorporation into photoswitch-fluorophore dyads. Specifically, we measured the absorption spectra of both isomeric states (Section S3, and Fig. S4–S7), photoswitching quantum yields (*Φ*_s,1_ and *Φ*_s,−1_, where *Φ*_s,1_ represents the forward switching quantum yield from the non-quenching state to the quenching state and *Φ*_s,−1_ represents the reverse switching quantum yield, Section S5), and PSDs (Section S6) under controlled irradiation conditions (Table 1). Forward switching—from the non-quenching to the quenching state—was induced using 405 nm light, while reverse switching was triggered by 525 nm light for DAZ and SO, and by 630 nm light for FULG and DTE. Owing to differing solubility, photoswitching behavior of DAZ, FULG, DTE and SO was assessed in DMSO, MeCN, water and cyclohexane, respectively. DAZ exhibited distinct absorption maxima at 404 nm and 493 nm for the non-quenching and quenching isomers, respectively, and reached a forward PSD of 72% under 405 nm irradiation. FULG showed a greater absorption spectral separation of 167 nm and achieved a higher forward PSD of 87%. DTE displayed the largest absorption band separation of 273 nm and a forward PSD of 96%. SO demonstrated an absorption shift of 183 nm with a forward PSD of 85%. For all four photoswitches, the non-quenching state could be fully regenerated, achieving 100% reverse PSD. All compounds showed robust fatigue resistance under repeated switching, consistent with previous reports.^35,54,56,57^

**Table 1:** Key photoswitching parameters of the selected capped photoswitches^e^

Photoswitch	*λ*_max,nq_ (nm)	*λ*_max,q_ (nm)	*Φ*_s,1_ (%)	*Φ*_s,−1_ (%)	PSD_nq–q_ (%)
DAZ^a^	404	493	15	35	72
FULG^b^	356	523	21	3.0	87
DTE^c^	377	650	17	0.12	96
SO^d^	388	561	8.0	1.1	85

a Measured in DMSO.

b Measured in MeCN.

c Measured in water.

d Measured in cyclohexane.

e For DAZ and SO, the forward and reverse photoswitching are triggered by 405 nm and 525 nm respectively. For FULG and DTE, forward and reverse photoswitching are triggered by 405 nm and 630 nm respectively. SO is reported in our previous work.^35^

### Design and synthesis of photoswitch-fluorophore dyads

Having established suitable photoswitches, we next focused on designing and synthesizing photoswitch-fluorophore dyads capable of efficient fluorescence modulation. To identify suitable fluorophores, we surveyed previously studied dyes to find those with emission spectra that overlap significantly with the absorption spectra of the photoswitches in their quenching states, enabling effective FRET quenching (Section S8).

**Fig. 3:**
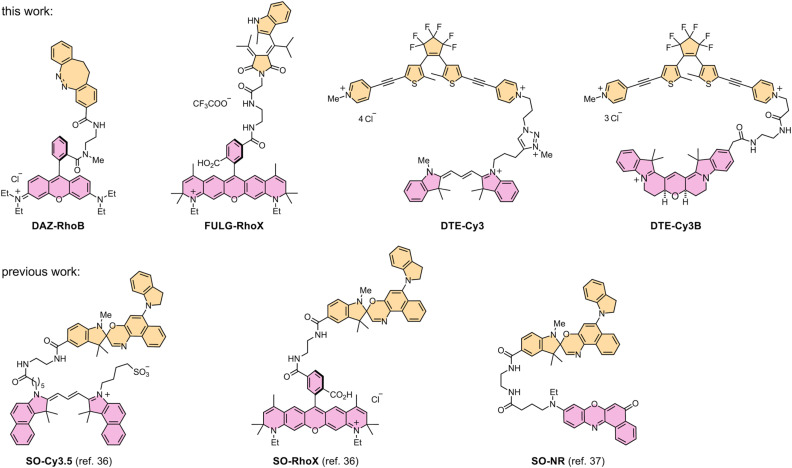
Structures of the seven photoswitchable fluorescent dyads compared in this study, synthesized during this work (top) and previously (bottom).^36,37^

To quantitatively assess this compatibility, we calculated the FRET efficiency of each photoswitch-fluorophore pair using the equations described in Section S8. Specifically, we measured the absorption spectra of the photoswitches in their quenching states and the fluorescence emission spectra of the selected dyes, from which spectral overlap integrals were determined for the DAZ-RhoB, FULG-RhoX, DTE-Cy3, and DTE-Cy3B dyads (Fig. 3, Table S4 and Section S8). We computed the corresponding Förster distances for each pair using the spectral overlap integral values. To estimate donor–acceptor (D–A) separation, we constructed molecular models of each dyad in their fully extended conformations using the MMFF94 force field (Tables 2, S5 and Section S8). These modeled distances represent upper bounds, and the flexible linkers are expected to adopt shorter separations in solution. Crucially, even at these maximal distances, all dyads were predicted to exhibit excellent FRET efficiencies (>94%), suggesting that they would support effective fluorescence quenching. Similarly, previously studied SO-Cy3.5, SO-RhoX and SO-NR dyads all exhibited outstanding calculated FRET efficiency (>99%; Table 2).^36,37^

**Table 2:** Calculated FRET efficiencies of dyads

Dyad	Max D–A distance (Å)	FRET efficiency
DAZ-RhoB^a^	10.3	94.3%
FULG-RhoX^b^	17.6	98.5%
DTE-Cy3^c^	24.3	96.1%
DTE-Cy3B^c^	28.2	97.8%
SO-Cy3.5^d^	18.6	99.3%
SO-RhoX^d^	15.7	99.9%
SO-NR^d^	47.2	99.1%

a FRET efficiency calculations were performed based on photophysical data in DMSO.

b FRET efficiency calculations were performed based on photophysical data in MeCN.

c FRET efficiency calculations were performed based on photophysical data in water.

d FRET efficiency calculations were performed based on photophysical data in cyclohexane.

Following these design considerations, we synthesized all four dyads (Fig. 3). For each system, the photoswitch and the fluorophore were prepared separately and then coupled together (Sections S2 and S12). To synthesize DAZ-RhoB, DAZ and RhoB were deprotected and then coupled *via* HBTU-mediated amide bond formation (Scheme S1). Similarly, FULG-RhoX was synthesized using amide coupling (Scheme S3). For DTE-Cy3, a Cy3 fluorophore with an alkyne handle was first prepared (Scheme S4). The alkyne was then linked to an alkyl iodide using copper-catalyzed azide–alkyne cycloaddition (CuAAc). Cy3 dye and DTE photoswitch were coupled together *via* nucleophilic substitution (Scheme S5). DTE-Cy3B was assembled using amide coupling with an ethylene diamine linker similar to DAZ-RhoB. In the final step, the remaining free pyridine group of the DTE core was methylated to introduce the *N*-methyl pyridinium functionality (Scheme S7).^56^ Previously studied SO-Cy3.5, SO-RhoX and SO-NR were all synthesized using amide coupling.^36,37^ Full details of the syntheses are reported in the SI (Sections S2 and S12).

### Fluorescence quenching

**Fig. 4:**
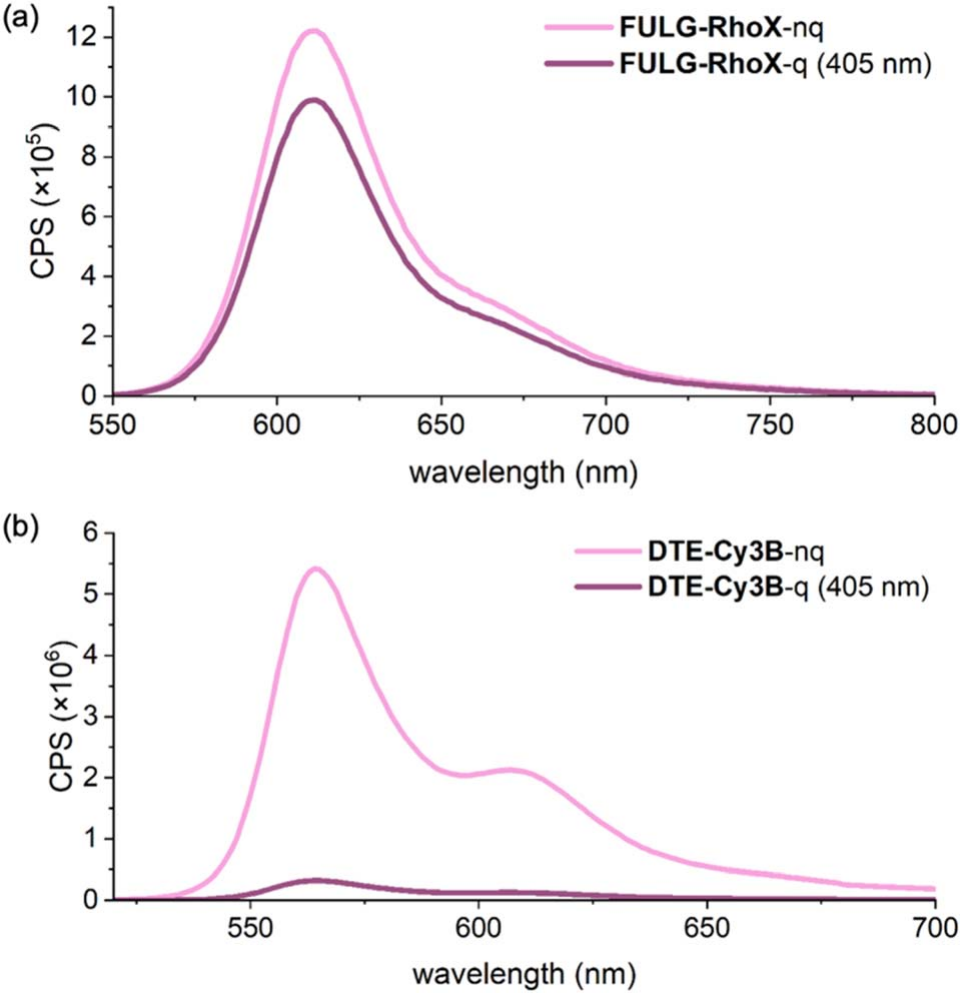
Fluorescence spectra of (a) FULG-RhoX (measured in MeCN) and (b) DTE-Cy3B (measured in water) in the emissive and the non-emissive states at 25 °C.

A critical parameter for assessing the effectiveness of each photoswitch-fluorophore dyad is the extent of fluorescence quenching between its emissive (non-quenching) and non-emissive (quenching) states. 100% quenching would indicate complete suppression of fluorescence in the non-emissive state, delivering highly efficient light-controlled modulation. To evaluate this parameter, we first measured the fluorescence intensity of the dyads with the photoswitch component in its non-quenching state (Fig. 4 and Section S10). Samples were then irradiated with 405 nm light to induce isomerization of the photoswitch into its quenching state (irradiated until no further change was observed), after which the fluorescence signal was measured again. The integrated fluorescence intensities of the bright and dark states were compared to calculate the percentage quenching for each dyad. These quenching values are reported in Table 3 as the experimental PSD (of the photoswitch). We assume that the observed fluorescence quenching directly corresponds to the PSD of the photoswitch, based on the near-quantitative FRET efficiency. This assumption is supported by our FRET modeling (Table 2), which predicts >94% FRET efficiency even at the maximal estimated donor–acceptor distance; in solution, the flexible linker probably adopts shorter average distances, further enhancing energy transfer. To validate this assumption, we independently measured the PSDs of the DTE component in DTE-Cy3 and DTE-Cy3B dyads *via*^1^H NMR spectroscopy (Section S11). The PSD values derived from fluorescence quenching and NMR were in close agreement, with discrepancies of less than 2%.

**Table 3:** Experimental and calculated PSD of photoswitches and photoswitchable fluorescent dyads under irradiation at 405 nm. Calculated PSDs were estimated using eqn (2)–(4). The molar absorption coefficients for such calculation are shown in the table. The photoswitching quantum yields and FRET efficiencies are given in Tables 1 and 2, respectively. *K* (either *K*_switch_ or *K*_dyad_ in eqn (3) or (4), depending on the system) is the photodynamic equilibrium constant

Compound		*ε*_s-nq-405_ (M^−1^ cm^−1^)	*ε*_s-q-405_ (M^−1^ cm^−1^)	*ε*_f-405_ (M^−1^ cm^−1^)	*K*	Exp. PSD (%)	Calc. PSD (%)
Photoswitches	DAZ^a^	750	170	n.a.	1.93	72^f^	66
	FULG^b^	840	610	n.a.	7.24	87^f^	87
	DTE^c^	9800	12 000	n.a.	118	96^f^	99
	SO^d^	15 000	11 000	n.a.	9.91	85–95^g^	91
Dyads	DAZ-RhoB^a^	750	170	2200	0.14	7^h^	13
	FULG-RhoX^b^	840	610	9800	0.43	19^h^	30
	DTE-Cy3^c^	9800	12 000	470	110	94^h^ (94)^f^	99
	DTE-Cy3B^c^	9800	12 000	430	110	93^h^ (92)^f^	99
	SO-Cy3.5^e^	15 000	11 000	910	9.2	85^i^	90
	SO-RhoX^e^	15 000	11 000	9800	5.5	80^i^	85
	SO-NR^e^	15 000	11 000	180	9.8	90^i^	91

a Measured in DMSO.

b Measured in MeCN.

c Measured in water.

d Measured in cyclohexane.

e Measured under microscopy conditions as illustrated by the original paper.^36,37^

f Determined using ^1^H NMR spectroscopy.

g Determined using UV-vis absorption spectroscopy.

h Determined by fluorescence quenching measured in a fluorimeter.

i Determined by fluorescence quenching under microscopy conditions. All measurements were performed at 25 °C.

**Fig. 5:**
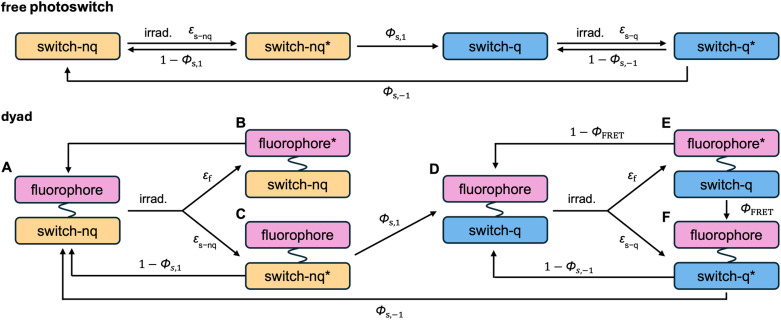
Schematic photophysical model showing the possible states that can be accessed by a photoswitch (top) and a photoswitch-fluorophore dyad (bottom). (A–F) denote different energetic states.

**Fig. 6:**
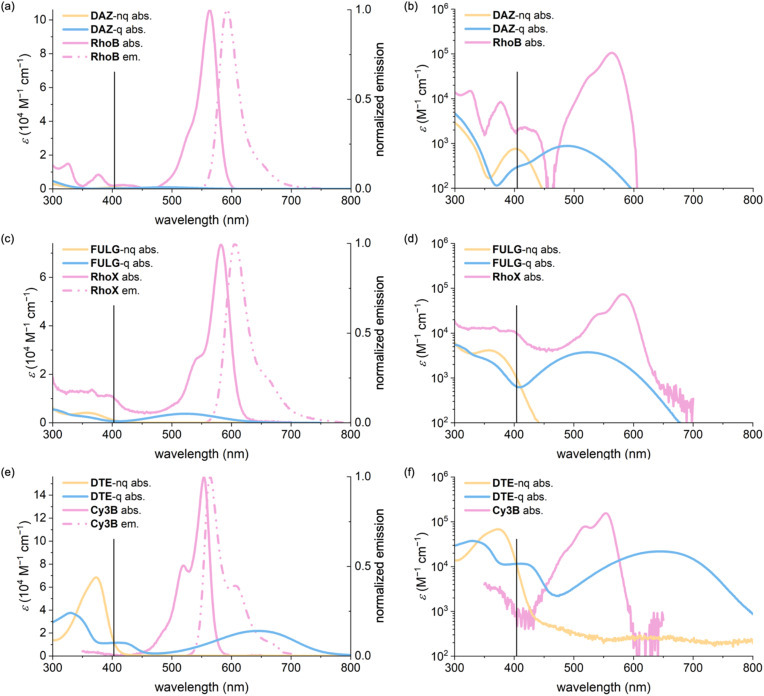
Absorption spectra of the fluorophore (solid pink ), the photoswitch in its non-quenching form (orange), and quenching form (blue), alongside the normalized emission spectrum of the fluorophore (dotted pink ) for (a and b) DAZ/RhoB, (c and d) FULG/RhoX and (e and f) DTE/Cy3B. Spectra are shown on a linear scale (left column) and logarithmic scale (right column). The black vertical line marks the wavelength used for forward photoswitching (405 nm). In DAZ-RhoB and FULG-RhoX, the fluorophore exhibits significantly stronger absorption than the photoswitch at 405 nm, triggering dye-mediated back-isomerization. In contrast, for DTE-Cy3B, the photoswitch absorbs more strongly than the fluorophore at this wavelength, favoring efficient switching by minimizing competing excitation pathways.

The fluorescent quenching results (experimental PSD values in Table 3) reveal excellent performance for the DTE-based systems, which achieve over 90% quenching, whereas DAZ-RhoB and FULG-RhoX show only minimal quenching of 7% and 19%, respectively. To understand the poor fluorescence quenching observed in DAZ-RhoB and FULG-RhoX, we considered several possible explanations. The most plausible scenario is that during 405 nm irradiation, the fluorophore itself absorbs light and becomes excited while the photoswitch is isomerized to its quenching form. This excited-state fluorophore can efficiently transfer energy to the photoswitch *via* FRET, driving photoisomerization back to the non-quenching form (Fig. 5 bottom). As a result, the PSD becomes significantly shifted toward the emissive species, reducing the population of the quenching state and diminishing the observed fluorescence contrast. Dye-mediated back-isomerization is favored by the strong 405 nm absorption of the rhodamine-based dyes used in DAZ-RhoB and FULG-RhoX, in contrast to the minimal absorption at 405 nm of the cyanine dyes in the DTE-based systems (Fig. 6 and Table 3).

We also considered and ruled out other potential explanations. Thermal back-relaxation, in which the photoswitch reverts from the quenching to the non-quenching state spontaneously, is unlikely: DAZ exhibits a thermal half-life of about 11 hours (Fig. S10),^57^ and fulgimides are known to be thermally irreversible.^54^ Another possibility is inefficient FRET despite successful isomerization to the quenching state; however, our modeling indicates >94% FRET efficiency for all dyads even at maximal linker extension. Finally, we ruled out the possibility that 405 nm irradiation fails to initiate photoswitching altogether, as all free photoswitches demonstrated excellent switching performance under identical conditions.

For previously reported dyads incorporating the thermally reversible SO photoswitch,^36,37^ including SO-Cy3.5, SO-RhoX, and SO-NR, fluorescence quenching could not be quantified using the steady-state bulk method, since the photoswitch spontaneously reverts on the time scale of measurement. Instead, fluorescence quenching efficiency was assessed under microscopy conditions, where individual pixels were monitored for changes in fluorescence intensity before and after photoactivation. This method enables the measurement of transient quenching despite thermal reversibility. All SO-based dyads demonstrate outstanding fluorescence quenching efficiencies (expressed as experimental PSD in Table 3) and enabled resolution enhancement in RESOLFT microscopy.

### Dyad photodynamic model

A central challenge in designing effective photoswitchable fluorescent dyads lies in ensuring that the photoswitch component can reliably modulate the fluorescence emission of the attached dye. The key parameter governing this modulation is the PSD under continuous irradiation, which defines the fraction of molecules residing in the quenching state. Upon irradiation, the system reaches a dynamic equilibrium between the non-quenching and quenching isomers of the photoswitch. This equilibrium is characterized by photodynamic equilibrium constant *K*, defined by the ratio of the forward and reverse photoswitching rates, or the ratio of the concentration of the two isomers (eqn (1)). The PSD is then derived from photodynamic equilibrium constant *K* according to eqn (2).1
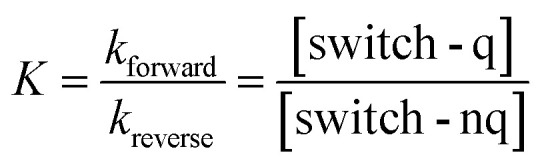
2
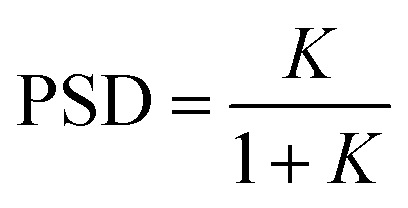
In a free photoswitch (Fig. 5, top), irradiation at a wavelength selectively absorbed by the non-quenching state (orange) promotes its excitation, which can lead to productive photoisomerization into the quenching state (blue) with a quantum yield *Φ*_s,1_. Alternatively, the excited non-quenching state may undergo non-productive decay back to the ground state with a probability of 1 − *Φ*_s,1_. The quenching state can similarly undergo reverse photoisomerization under suitable irradiation, with a quantum yield of *Φ*_s,−1_. In this simple two-state system, the equilibrium constant *K*_switch_ depends solely on the molar absorption coefficients and quantum yields of the two isomeric states, as described in eqn (3).3
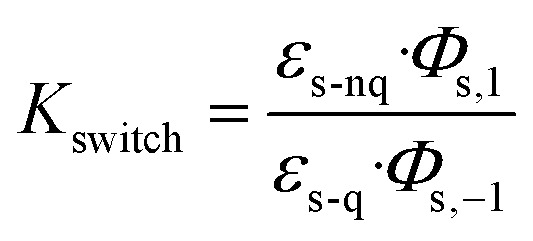


However, in a dyad construct (Fig. 5, bottom), where the photoswitch is covalently linked to a fluorophore, the photodynamics become more complex. Both components (photoswitch and fluorescent dye) can absorb light at the irradiation wavelength, and their relative excitation probabilities are determined by the ratio of their molar absorption coefficients. When the dyad is in ground state A (non-quenching), irradiation can excite either the fluorophore, leading to excited state B, or the photoswitch, yielding excited state C. Excited state B relaxes back to ground state A *via* radiative and non-radiative decay, while excited state C may undergo photoisomerization to generate the quenching ground state D (with a quantum yield of *Φ*_s,1_) or decay non-productively back to state A (with a quantum yield of 1 − *Φ*_s,1_)—mimicking the behavior of a free photoswitch. Upon light absorption by the quenching state D, both components may again be excited. Fluorophore excitation leads to excited state E, which can relax through either direct fluorescence or energy transfer *via* FRET to the photoswitch. Given the high FRET efficiencies calculated for our dyads, this process predominantly results in the formation of excited state F, where the photoswitch is now in its excited quenching form. From this state, the system can either undergo reverse photoisomerization to return to ground state A or relax back to D without switching. The probability of this reverse isomerization is governed by *Φ*_s,−1_, the reverse photoisomerization quantum yield.

This FRET-mediated back-isomerization is a photophysical pathway that does not exist in the free photoswitch. As a result, the effective rate of the q-to-nq transition is increased in the dyad under irradiation conditions that excite both the fluorophore and the photoswitch. Consequently, the photodynamic equilibrium constant *K*_dyad_ that defines the PSD in the dyad must be modified to account for this additional channel. Eqn (4) introduces an extra term in the denominator to represent the contribution of dye excitation followed by FRET-induced reverse isomerization. This additional decay pathway reduces the effective population of the quenching state under irradiation, thereby lowering *K*_dyad_, diminishing the PSD, and ultimately decreasing the fluorescence modulation contrast.4
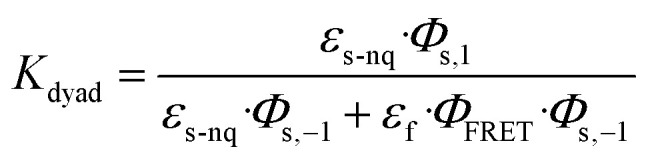


To evaluate the predictive power of our model, we first calculated the PSDs of the free photoswitches using their molar absorption coefficients at 405 nm (for both the non-quenching and quenching states, Table 3) and their respective forward and reverse photoswitching quantum yields (Table 1, and eqn (3)). These calculated values were then compared with experimental PSDs measured independently by ^1^H NMR spectroscopy (Table 3, and Section S6).^56^ For the free photoswitches, the calculated PSDs were 66% for DAZ, 87% for FULG, 99% for DTE, and 91% for SO closely matching the experimentally determined values of 72%, 87%, 96%, and 85–95% respectively (Table 3). The PSD range reported for SO reflects its fast thermal back-isomerization, which precludes steady-state ^1^H NMR spectroscopy analysis; instead, PSD was estimated using transient UV-vis and IR spectroscopies on a picosecond to microsecond timescale.^35^ Despite this limitation, the strong agreement across all systems validates the two-state photophysical model in the absence of a fluorophore.

We next applied the extended dyad model (eqn (4)), which further accounts for the absorption of the fluorophore and the calculated FRET efficiency to predict the PSDs within the dyads. The resulting calculated PSDs were 13% for DAZ-RhoB, 30% for FULG-RhoX, and 99% for both DTE-Cy3 and DTE-Cy3B (Table 3 right-hand column). These predictions correlate well with the experimentally observed PSDs—7%, 19%, 94%, and 93%, respectively (Table 3)—determined by fluorescence quenching. This agreement supports the conclusion that fluorophore absorption and dye-mediated back-isomerization can significantly perturb the PSD of the photoswitch component in dyads, underscoring the importance of considering such effects during molecular design.

To further evaluate the generalizability of our model, we applied it to SO-Cy3.5, SO-RhoX, and SO-NR that have been successfully used in RESOLFT imaging.^36,37^ Due to the rapid thermal reversion of the spironaphthoxazine photoswitch, PSD for these dyads could not be directly determined by ^1^H NMR spectroscopy or standard fluorometric measurements. Instead, their fluorescence quenching efficiencies were assessed under microscopy conditions, where changes in signal intensity within individual pixels were monitored following photoswitching. The observed quenching efficiencies (Table 3) at optimal imaging conditions—85% for SO-Cy3.5, 80% for SO-RhoX, and 90% for SO-NR—closely matched the PSDs predicted by our model (90%, 85%, and 91%, respectively), providing further validation of the model's predictive power even in systems where rapid thermal dynamics complicate conventional characterization. Notably, the model does not account for thermal relaxation of the quenching state of SO, which may contribute to the discrepancy. Nonetheless, the strong agreement underscores the robustness and applicability of our model to dynamic systems used in practical imaging applications.

### Dyad design principles

In our previous work,^35^ we outlined six key design principles for photoswitchable fluorescent dyads in the context of super-resolution microscopy:

(1) A stable emissive state excited by visible light;

(2) High fluorescence contrast governed by photoswitch PSD;

(3) Strong fatigue resistance under biological conditions;

(4) High brightness and low cytotoxicity;

(5) Visible-light addressability (*λ* > 400 nm);

(6) Cell permeability for intracellular labeling.

While these criteria remain foundational, they overlook the photodynamic behavior that arises in covalently linked dyads. In such systems, the achievable fluorescence contrast is governed not only by the spectral overlap necessary for FRET quenching but also by additional light-driven pathways that can perturb the PSD of the photoswitch and thus the effectiveness of fluorescence modulation. Here we outline additional principles that are key to ensure high PSD in a dyad.

A critical parameter in dyad design is the relative absorption of the fluorophore and photoswitch at the photoswitching wavelength (from non-quenching to quenching state), typically at 405 nm. Fluorophores such as rhodamines often have high molar absorption coefficients at this wavelength—for example, RhoB and RhoX show *ε* values of 2200 and 9800 M^−1^ cm^−1^ at 405 nm, respectively (Fig. 6). In dyads such as DAZ-RhoB and FULG-RhoX, this results in the fluorophore outcompeting the photoswitch for excitation. Once excited, the fluorophore can undergo FRET to the photoswitch in its quenching state, inducing undesired back-isomerization to the non-quenching form. This dye-mediated reversal of the photoswitching process shifts the PSD toward the emissive species, ultimately limiting the fluorescence quenching efficiency to 7–19%.

In contrast, fluorophores with minimal absorption at the switching wavelength, such as cyanines, are far less prone to this issue. For instance, Cy3B exhibits negligible absorption at 405 nm (*ε* = 430 M^−1^ cm^−1^), whereas the corresponding DTE photoswitch absorbs strongly at the same wavelength (*ε* = 9800 M^−1^ cm^−1^) (Fig. 6). This enables efficient photoswitch excitation and suppresses dye-mediated interference, resulting in a PSD of 92% (measured by ^1^H NMR spectroscopy, Section S11) and 93% fluorescence quenching in the DTE-Cy3B dyad (Table 3, and Section S10).

In systems where both the fluorophore and the photoswitch absorb strongly at the switching wavelength, as in SO-RhoX, the relative quantum yields of forward and reverse switching become decisive. Photoswitch SO absorbs strongly at 405 nm (*ε* = 15 000 M^−1^ cm^−1^), higher than RhoX (9800 M^−1^ cm^−1^), allowing it to dominate excitation. Nevertheless, the absorbance of RhoX is not negligible. It is the high forward switching quantum yield (*Φ*_s,1_ = 0.080) and low reverse switching quantum yield (*Φ*_s,−1_ = 0.011) of SO that ensure efficient accumulation in the quenching state and minimize FRET-induced back-isomerization. This balance enables SO-RhoX to maintain high quenching efficiency (∼85%).^36^ FRET-based photoswitchable dyads inevitably suffer to some extent from destructive readout; *i.e.*, excitation of their fluorophore unit for fluorescence detection leads to unwanted back-photoisomerization *via* FRET to the photoswitch. This unwanted photo-reversion is minimized by a low value of *Φ*_s,−1_. On the other hand, if *Φ*_s,−1_ is too low, the switching cycle becomes very slow, so a compromise is required.

These results underscore that the design of high-performance photoswitchable fluorophore dyads requires more than ensuring sufficient spectral overlap for FRET quenching. Instead, the interplay between molar absorption coefficient at the switching wavelength and the quantum yields of photoisomerization in both directions must be optimized. Ideal systems feature a photoswitch that dominates excitation at the switching wavelength, a fluorophore with minimal competing absorption, and a high ratio of forward to reverse quantum yield. Together, these parameters govern the PSD in the dyad and ultimately determine the dynamic range of fluorescence modulation achievable for imaging and photonic applications.

## Conclusions

In this study, we designed, synthesized, and systematically tested a series of photoswitch-fluorophore dyads based on FRET quenching to achieve reversible fluorescence modulation. Among the four new dyads, only the DTE-based systems with Cy3 and Cy3B fluorophores demonstrated high switching contrast, while the DAZ-RhoB and FULG-RhoX systems showed limited fluorescence quenching, despite favorable spectral overlap, high FRET efficiencies and excellent performance in the isolated switches. To understand this discrepancy, we developed a photodynamic model that accounts for not only the intrinsic photoisomerization parameters of the photoswitch but also the impact of fluorescent dye excitation and energy transfer within the dyad. This model reveals that fluorophore absorption at the photoswitching wavelength can significantly perturb the PSD, primarily through dye-mediated back-isomerization of the photoswitch. Incorporating this effect into our photodynamic equilibrium model allowed us to accurately predict the PSD and correlate it with observed fluorescence quenching. The poor performance of the DAZ-RhoB dyad can be attributed both to the significant absorption of RhoB at 405 nm and to the low oscillator strengths of DAZ, which lead to weak absorption by the non-quenching form at 405 nm and less efficient FRET in the quenching form of the switch.

Our analysis illustrates the importance of considering both spectral separation and photodynamics when designing high-performance photoswitchable fluorescent dyads. Specifically, dyes with minimal absorption at the photoswitching wavelength and photoswitches with intense absorption bands, in combination with high forward and low reverse quantum yields are key to maximizing switching contrast. These insights provide a rational framework for the development of next-generation photoswitchable fluorophores with enhanced control over fluorescence output, facilitating applications in super-resolution microscopy and molecular sensing.

## Author contributions

S. Q. synthesized and characterized DTE and DTE-based dyads. A. T. F. synthesized and characterized FULG and FULG-based dyad. K. G. L. synthesized and characterized DAZ and DAZ-based dyad. X. Q. synthesized raw materials. S. Q. and A. T. F. constructed the photodynamic model. S. Q., A. T. F. and H. L. A. conceptualized the project. H. L. A. managed the project and acquired the funding. S. Q. wrote the manuscript. All authors contributed to editing and reviewing the manuscript.

## Conflicts of interest

There are no conflicts to declare.

## Supplementary Material

SC-OLF-D5SC06258F-s001

## Data Availability

The data that support the findings of this study are available in the supporting information (SI) of this article. Supplementary information: synthetic procedures and full characterization for all compounds, spectroscopic data, copies of NMR spectra, and MS data. See DOI: https://doi.org/10.1039/d5sc06258f.
