# Supplementary material for: Design principles for photoswitchable fluorescent dyads
Source: Chem Sci. 2025 Oct 16;16(46):22101–10. doi: 10.1039/d5sc06258f (PMC12542846; doi:10.1039/d5sc06258f)
Supplement: SC-016-D5SC06258F-s001 [file SC-016-D5SC06258F-s001.pdf]

Supplementary Information (SI) for Chemical Science.
This journal is © The Royal Society of Chemistry 2025

Electronic Supporting Information

Design Principles for Photoswitchable Fluorescent Dyads

Sili Qiu, Andrew T. Frawley,* Kathryn G. Leslie, Xingyu Qiu, and Harry L. Anderson*

Chemistry Research Laboratory, Department of Chemistry, University of Oxford, Oxford OX1 3TA, UK.

E-mail: andrew.frawley@chem.ox.ac.uk, harry.anderson@chem.ox.ac.uk

Contents

S1. General Procedures 2
S2. Synthesis of Compounds 5
S3. UV-Vis Spectra of the Model Photoswitches 12
S4. Determination of Molar Absorption Coefficients 15
S5. Photoswitching Quantum Yields 16
S6. Model Photoswitch PSD Determination by ^{1}H NMR 18
S7. Dye Selection 21
S8. FRET Efficiency Modelling 22
S9. Dyad UV-vis Absorption and Fluorescence 25
S10. Fluorescence Quenching 27
S11. PSD of Photoswitch in the Dyad by NMR 29
S12. Synthetic Procedures 31
References 71

S1. General Procedures

Chemical Reactions and Analysis

All reactions were performed using oven-dried reaction vessels under an argon atmosphere unless stated otherwise. Anhydrous acetonitrile (MeCN), chloroform ($CHCl_3$), dichloromethane (CH_2Cl_2), diethyl ether (Et_2O), tetrahydrofuran (THF), 1,4-dioxane, and triethylamine (Et_3N) were obtained from an MBraun MBSPS-5-BenchTop solvent purification system, having been passed through an activated alumina column under nitrogen. Methanol was dried over 4 Å molecular sieves under argon. All other chemical reagents used were commercially available from Alfa Aesar, Fluorochem, Merck, and TCI Chemicals and were used as supplied.

Thin layer chromatography (TLC) analysis was carried out using Merck aluminum-backed TLC silica gel 60 F_{254} pre-coated plates. Plates were visualized by the quenching of fluorescence under ultraviolet light (λ_{max} = 254 nm), turn-on of fluorescence under ultraviolet light (λ_{max} = 356 nm), color of fluorescence dye or color changes of photoswitches after ultraviolet light (λ_{max} = 365 nm) irradiation. Flash column chromatography and size exclusion chromatography were performed using Merck Geduran® 60 silica gel (particle size 63–200 µm) or Bio-Rad Bio-Beads S-X3 styrene divinylbenzene beads (3% cross linkage, 40–80 µm bead size, ≤2,000 MW limit), respectively, with the solvent system given. All solvents used for chromatography purification were HPLC grade or equivalent and were supplied by Merck, Fisher Scientific, VWR or Honeywell.

Reverse phase HPLC was performed at 298 K using an Agilent 1100 Series system comprising an autosampler (G1313A), a vacuum degassing unit (G1379A), a quaternary pump (G1311A), a column oven (G1316A), a diode array detector (G1315B), and a fraction collector (G1364C). The instrument was operated using ChemStation software. For analytical HPLC an Agilent Eclipse XDB-C18 column (4.6 × 150 mm, 5 µm particle size) was used with a flow rate of 1.0 mL/min.

Method A

**time (min)**	**% H_2O**	**% CH_3OH**
0	95	5
20	0	100
30	0	100
33	05	5

Method B

time (min)	% H_2O	% MeCN
0	95	5
20	0	100
30	0	100
33	05	5

Proton (^{1}H) and carbon (^{13}C) NMR spectra are recorded on either a Bruker AVIII HD 400, a Bruker AVIII HD 500, a Bruker AVII 500 with a ^{13}C(^{1}H) dual cryo-probe, or a Bruker AVIII 600 instrument with a broadband cryo-probe in deuterated solvents. ^{1}H NMR chemical shifts are reported in ppm to the nearest 0.01 ppm relative to $SiMe_4$ (δ = 0) and are referenced internally with respect to residual protons in the deuterated solvent (δ = 2.05 for acetone-d_6, 1.94 for acetonitrile-d_3, 7.26 for chloroform-d, 2.50 for DMSO-d_6, 3.31 for methanol-d_4, and 3.58 for tetrahydrofuran-d_8). ^{13}C NMR spectra are recorded with broadband decoupling. ^{13}C chemical shifts are reported in ppm to the nearest 0.1 ppm relative to $SiMe_4$ (δ = 0) and are referenced internally with respect to carbons in the solvent (δ = 118.6 for acetonitrile-d_6, 128.1 for benzene-d_6, and 77.2 for chloroform-d). Peak assignments are made based on chemical shifts, integrations, coupling constants, comparison to known compounds, and using COSY, HSQC, and HMBC experiments where appropriate. Multiplets are described as singlet (s), doublet (d), triplet (t), quartet (q), pentet (p) multiplet (m), broad (br), or combinations thereof. Coupling constants (J) are reported to the nearest 0.1 Hz.

High resolution mass spectra (HRMS) were recorded by the Chemistry Research Laboratory Mass Spectrometry Facility, University of Oxford, using a Bruker MicroTOF spectrometer or a Thermo Orbitrap Exactive mass spectrometer. The mass reported was that containing the isotopes with the lowest mass, with each value to 7 significant figures and within 5 ppm of the calculated mass. Mass to charge ratios (*m/z*) are reported in Daltons.

Optical Analysis

Stock solutions of all capped model photoswitches and dyads were prepared by dissolving in DMSO-d_6, MeCN-d_3, or D_2O containing methanol (2.34 mM) as a concentration reference. The ^{1}H NMR spectra of the samples were acquired on a 500 MHz spectrometer with an extended relaxation delay (30 s) for accurate integration. The exact concentration of the photoswitch was determined by comparing the integration of a photoswitch proton signal with the integration of the reference signal, assuming 1% error associated with the NMR integration. The stock solution was

stored under an argon atmosphere in the dark at −20 °C. The stock solution was diluted to the concentration range of 0.5–3.0 μM in HPLC-grade solvents or deionized water for spectroscopic measurement unless otherwise specified.

UV-vis absorption spectra were acquired on a Perkin Elmer Lambda 20 or Lambda 25 spectrometer using quartz glass cuvettes from Starna (10 mm path length). All measurements were conducted at 25 °C unless otherwise stated, with the temperature controlled by a water-cooled PTP-1 Peltier unit from Perkin Elmer.

Photoswitching experiments were conducted using Prizmatix collimated modular Mic-LED light sources including mic-LED-365L (centered at 365 nm, FWHM = 14 nm), mic-LED-405L (centered at 405 nm, FWHM = 18 nm), and mic-LED-630 (centered at 630 nm, FWHM = 15 nm) at the given power setting. The intensity of the light sources was controlled by a Benchtop LED Current Controller from Prizmatix. The power of the light source (in mW) was measured by a Coherent® PowerMax™-USB/RS Sensor System.

To measure the quantum yields of photoswitching processes, two LEDs were joined into one output beam via a beam combiner, which was coupled to a liquid light guide (diameter = 3 mm). The liquid light guide was wired close to the cuvette holder and orthogonal to the beamline of the spectrometer, as shown in Figure S1. The irradiation was performed using MATLAB script to control irradiation time, interval time, and the number of cycles of irradiation.

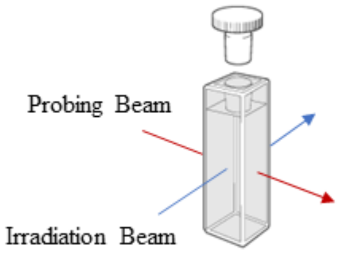

Figure S1. Schematic representation of the geometrical arrangement of the sample holder in the UV-vis spectrometer for measurements of the PSS spectrum, quantum yields of photochemical ring-closure and ring-opening, and fatigue resistance.

Fluorescence spectra were acquired at 298 K using an Edinburgh Instruments FS5 spectrofluorometer operating Fluoracle® software and equipped with a xenon arc lamp (providing 230–1000 nm excitation range), a thermostatic sample holder (SC-20) and both an R13456 PMT detector (200–950 nm spectral coverage, Hamamatsu) and an InGaAs analogue NIR detector (850–1650 nm spectral coverage). Quantum yields were measured either by an absolute method using an integrating sphere (SC-30) or by a relative method referenced to a Cy3B fluorescent dye.

S2. Synthesis of Compounds

This study investigates four novel photoswitchable fluorophores: **DAZ-RhoB**, **FULG-RhoX**, **DTE-Cy3**, and **DTE-Cy3B**. In the nomenclature system, the prefix specifies the type of organic photoswitch used: diazocine (**DAZ**), fulgimide (**FULG**), or dithienylethene (**DTE**). The suffix indicates the associated fluorescent dye: rhodamine (**RhoB**), Atto-590 (**RhoX**), cyanine 3 (**Cy3**), or a structurally rigid variant of Cy3 (**Cy3B**). These compounds are designed following a dyad strategy, in which the organic photoswitch is linked to a fluorophore. The photoswitch can exist in two states. In one state, the photoswitch quenches the fluorophore's emission through Förster resonance energy transfer (FRET), while in the other, it permits unimpeded fluorescence. This design allows for the controlled on-off switching of fluorescence, modulated by the state of the photoswitch.

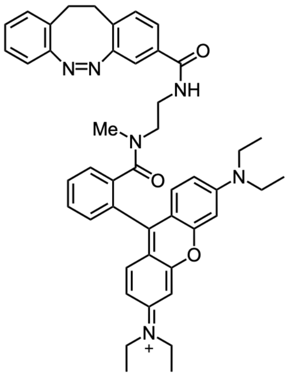

DAZ-RhoB

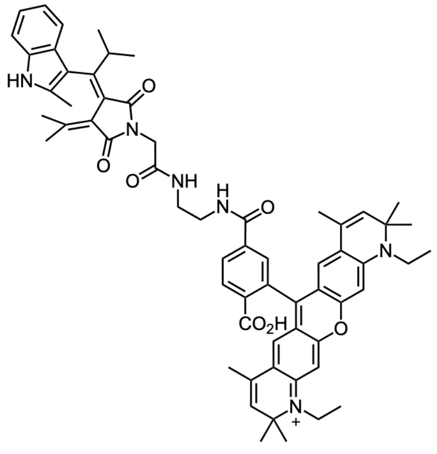

FULG-RhoX

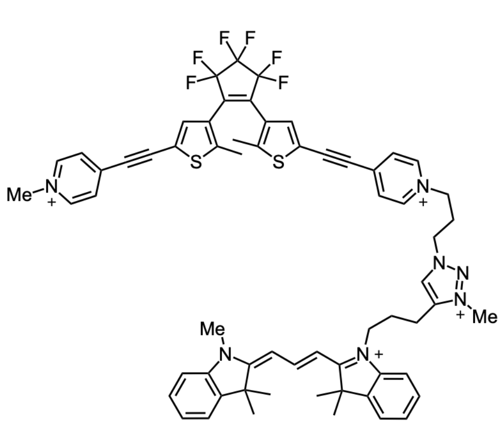

DTE-Cy3

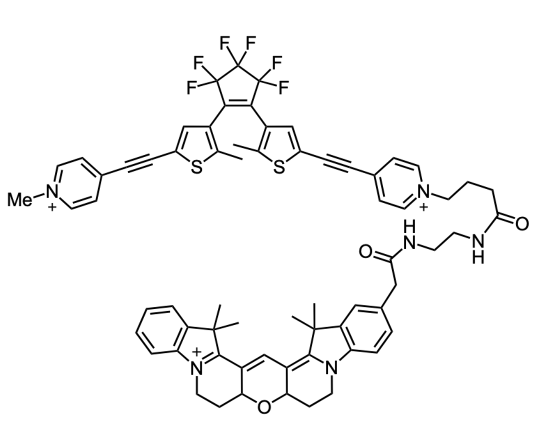

DTE-Cy3B

Figure S2. Summary of the four photoswitchable fluorophores covered in this study.

The synthesis of **DAZ-RhoB** was conducted by separately preparing its two main components: the diazocine photoswitch and the rhodamine dye (Scheme S1). The diazocine component, specifically the **DAZ**, was synthesized according to the established protocol by Maier and co-workers.[1] Concurrently, the rhodamine fluorophore was modified through the addition of a linker, resulting in **RhoB-NHBoc**. Before the final amide coupling reaction to form the **DAZ-Rho**, both precursors were subjected to deprotection processes. The DAZ-ester underwent treatment with LiOH to convert it into the free carboxylic acid, and the **RhoB-NHBoc** was treated with trifluoroacetic acid (TFA) to release the free amine. Following deprotection, the carboxylic acid was activated using HBTU. This activated intermediate was then coupled with the **RhoB-amine** in a one-pot reaction to yield the desired **DAZ-RhoB**.

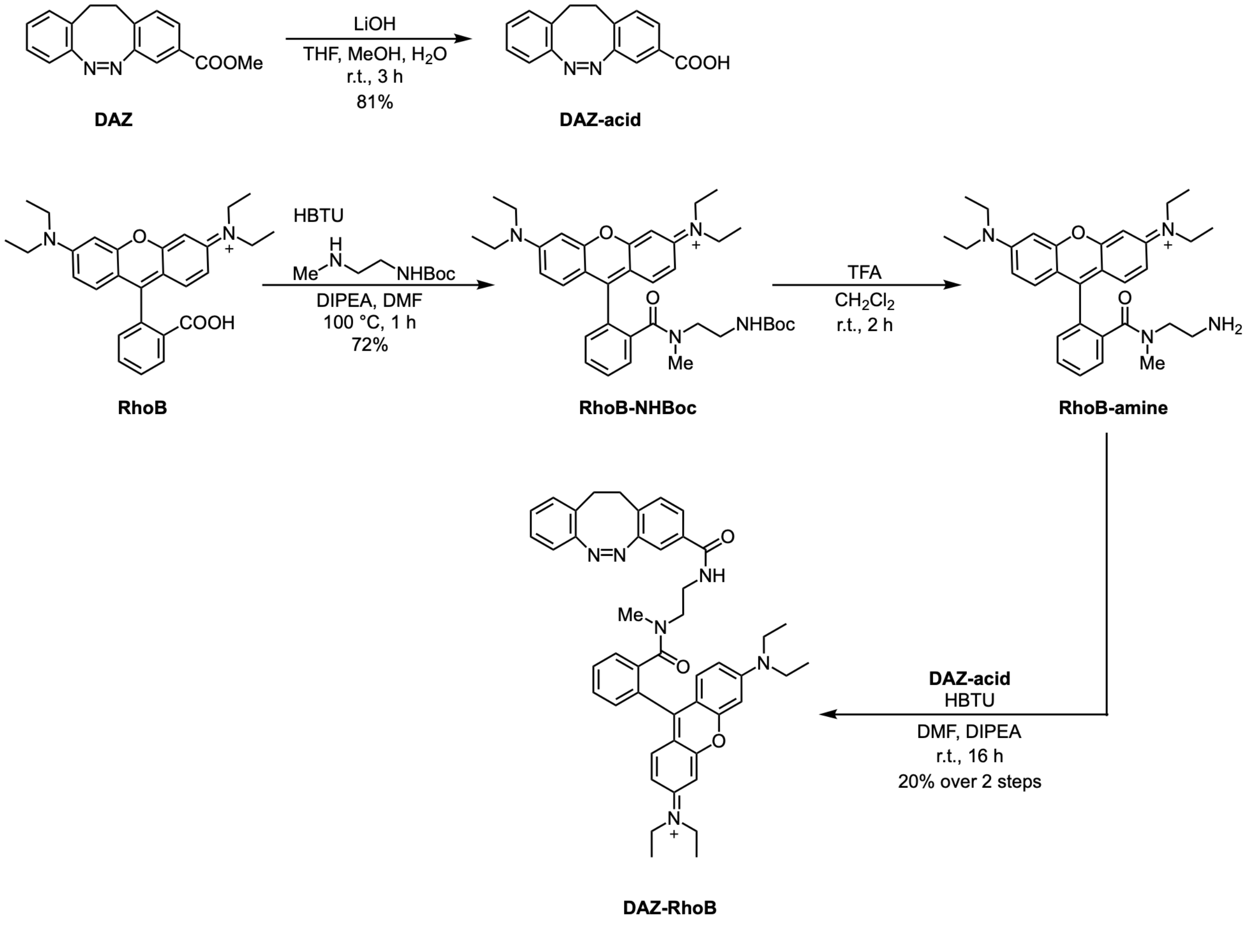

Scheme S1. Synthesis of photoswitch **DAZ** and the dyad **DAZ-RhoB**.

The synthesis of **FULG-RhoX** commenced with the preparation of the fulgimide photoswitch (Scheme S2). This photoswitch contains an indole heterocycle with an isopropyl group substituted at the bridge carbon, which enforces a conformation of the photoactive hexatriene unit favorable for efficient photocyclization. The fulgimide photoswitch was synthesized following the general strategy described by Lachmann and Simeth, with modifications to the functional groups.[2-4] The synthetic route began with a Stobbe condensation to generate an isopropylidenesuccinate intermediate **2**, followed by a second Stobbe condensation to construct the molecules **6** and **7** bearing hexatriene core. Subsequent ring closure led to the formation of a fulgide **10**, which was then converted to the corresponding fulgimide bearing a free carboxylic acid functionality. Conversion of fulgide **10** to **FULG** was found to be more efficient from the closed form.

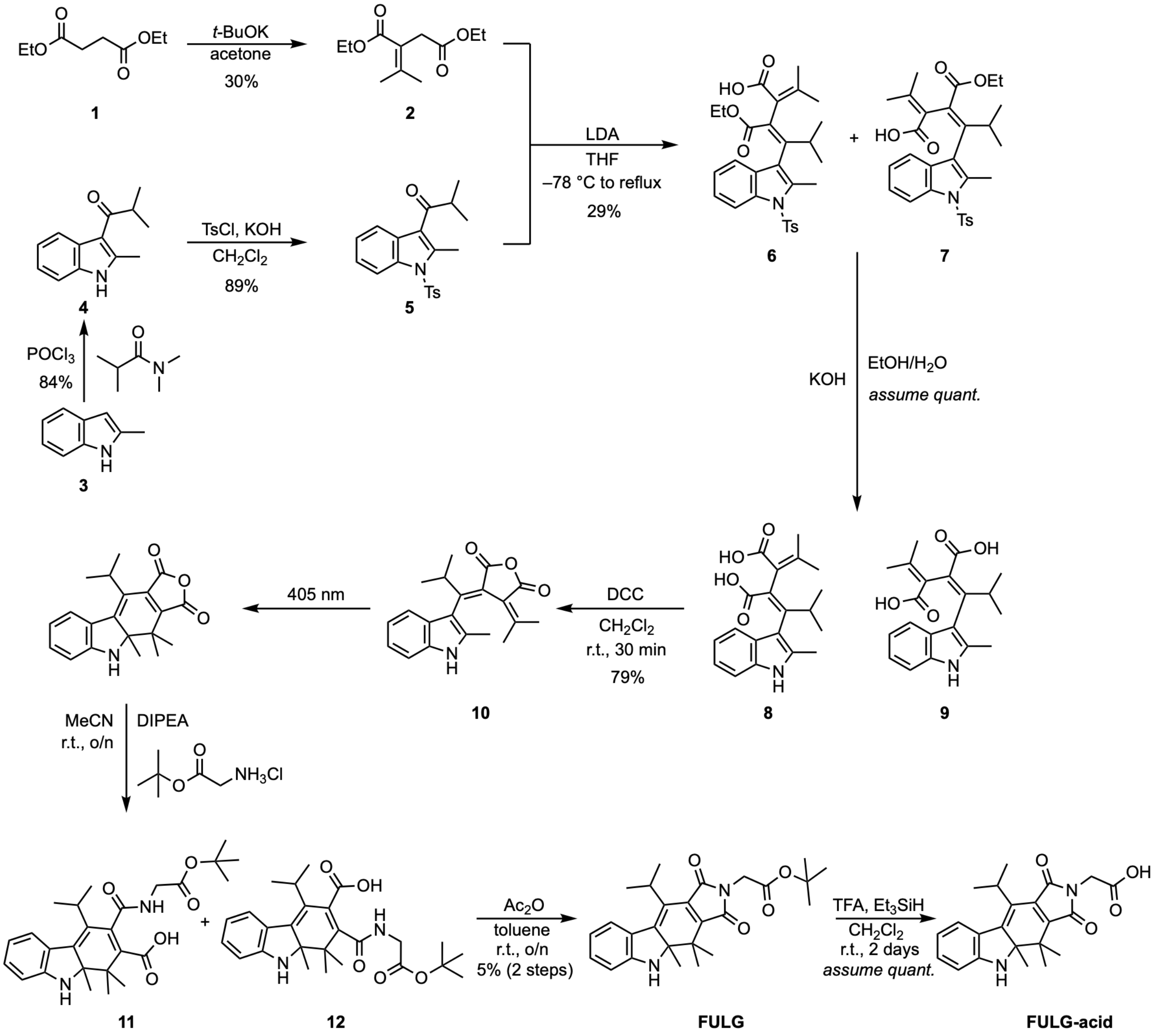

Scheme S2. Synthesis of the photoswitch **FULG**.

After testing the photoswitching properties, the **FULG** photoswitch was coupled to the fluorophore Atto-590 (RhoX) through an amide coupling reaction with HBTU activation (Scheme S3).

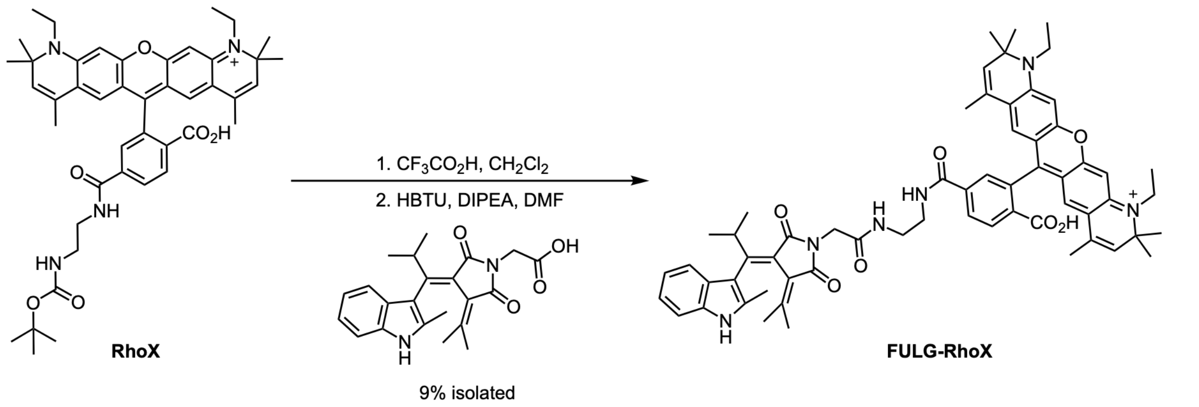

Scheme S3. Synthesis of **FULG-RhoX** dyad by amide coupling.

We further prepared two switchable dyads using a dithienylethene and cyanine 3 fluorophores. The key DTE intermediate **DTE-dipy** (see later Scheme S5) was prepared following a procedure described by Qiu and co-workers.[5] The Cy3 fluorescent dye was prepared by a step-wise approach, forming the hemicyanine **15** first, followed by forming **Cy3-alkyne** (Scheme S4).

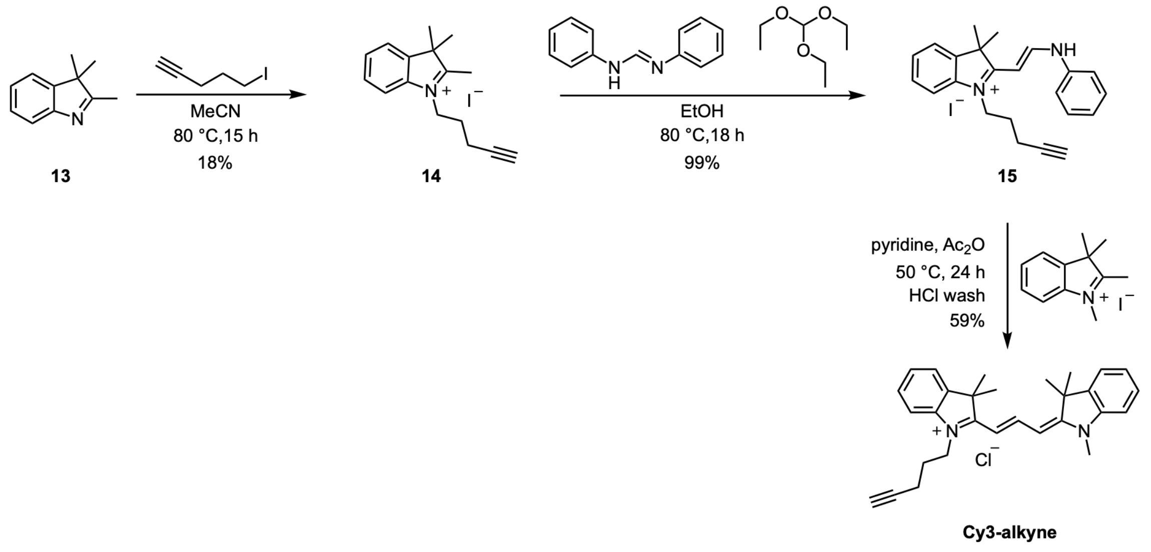

Scheme S4. Synthesis of **Cy3-alkyne** fluorescence dye.

Cy3-alkyne was converted into **Cy3-iodo** through a copper-catalyzed azide-alkyne cycloaddition (CuAAc) reaction followed by a nucleophilic substitution with **DTE-dipy** to form **DTE-py-Cy3**. The remaining free pyridine group was eventually methylated to form the final dyad **DTE-Cy3**. This nucleophilic substitution route was adopted because we found that CuAAc reaction does not proceed when we incorporate an azide group to the DTE core.

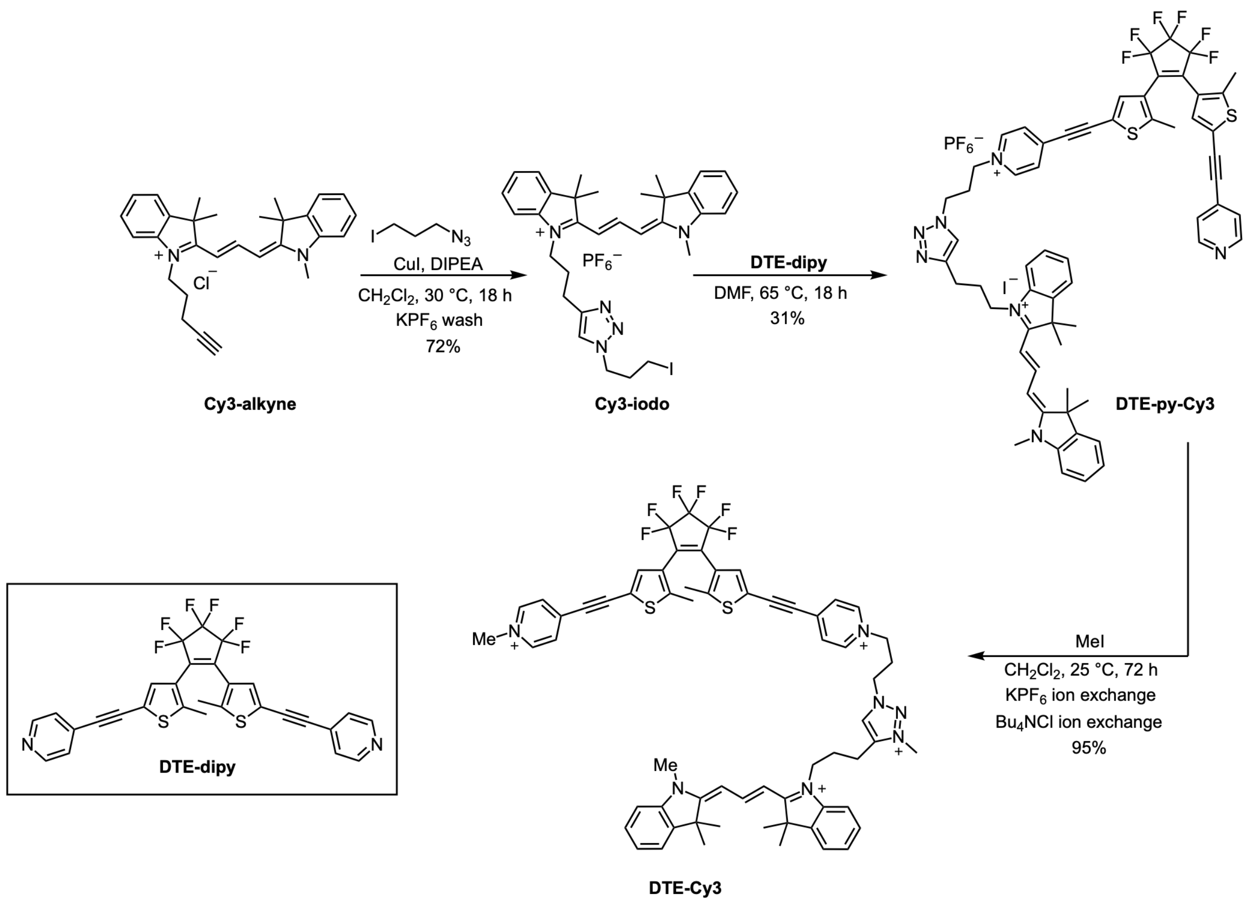

Scheme S5. Synthesis of **DTE-Cy3**.

The synthesis of **Cy3B** was carried out following the general Cy3B synthetic route developed by Hall and co-workers (Scheme 3.8),[6] with a modification to incorporate a carboxylic acid functionality on the indolenine aromatic ring. The synthesis begins with the preparation of indolenines **19** and **21**. Indolenine **19**, functionalized with a carboxylic acid group, was prepared starting from 2-(4-aminophenyl)acetic acid. This starting material was converted into hydrazine **17** using sodium nitrite and tin(II) chloride dihydrate.[7] Subsequently, indole **18**, bearing a carboxylic acid group, was synthesized through a Fischer indole synthesis.[8] Indolenines **19** and

21 were then obtained by alkylating the indole nitrogen with 2-(2-bromoethyl)-1,3-dioxolane. Since the stepwise cyanine synthesis provided higher overall yields compared to the one-pot approach in the previous Cy3 synthesis, a similar stepwise strategy was adopted for the synthesis of **Cy3B**. First, the hemicyanine intermediate **22** was prepared and isolated. Then, **22** was condensed with the other indolenine to form **23**, which further underwent cyclization under acidic conditions to yield the desired **Cy3B**. This synthetic route allowed us to easily prepare **Cy3B** on a half gram to gram scale.

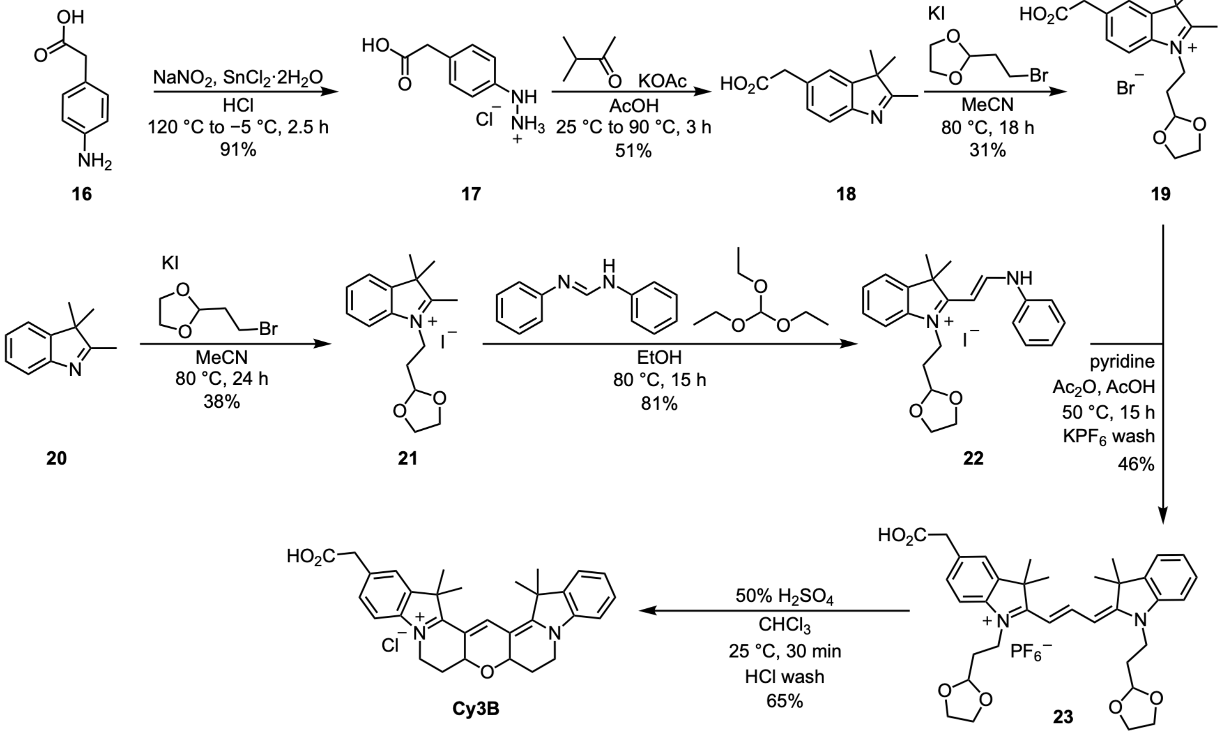

Scheme S6. Synthesis of **Cy3B**.

DTE-Cy3B was synthesized using amide coupling reactions with an ethylenediamine linker (Scheme S7). The synthesis began by converting **Cy3B** to **Cy3B-NHS ester**, which can easily react with nucleophiles. The ethylenediamine linker, with one end protected by a Boc group, was reacted with the NHS ester to form **Cy3B-NHBoc**. In parallel, the **DTE-dipy** was modified with an alkyl linker bearing a protected carboxylic acid, yielding **DTE-COO*t*Bu**. Both the *t*-butyl ester protecting group on the DTE and the Boc protecting group on the Cy3B were removed using trifluoroacetic acid separately. The deprotected DTE was then activated with HBTU and coupled

to the deprotected cyanine, which featured a free amine group. Without any purification, the coupled product was finally methylated with iodomethane to form **DTE-Cy3B**.

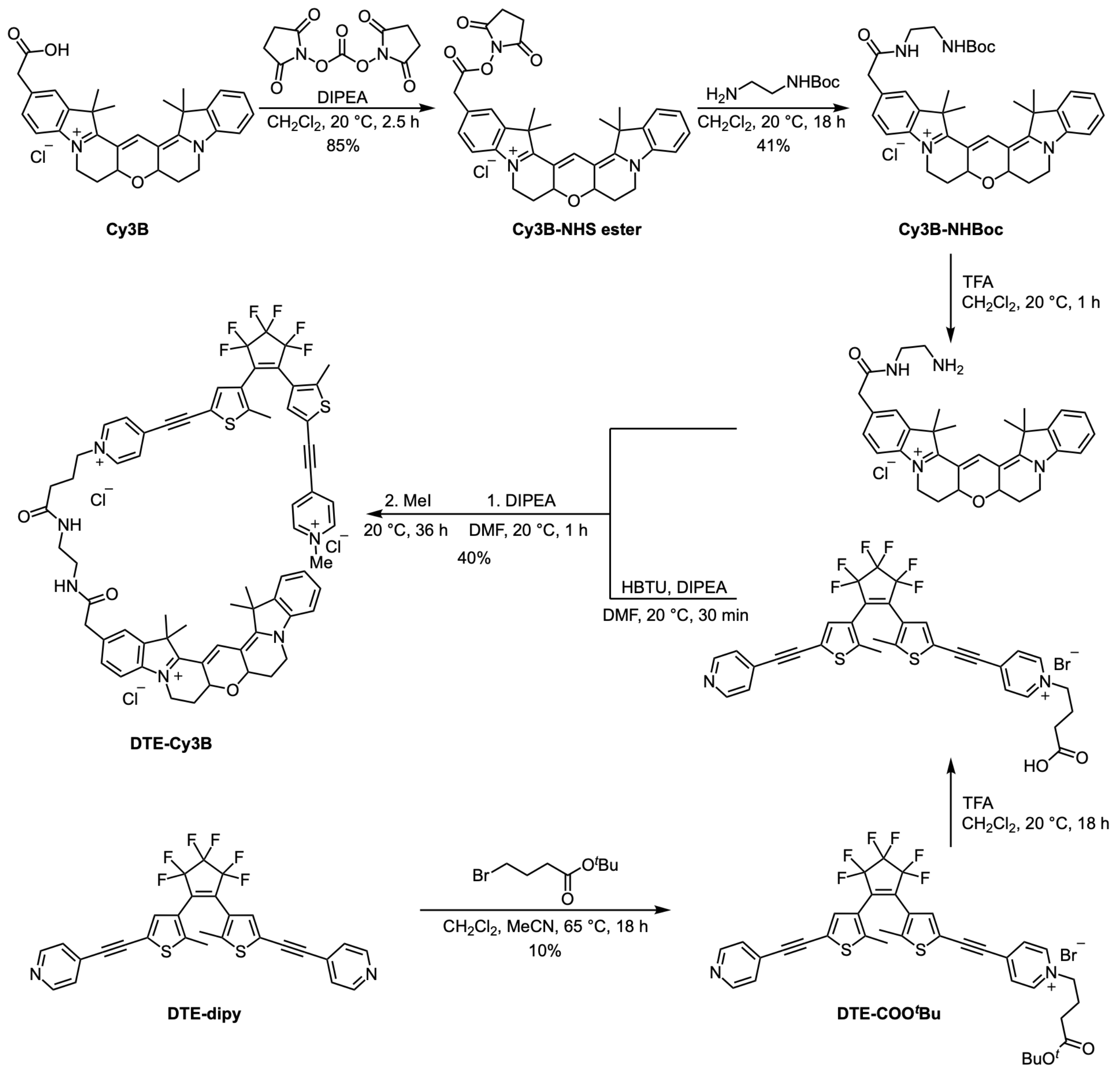

Scheme S7. Synthesis of **DTE-Cy3B**.

S3. UV-Vis Spectra of the Model Photoswitches

The photophysical and photochemical properties of each photoswitch were evaluated prior to their incorporation into the dyad systems. The model photoswitches used in these tests were capped or protected analogues, as depicted in Figure S3. The diazocine photoswitch (**DAZ**) was evaluated in its methyl ester form, the fulgimide photoswitch (**FULG**) as a *t*-butyl ester-protected derivative, and the dithienylethene photoswitch (**DTE**) as a methyl-capped analogue designed to mimic the dicationic character of the final dyad structure.

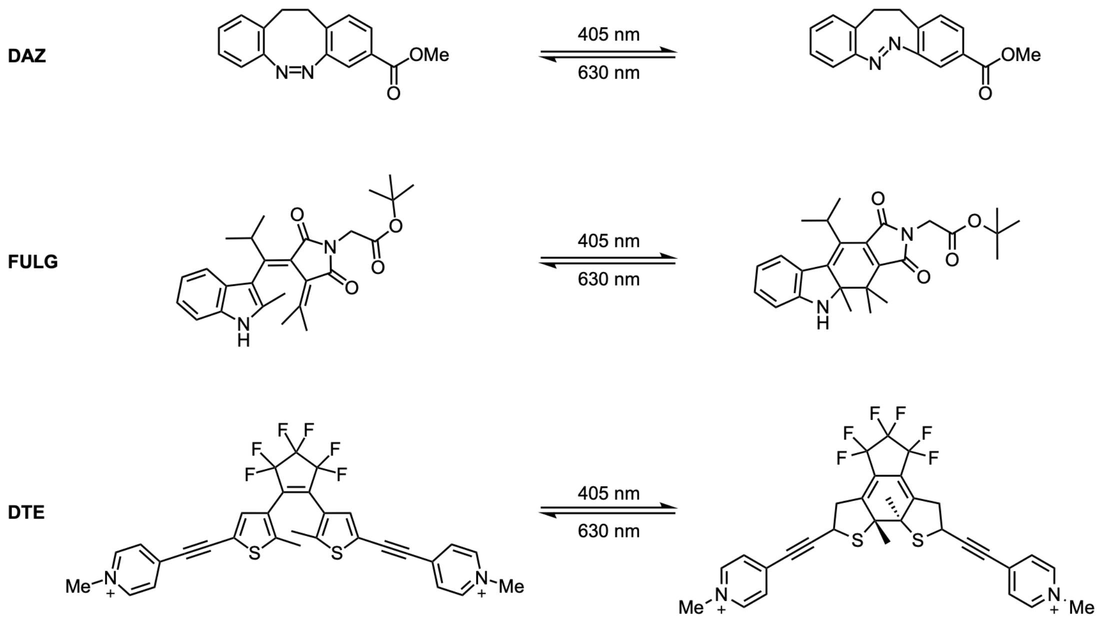

Figure S3. Model photoswitches for testing the photochemical and photophysical parameters.

A typical measurement is as follows: a stock solution of **DTE** in water is diluted to a have an absorption of less than 0.1. The sample was irradiated with 630 nm light (0.11 W, 1 min) to convert all residual closed **DTE** into open **DTE**. The open UV-vis spectrum was recorded between 300 nm and 1100 nm. The solution of **DTE** was then irradiated with cycles of 405 nm LED (18.9 mW, 1 s irradiation, 3 min interval) and the UV vis spectrum was recorded during the intervals until no further changes in the UV-vis spectrum were observed. The final UV-vis spectrum with no further change after irradiation is the absorption spectrum of the photostationary state (PSS). The UV-vis spectrum of the closed DTE was then calculated based on the fact that the absorption spectrum of the PSS at a certain wavelength is a linear combination of open-form absorption and the closed-

form absorption. The composition of the photoswitch solution at PSS under 405 nm light irradiation was measured by ^{1}H NMR spectroscopy as detailed in Section S6.

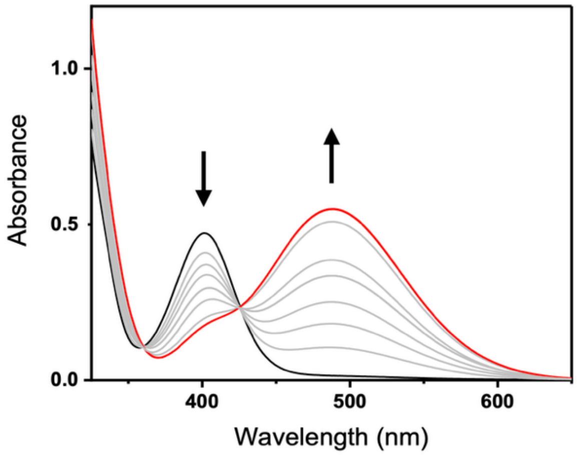

Figure S4. Photoswitching UV-vis of **DAZ** measured in DMSO at 25 °C. The red line corresponds to a PSS mixture containing 72% of *E*-isomer.

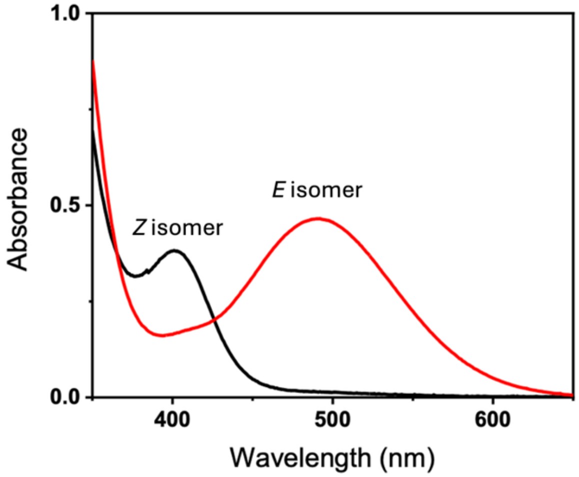

Figure S5. UV-vis absorption of **DAZ** in its *Z* isomer (black) and *E* isomer (red) measured in DMSO at 25 °C.

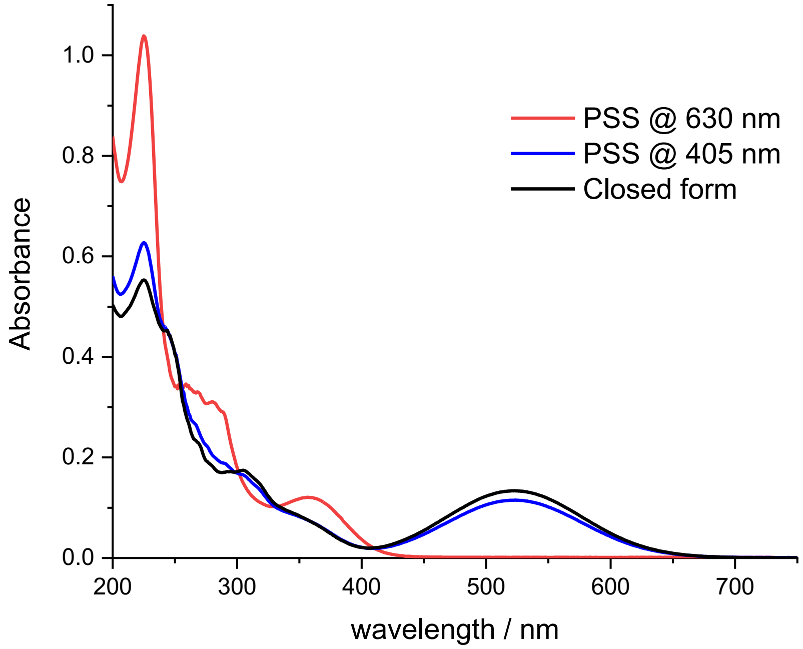

Figure S6. UV-vis absorption of **FULG** in its open for (red), at PSS (blue) and in its closed form (black), measured in MeCN at 25 °C.

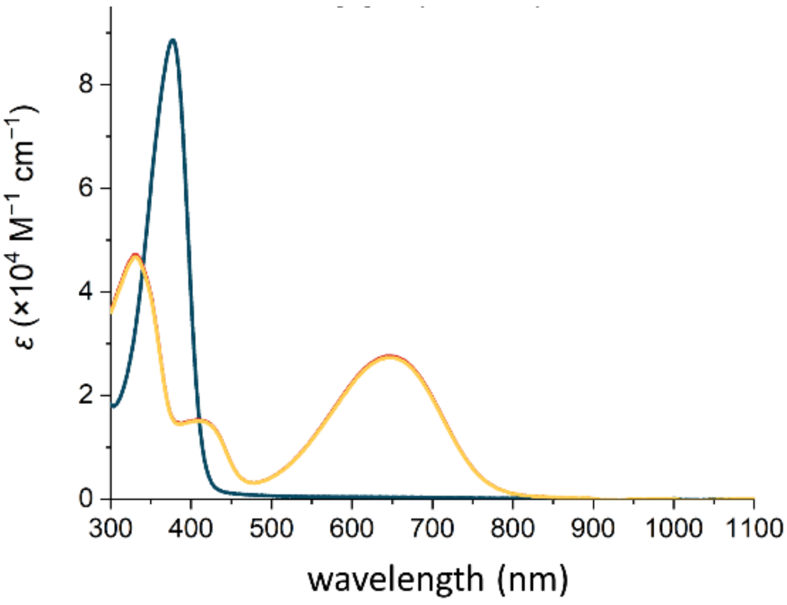

Figure S7. UV-vis absorption of **DTE** in its open form (blue), at PSS (yellow), and in in its closed form (red) measured in water at 25 °C.

S4. Determination of Molar Absorption Coefficients

The concentration of the stock solution was determined by ^{1}H NMR spectroscopy by using a known concentration of methanol as a reference. An NMR relaxation delay of 30 s was used to measure accurate integrals, as detailed in Section S1. A typical experiment for determining the molar absorption coefficient at the band maximum is as follows: a stock solution of DTE in D_2O containing 2.24 mM methanol was prepared and the exact concentration of **DTE** was determined by NMR to be 1.93 mM. The stock solution of **DTE** was diluted into water and the absorption spectra of various concentrations were recorded following irradiation with 630 nm light at 112.0 mW for 5 minutes. The absorbance at 377 nm was plotted against concentration to give a graph of the form $y = mx$ where m gives the molar absorption coefficient according to the Beer-Lambert Law.

S5. Photoswitching Quantum Yields

Photoswitching quantum yields were determined using the initial-slope method we used previously.[5] To accurately determine the initial slope (*m*) of the photochemical process, the power of the LED light source was turned down sufficiently that the formation of the product is linear (only 5%–10% of the product is formed during the process of measurement). A typical photoswitching quantum yield experiment is as follows: an aqueous solution of **DTE** (1.6 μM) was placed in a cuvette in the sample holder of the UV-vis spectrometer and stirred at 25 °C. After irradiating the sample with 405 nm light (18.9 mW, 200 ms irradiation), the absorption intensity at 650 nm corresponding to peak maximum of the formed product was recorded. The irradiation-recording process was repeated for five cycles to acquire a kinetic trace of the photochemical conversion (7% of the open-form **DTE** was converted to the closed-form). Initial slope measurement was carried out three times for each compound, showing good reproducibility. An example of the initial slope measurement is shown in Figure S8.

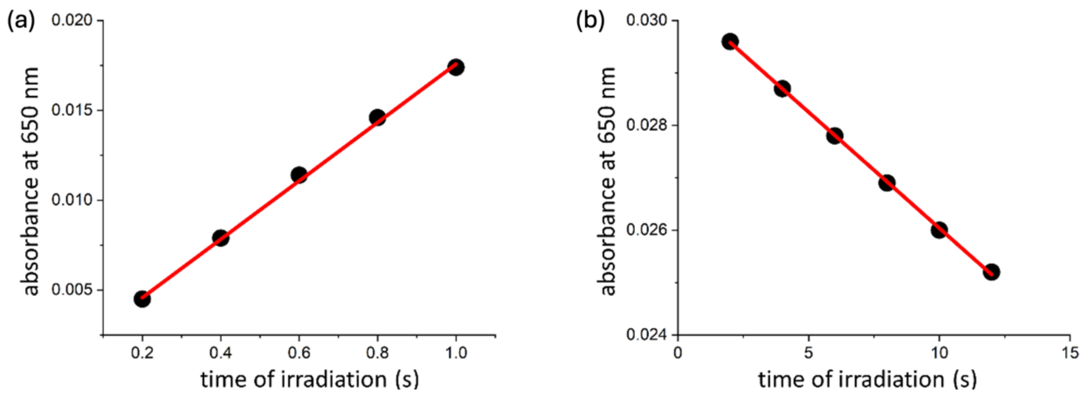

Figure S8. The initial region of the kinetic traces of (a) photocyclization and (b) photoreversion in water of DTE and their linear fits. The gradients *m* of (a) 0.0172 ± 0.0007 s^{-1} and (b) 0.00045 ± 0.00001 s^{-1} were used in the calculation of photochemical quantum yields.

Equation S1 was then used to calculate the photoswitching quantum yield, where φ is the photochemical quantum yield of the reaction, m is the slope of the linear fit for the time-dependent absorbance change during irradiation for appearance or disappearance of closed form DTE monitored at the band maxima in the visible region (e.g. monitored at 650 nm for **DTE**), V is the volume of solution under irradiation, N_A is the Avogadro constant, h is the Planck constant, c is the speed of light, P is the irradiation power (in W), λ is the excitation wavelength, A is the absorbance at excitation wavelength, ε_{prod} is the molar absorption coefficient of the formed product

at the monitoring wavelength (e.g. at 650 nm for **DTE**), *l* is the pathlength (1 cm). The photoswitching quantum yield results of different photoswitches are shown in Table S1.

$$\varphi = \frac{m \cdot V \cdot N_\mathrm{A} \cdot h \cdot c}{P \cdot \lambda \cdot (1 - 10^{-A}) \cdot \varepsilon_\mathrm{prod} \cdot l} \quad (S1)$$

Table S1. Photoswitching quantum yields of the model photoswitches.

	$\Phi_{s,1}$ (%)	$\Phi_{s,-1}$ (%)
**DAZ**[a]	15	35
**FULG**[b]	21	4.0
**DTE**[c]	17	0.12

[a]Measured in DMSO. [b]Measured in MeCN. [c]Measured in water.

S6. Model Photoswitch PSD Determination by ^{1}H NMR

The percentage conversion at the PSS, or the photostationary state distribution (PSD), of each compound was measured by comparing the ^{1}H NMR spectrum of the non-quenching form of the photoswitch with quenching form of the photoswitch. All NMR spectra here were recorded with extended (10 s) relaxation time. The aromatic proton signals were integrated and used to determine the relative percentage of the two isomers. A typical procedure is as follows: a sample of **DTE** in D_2O was irradiated with 630 nm light at 112.0 mW for 3 minutes and then the NMR spectrum of the non-quenching form was recorded. The sample was then irradiated with 405 nm light at 159 mW for 5 minutes and then the NMR spectrum was recorded. Irradiation with 405 nm light for 5 minutes followed by taking NMR spectrum was repeated for 7 times until no further change in the NMR spectrum was observed. The integration ratios of the peaks H(a):H(a') and H(b):H(b') at the PSS were measured to give a PSD of 96%. The uncertainty associated with NMR integration is evaluated by setting the integration of H(a) peak to 2.00 and randomly integrate on the baseline where no peak occurs (with the width of 0.05 ppm) for ten times, giving average uncertainty of ± 0.02, which corresponds to 1% error in the integration value.

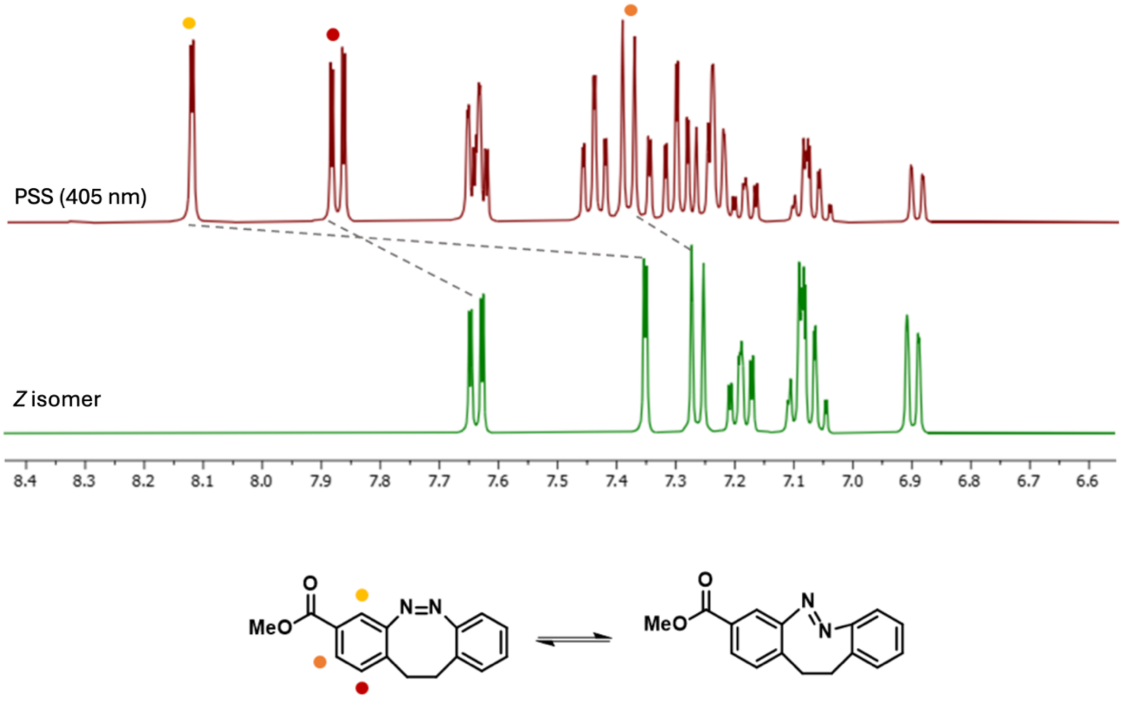

Figure S9. ^{1}H NMR spectra (500 MHz) of non-quenching **DAZ** (middle), 72% ± 1% conversion reached at PSS under 405 nm irradiation (top) in DMSO and the scheme demonstrating the photoswitching process (bottom).

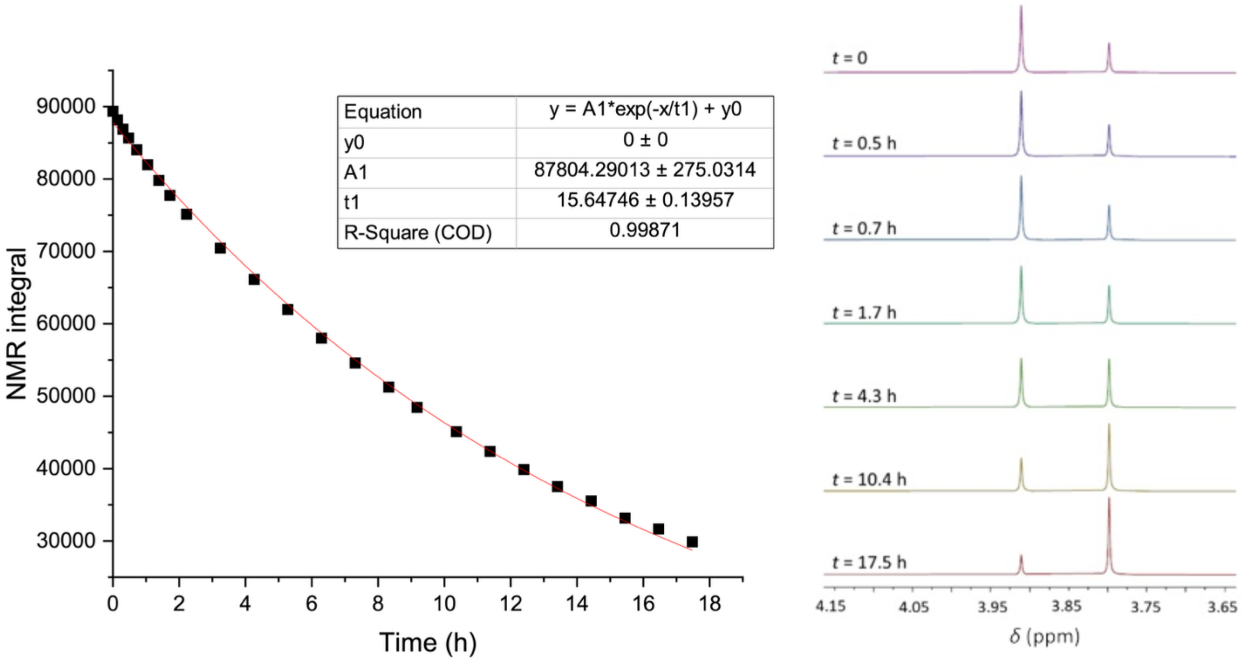

Figure S10. Thermal relaxation (measured by NMR) of *E*-**DAZ** switch (left); $k = 0.0638\ h^{-1}$ and $t_{½} = 10.9$ h. Selected ^{1}H NMR (500 MHz) spectra of methyl ester proton change over time (right). Spectra recorded at 298 K in DMSO.

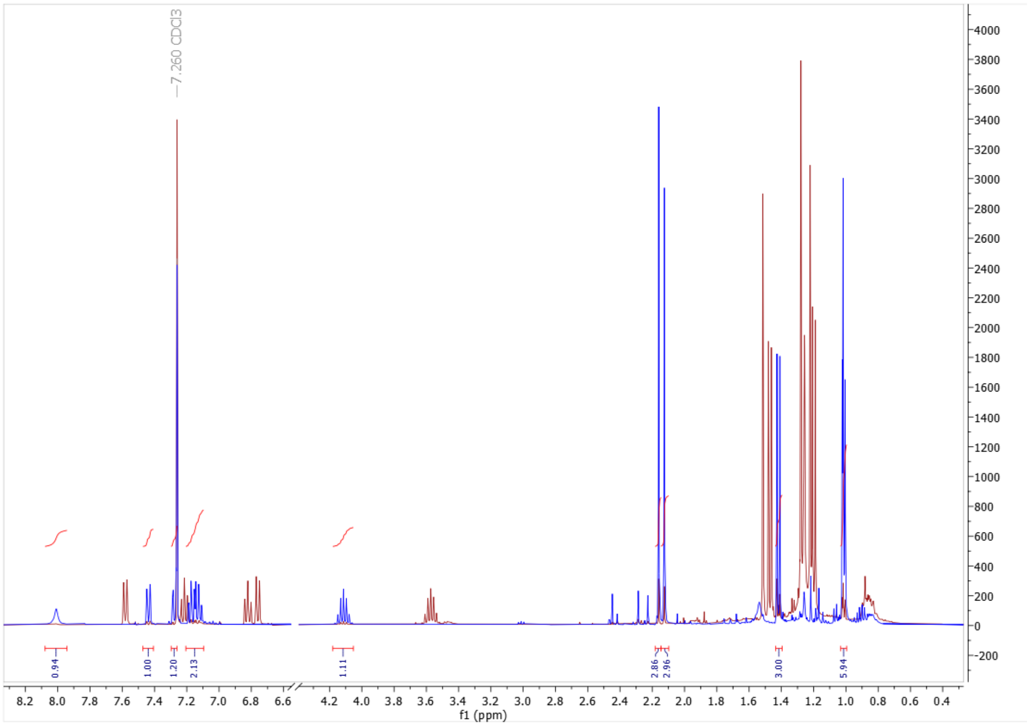

Figure S11. ^{1}H NMR spectrum (500 MHz) of non-quenching **FULG** (blue), 87% ± 1% conversion reached at PSS under 405 nm irradiation (red) in $CDCl_3$.

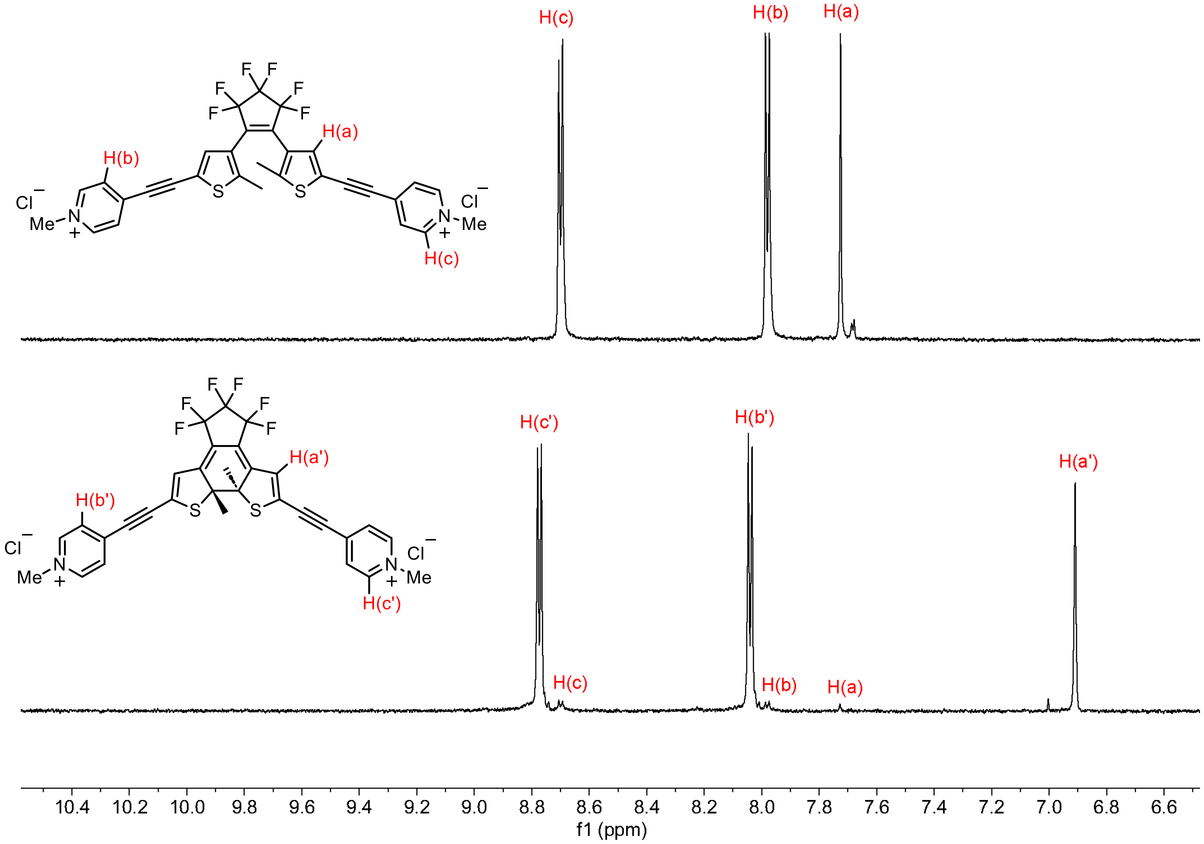

Figure S12. ^{1}H NMR spectra (500 MHz) of non-quenching **DTE** (top) and 96% ± 1% conversion reached at PSS under 405 nm irradiation (bottom) in D_2O.

Table S2. PSD of model photoswitches measured by ^{1}H NMR.

	$PSD_{n\text{-}q}$ (%)	$PSD_{q\text{-}n}$ (%)
**DAZ**[a]	72	100 (525 nm)
**FULG**[b]	87	100 (630 nm)
**DTE**[c]	96	100 (630 nm)

[a] Measured in DMSO-d_6. [b] Measured in $CDCl_3$. [c]Measured in D_2O.

S7. Dye Selection

For effective fluorescence modulation in a photoswitchable dyad, the selected fluorophore must satisfy a key spectroscopic criterion to enable efficient Förster resonance energy transfer (FRET)-based quenching. Specifically, there must be substantial spectral overlap between the emission spectrum of the fluorophore (donor) and the absorption spectrum of the quenching state of the photoswitch (acceptor). This overlap is essential for efficient energy transfer, allowing the quenching state of the photoswitch to quench the fluorophore's emission effectively. As a result, the system exhibits a pronounced difference in fluorescence intensity between the "on" (non-quenching) and "off" (quenching) states of the photoswitchable fluorophore.

To identify fluorophores that fulfill these criteria, an extensive survey was performed, covering both published literature and products from leading chemical suppliers (including AAT Bioquest, ATTO-TEC, Lumiprobe, Merck, Thermo Fisher Scientific, and Vector Laboratories). The photophysical parameters (including absorption maxima, emission maxima, and fluorescence quantum yields) of the main fluorophore families were collated and are presented in the Table S3.

Table S3. Notable fluorescent dyes and their fluorescent properties.

Dye	Excitation Maximum (nm)	Emission Maximum (nm)	Fluorescent Quantum Yield (%)	Solvent
Atto-532[a,*]	532	552	90	water
Atto-550[a,*]	554	576	80	water
BODIPY-FL[b]	502	511	90	methanol
Cy2[b]	492	508	12	PBS
Cy3[b]	555	569	15	PBS
Cy3B[b]	560	571	58	PBS
Cy3.5[c]	580	610	35	ethanol
Cy5[c]	646	662	20	ethanol
Cy5.5[c]	684	710	20	ethanol
Cy7[c]	750	773	30	ethanol
Rho-110[d,e]	498	520	92	ethanol
Rhodamine 6G[b]	525	548	95	water
Rhodamine B[d,e]	553	627	65	ethanol
Rhodamine-X[b]	578	604	94	PBS
Rhodamine-Si[f]	648	663	34	PBS
Fluorescein	498	517	79	ethanol

[*]Structurally related to rhodamine 6G. [a]Data from ATTO-TEC. [b]Data from AAT Bioquest. [c]Data from Lumiprobe. [d]Data from Merck. [e]Reported by Kubin and co-workers.[9] [f]Reported by Rao and co-workers.[10]

Based on this survey, Rhodamine B (**RhoB**) was selected for **DAZ**, atto-590 (**RhoX**) was selected for **FULG**, and **Cy3** and **Cy**3B were selected for **DTE** due to good FRET overlap.

S8. FRET Efficiency Modelling

Before synthesizing and testing the dyad system, the FRET-based fluorescent quenching was modelled. FRET is the process by which the energy of an excited state donor fluorophore is non-radiatively transferred to an acceptor molecule. The requirements for FRET quenching to occur include physical proximity, the alignment of the donor and acceptor transition dipole moments, and spectral overlap between the FRET donor emission and the FRET acceptor absorption. The FRET efficiency (*E*) is defined as the quantum yield of the FRET process and can be calculated for a given donor and acceptor pair using Equation S2.

$$E = \frac{k_{\mathrm{ET}}}{k_{\mathrm{FL}} + k_{\mathrm{ET}} + \sum k_{\mathrm{i}}} \qquad \text{(S2)}$$

where k_{ET} is the rate of energy transfer from the donor to the acceptor, k_{FL} is the radiative decay rate of the donor, and k_{i} is the rate of any other non-radiative relaxation pathways excluding the energy transfer. FRET efficiency depends on the donor-to-acceptor separation distance (*r*) with an inverse 6th-power relationship shown in Equation S3.

$$E = \frac{1}{1 + \left(\frac{r}{R_0}\right)^6} \qquad \text{(S3)}$$

The Förster radius R_0 term is the distance at which FRET is 50% efficient and can be approximated with Equation S4:

$$R_0 = 0.2108 \left[\frac{\kappa^2 \cdot \Phi_{\mathrm{D}} \cdot J(\lambda)}{n^4}\right]^{\frac{1}{6}} \qquad \text{(S4)}$$

where Φ_{D} is the fluorescence quantum yield of the donor, and *n* is the refractive index of the solvent. The term κ^2 is the dipole orientation factor, which describes the relative orientation of the transition dipoles of the donor and acceptor. The value can range from 0 to 4, (0 for perpendicular,

1 for parallel, 4 for antiparallel) but is assumed to be an average of 0.667 given free rotation between the donor and acceptor. The term $J(\lambda)$ is the spectral overlap integral, which is given by Equation S5:

$$J(\lambda) = \int f_{\mathrm{D}}(\lambda) \cdot \varepsilon_{\mathrm{A}}(\lambda) \cdot \lambda^4 \cdot d\lambda \tag{S5}$$

where f_{D} is the normalised donor emission spectrum, ε_{A} is the acceptor molar absorption coefficient at wavelength λ.

Table S4 summarizes the parameters used to calculate the Förster radius and the corresponding results, assuming a dipole orientation factor of 0.667. The spectral overlap integral is calculated using the absorption spectrum of quenching state of the photoswitch and the emission spectra of the fluorescent dye.

Table S4. Förster distance of distance of different dyads calculated from the overlap integral.

System	$\boldsymbol{J(\lambda)}$ (mol^{-1} dm^3 cm^{-1} nm^4)	$\boldsymbol{\Phi_{\mathrm{D}}}$ (%)	$\boldsymbol{n}$	$\boldsymbol{R_0}$ (Å)
**DAZ-RhoB**	3.22×10^{13}	65	1.33	21.2
**FULG-RhoX**	1.18×10^{14}	86		35.2
**DTE-Cy3**	1.81×10^{15}	15		41.4
**DTE-Cy3B**	1.85×10^{15}	58		53.1

* The spectral overlap integral is calculated using the closed DTE-diMe absorption spectrum measured in the previous chapter, and Cy3 and Cy3B emission spectra supplied by AAT Bioquest.

To estimate the distance between the photoswitch and the dye, we constructed a molecular model for each of the dyads. The molecular models were optimized using the MMFF94 force field, forcing the linker group to adopt a fully extended conformation. The distance between the photoswitch and the fluorescent dye is then measured.

The FRET efficiencies for the dyads were calculated using the donor-acceptor distances estimated from the molecular models. The results are presented in Table S5. It is important to note that these molecular models were constructed assuming that the molecules adopt fully extended conformations. In reality, the synthesized molecules are expected to have higher FRET

efficiencies when the linkers randomly fold up in solution due to their flexibility, bringing the donor and acceptor closer together.

Table S5. Calculated FRET efficiency of the dyads.

Model	$\boldsymbol{R_0}$ (Å)	Maximum D-A Distance (Å)	FRET Efficiency (lower limit)
**DAZ-RhoB**	21.2	10.3	94.3%
**FULG-RhoX**	35.2	17.6	98.5%
**DTE-Cy3**	41.4	24.3	96.1%
**DTE-Cy3B**	53.1	28.2	97.8%

The FRET quenching model demonstrates that the quenching states of the photoswitches will effectively quench the fluorescence in the dyad. In contrast, when the photoswitch is in the non-quenching form, it will not quench the fluorescence, as there is no significant spectral overlap.

S9. Dyad UV-vis Absorption and Fluorescence

Following the successful synthesis of **DAZ-RhoB**, **FULG-RhoX**, **DTE-Cy3** and **DTE-Cy3B**, the UV-vis absorption spectra and the emission spectra were measured for each dyad when the photoswitch is in the non-quenching state (Figure S13–S16).

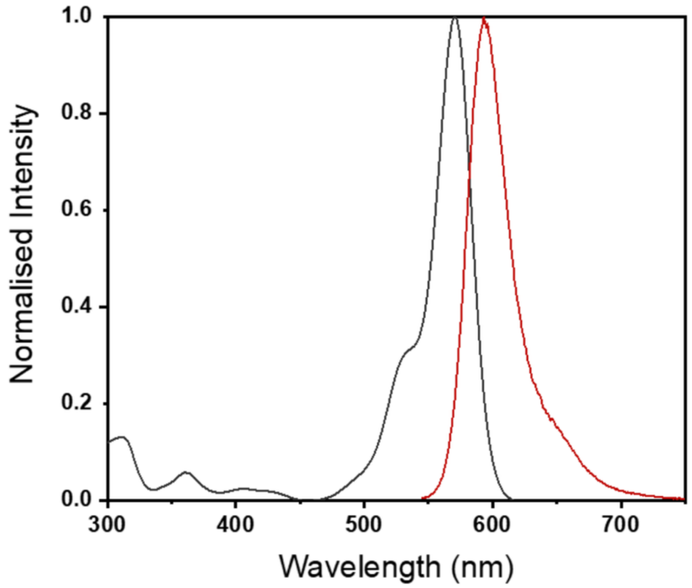

Figure S13. UV-vis absorption (black) and emission spectra of **DAZ-RhoB** measured when the photoswitch is in the non-quenching state in DMSO.

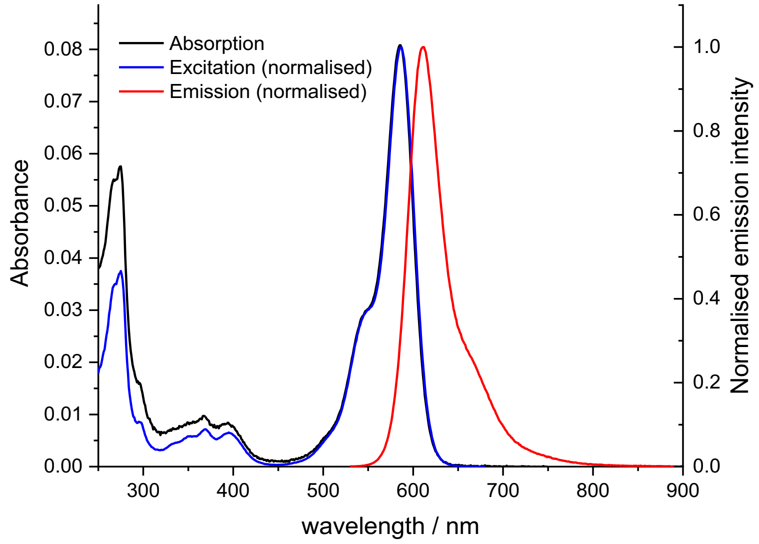

Figure S14. UV-vis absorption (black), emission spectra (red) and excitation spectra (blue) of **FULG-RhoX** measured when the photoswitch is in the non-quenching state in MeCN.

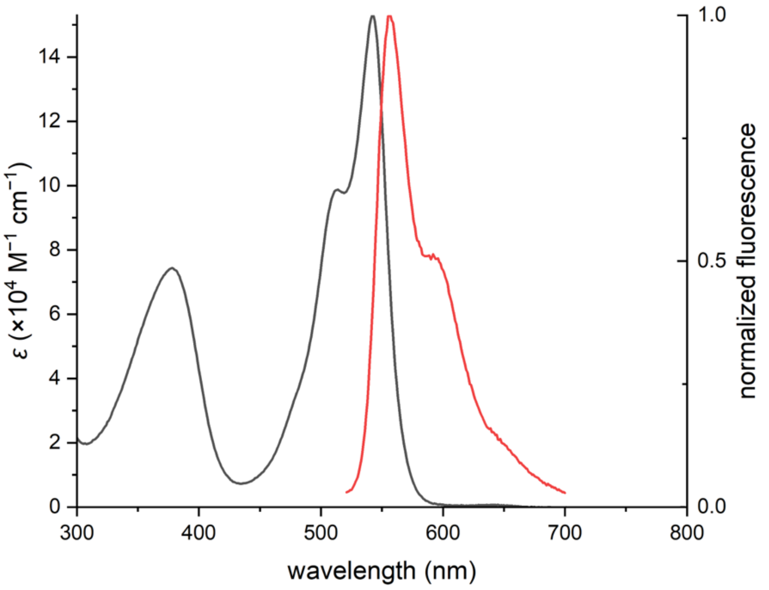

Figure S15. UV-vis absorption (black) and emission spectra (red) of **DTE-Cy3** measured when the photoswitch is in the non-quenching state in water.

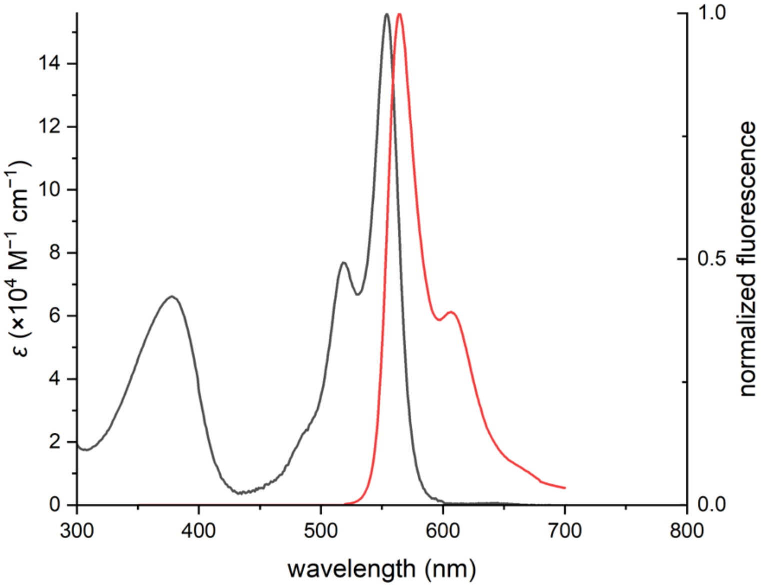

Figure S16. UV-vis absorption (black) and emission spectra (red) of **DTE-Cy3B** measured when the photoswitch is in the non-quenching state in water.

S10. Fluorescence Quenching

To evaluate whether photoswitching in the dyad leads to fluorescence quenching via FRET, fluorescence measurements were conducted before and after photoisomerization of the photoswitch. Initially, the dyad was tested in its non-quenching state, followed by irradiation at 405 nm to induce switching to the quenching state, and fluorescence intensity was measured again. A representative procedure is as follows: A solution of **DTE-Cy3** was prepared at a concentration low enough to ensure the absorbance of the cyanine dye was below 0.1. The sample was first irradiated with 630 nm light for 10 minutes to generate the non-quenching of the photoswitch. The fluorescence emission of the dyad was then recorded. Subsequently, the sample was irradiated with 405 nm light for 30 seconds to induce switching to the off state, followed by another fluorescence measurement. To assess reversibility, the sample was irradiated again at 630 nm for 30 minutes, and the fluorescence signal was measured once more. The fluorescence quenching spectra are shown in Figure S17–S19.

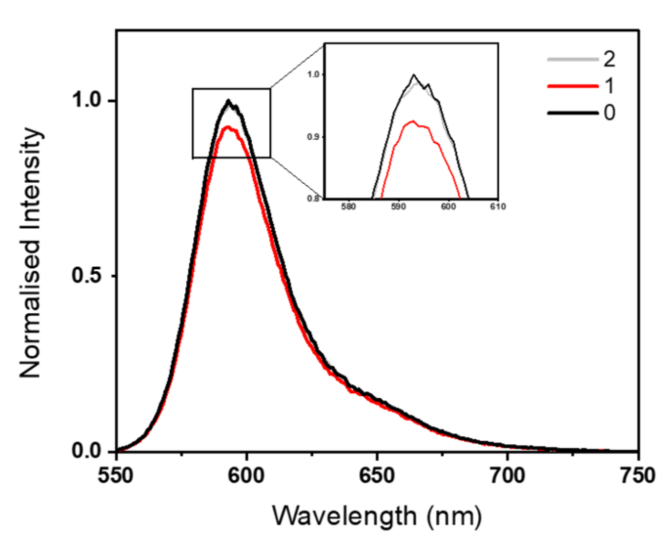

Figure S17. Normalized fluorescence signal of **DAZ-RhoB** in DMSO (non-quenching, 0, black), after irradiation with 405 nm light (quenching, 1, red) and after irradiation with 525 nm (restored, 2, grey).

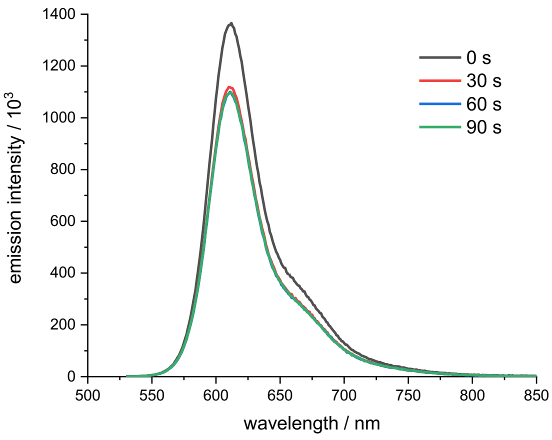

Figure S18. Fluorescent emission signal of **FULG-Atto** measured in MeCN at 0 s irradiation (when the photoswitch is in the non-quenching state) and after irradiation with 405 nm light.

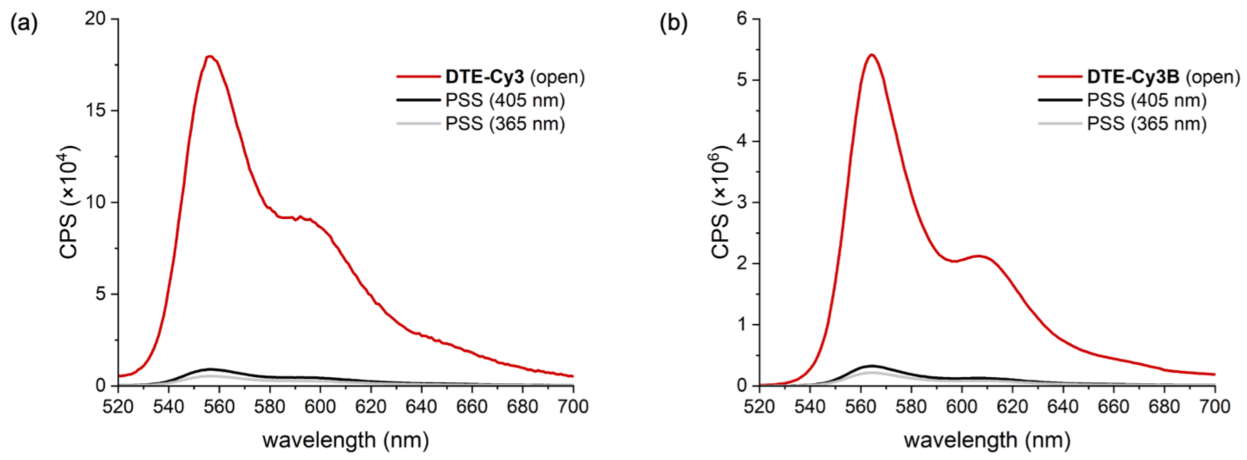

Figure S19. Fluorescent emission signal of (a) **DTE-Cy3** and (b) **DTE-Cy3B** measured in water when the DTE is in the non-quenching state (red), at PSS (405 nm, black) and at PSS (365 nm, grey).

Table S6. Percentage of fluorescence quenching between the on and off states measured by peak integration.[a]

	% quenching
**DAZ-Rho**[b]	7
**FULG-Atto**[c]	19
**DTE-Cy3**[d]	94 (97 at 365 nm)
**DTE-Cy3B**[d]	93 (96 at 365 nm)

[a]Under irradiation art 405 nm (unless stated otherwise). [b]Measured in DMSO. [c]Measured in MeCN. [d]Measured in water.

S11. PSD of Photoswitch in the Dyad by NMR

The PSD of the photoswitch after being incorporated into the switchable fluorophore was measured by ^{1}H NMR spectroscopy using the same approach described in Section S6. The new PSD of the systems under 405 nm irradiation are summarized in Table S7.

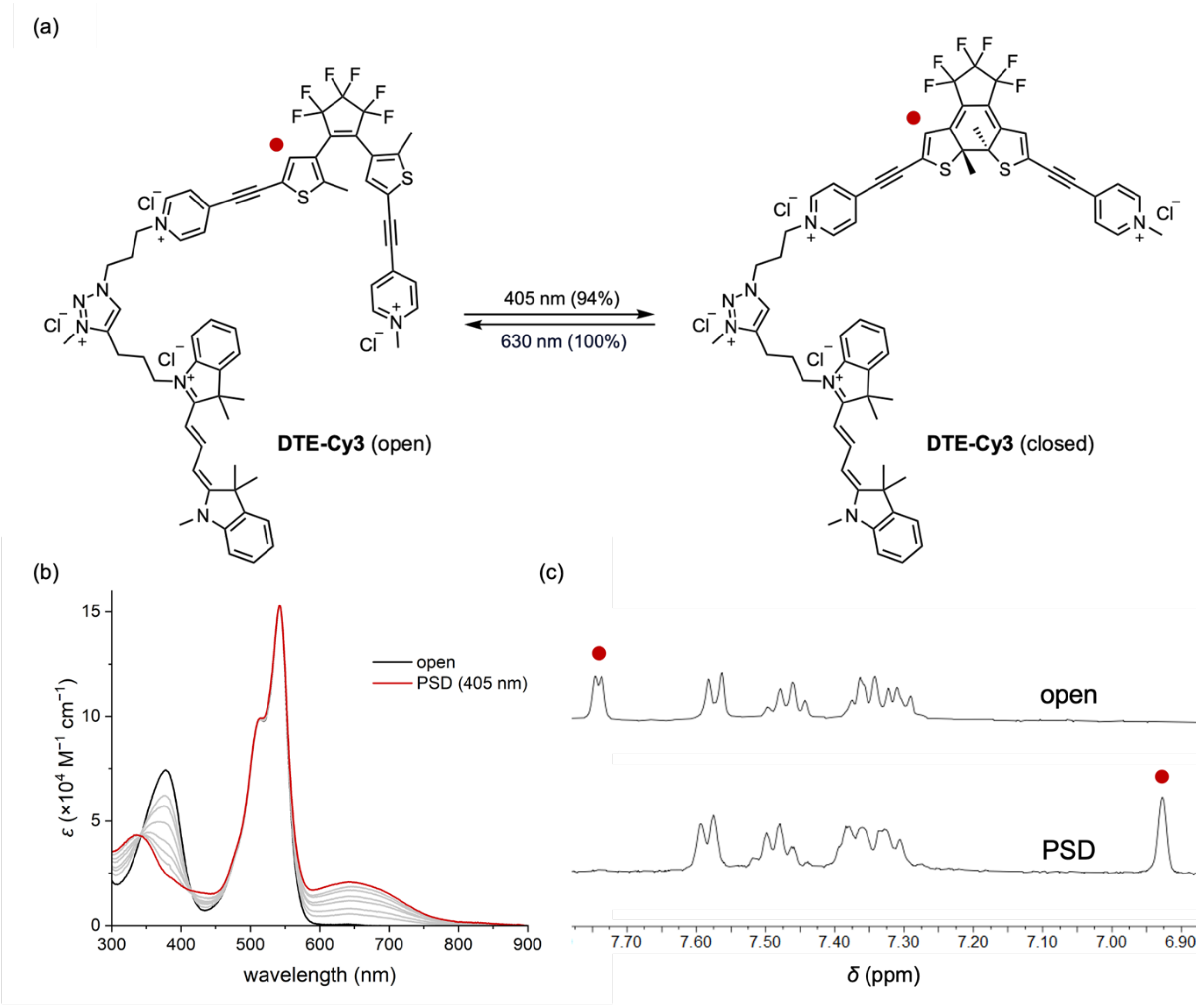

Figure S20. (a) Photoswitching of dyad **DTE-Cy3** in water under 405 nm and 630 nm irradiation and the corresponding change in (b) UV-vis absorption spectrum (25 °C) and (c) NMR spectra (D_2O, 500 MHz).

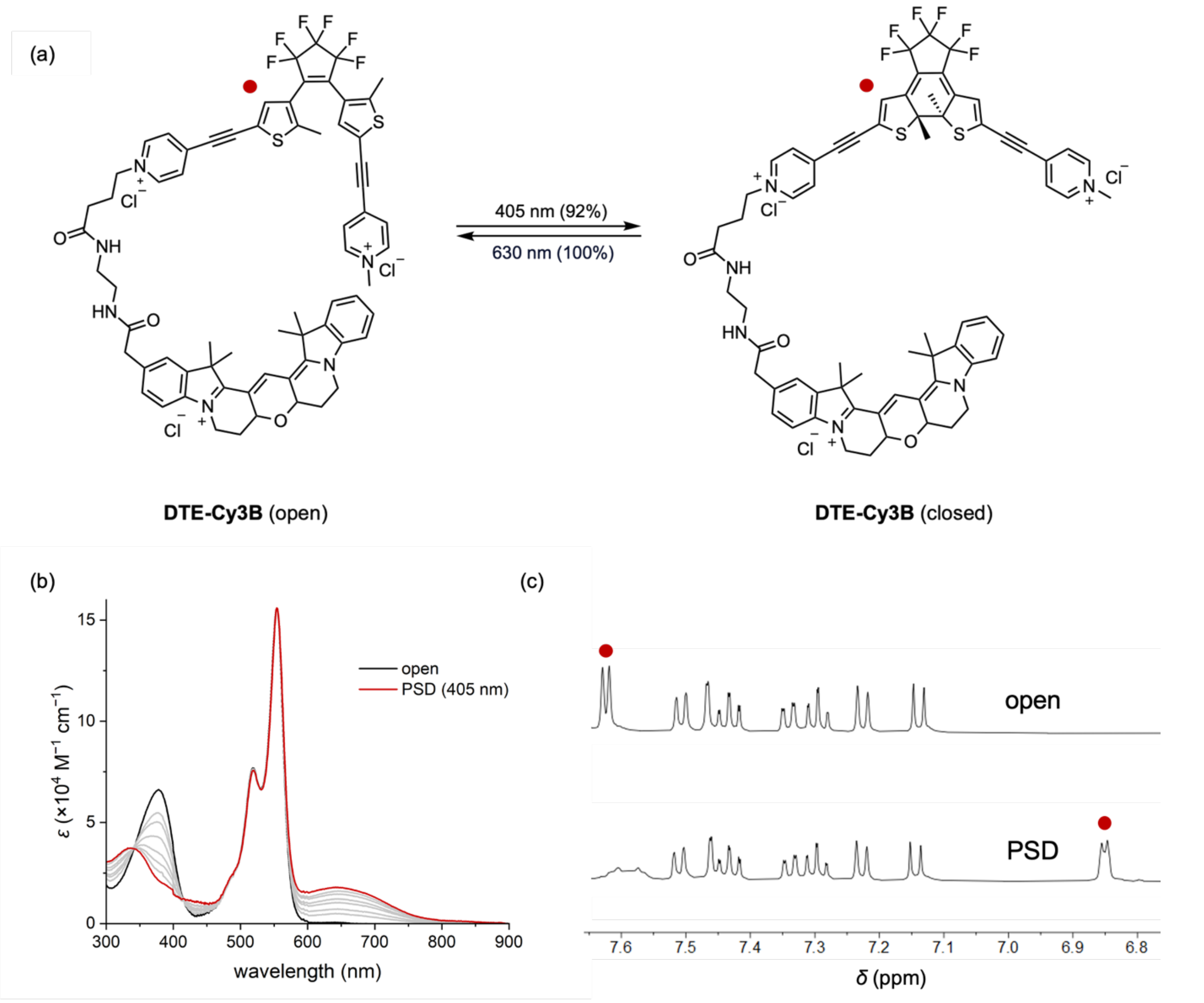

Figure S21. Photoswitching of dyad **DTE-Cy3B** in water under 405 nm and 630 nm irradiation and the corresponding change in (b) UV-vis absorption spectrum (25 °C) and (c) NMR spectra (D_2O, 500 MHz).

Table S7. PSD of the photoswitches in the dyad system.

	PSD_{n-q} (%)	PSD_{q-n} (%)
**DTE-Cy3**[c]	94	100 (630 nm)
**DTE-CyB**[c]	92	100 (630 nm)

[a]Measured in DMSO based on fluorescence quenching. [b]Measured in MeCN based on fluorescence quenching. [c]Measured in D_2O by both fluorescence quenching and ^{1}H NMR spectroscopy.

S12. Synthetic Procedures

DAZ

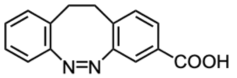

DAZ (52 mg, 0.20 mmol) was dissolved in THF (0.5 mL), then methanol (0.5 mL), water (0.5 mL) and lithium hydroxide (13 mg, 0.55 mmol) were added. The mixture was sonicated and the solution stirred for 3 hours at room temperature. The mixture was then acidified with 2 M HCl (0.3 mL), and the precipitate was collected via filtration, washed with water, and dried to give compound **DAZ-acid** (40 mg, 81%). Removal of the methyl ester was confirmed by proton NMR, and the compound was used in the next step without further purification. NMR δ_H (400 MHz, DMSO-d6) 13.09 (1H, s, COOH), 7.60 (1H, dd, J_{HH} 7.9 & 1.8, ArH), 7.31 (1H, d, J_{HH} 1.7, ArH), 7.27–7.15 (2H, m, ArH), 7.14–7.02 (2H, m, ArH), 6.89 (1H, dd, J_{HH} 7.7 & 1.2), 3.05–2.77 (4H, m, CH_2).

DAZ-RhoB

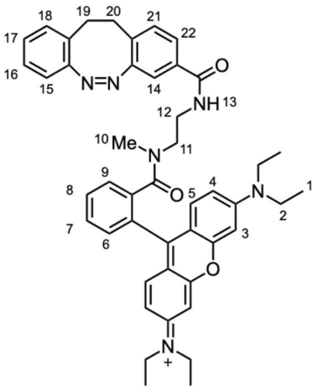

Rho-NHBoc (96 mg, 0.16 mmol) was stirred in a 1:9 TFA:dichloromethane mixture (2 mL) for 2 h at room temperature, and then the solvent was removed under reduced pressure. The crude deprotected dye was added to a pre-stirred solution of compound **DAZ-acid** (37 mg, 0.15 mmol), HBTU (83 mg, 0.22 mmol), and DIPEA (51 µL, 0.29 mmol) in DMF (3 mL). The reaction mixture was stirred at room temperature overnight, then ethyl acetate (50 mL) was added, and the organic layer washed with brine (6 × 30 mL). The solution was dried over anhydrous sodium sulfate, then the solvent removed under reduced pressure. The crude residue was purified by flash silica column chromatography (10% MeCN in dichloromethane) to give **DAZ-RhoB** (20 mg, 18%) as a purple solid. The NMR spectrum was complicated by the presence of rotamers around the amide bond. Some protons are split into two environments, with the *trans* as the major isomer (as drawn), and *cis* as the minor isomer (34:66 ratio). The major has been assigned in the proton NMR. All peaks observed in the carbon NMR are reported. NMR δ_H (600 MHz, DMSO) 8.28 (t, J_{HH} 5.7, H^{13}), 7.75 – 7.70 (2H, m, H^7, H^8), 7.67–7.65 (1H, m, H^9), 7.57–7.51 (1H, m, H^6), 7.35 (dd, J_{HH} 8.0 & 2.0, H^{17}), 7.23–7.04 (6H, m, H^4, H^5, H^{15}, H^{16}, H^{21}, H^{22}) 6.88–6.82 (2H, m, H^{18}, H^3), 3.64 (1H, m, H^2), 3.28–3.17 (1H, m, H^{11}), 2.96 (1H, s, H^{10}), 2.93–2.89 (1H, m, H^{12}), 2.88–2.80 (2H, m, H^{20} & H^{19}), 1.20 (1H, t, J_{HH} 7.1, H1); δ_C (151 MHz, DMSO) 167.8, 164.8, 157.0, 157.0, 155.6, 155.1, 155.0, 154.7, 136.1, 132.7, 132.5, 131.7, 131.4, 130.5, 130.3,

130.1, 129.9, 129.8, 129.7, 129.4, 129.2, 127.7, 127.3, 127.3, 126.9, 125.8, 125.5, 118.3, 117.2, 114.1, 112.9, 95.8, 49.5, 46.0, 45.3, 37.6, 37.3, 36.0, 32.0, 30.9, 30.6, 30.5, 12.4; HRMS $[C_{46}H_{49}N_6O_3]^+$ 733.3861, found 733.3877; IR (cm^{-1}) 2976, 1589, 1413, 1337, 1275, 1247, 1181, 1133, 1075, 841, 757, 684.

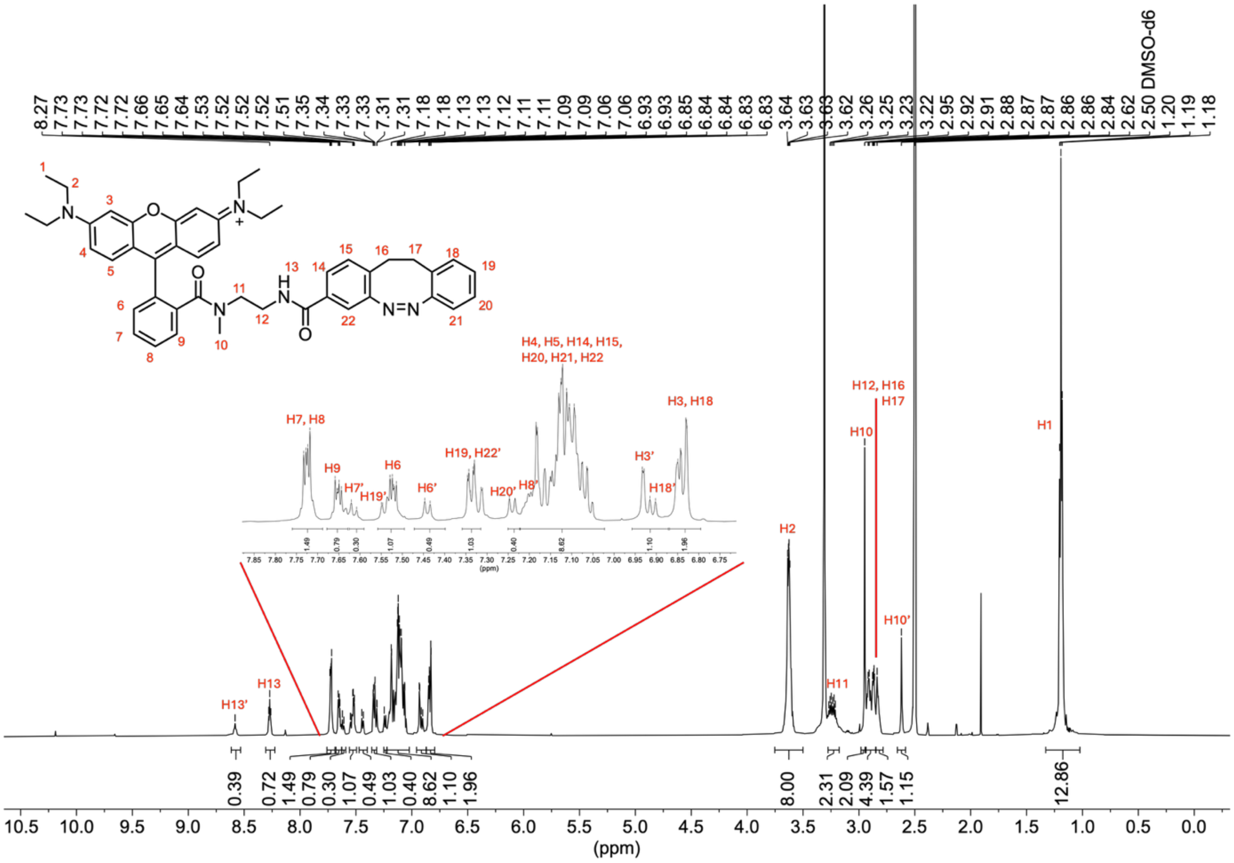

Figure S22. ^{1}H NMR spectrum of **DAZ-RhoB** (600 MHz, DMSO-d_6).

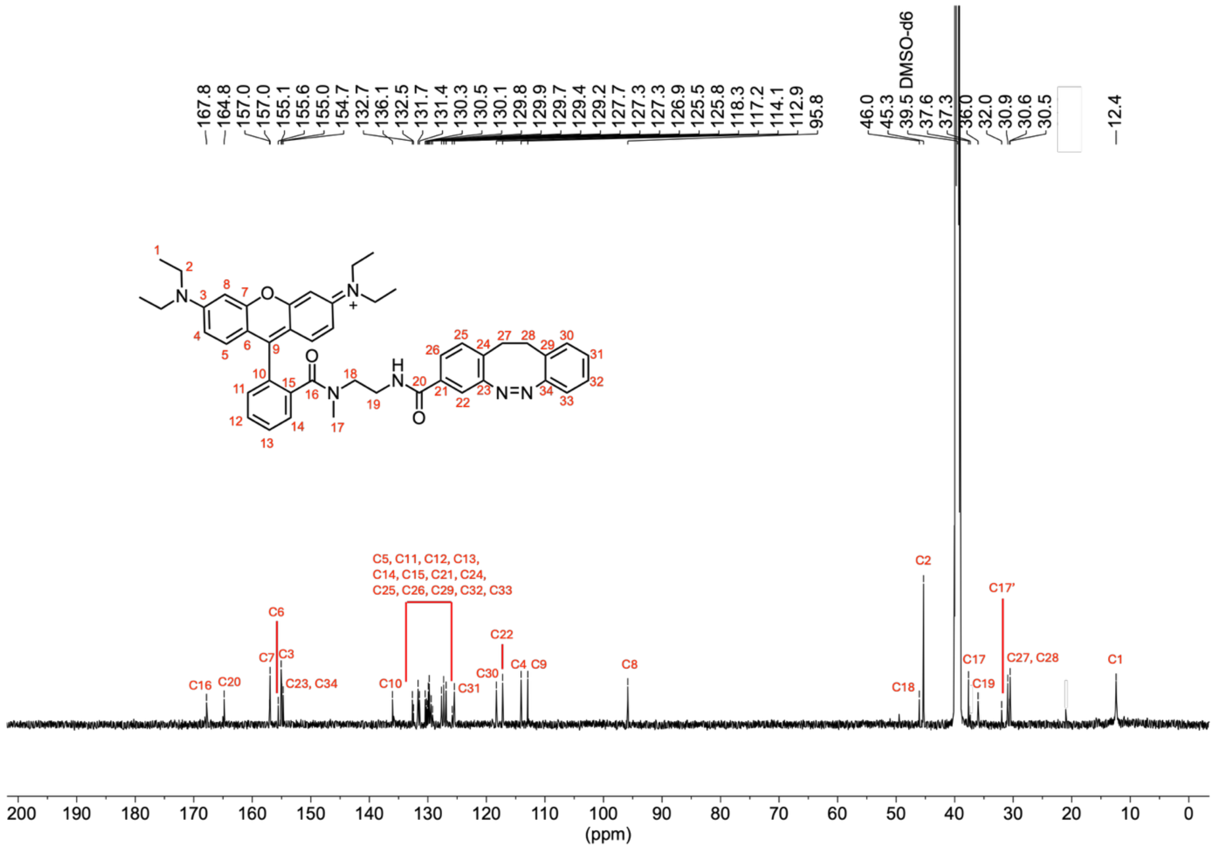

Figure S23. ^{13}C NMR spectrum of **DAZ-RhoB** (600 MHz, DMSO-d_6).

Diethyl isopropylidene succinate (2)

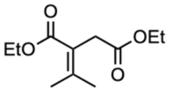

Potassium *tert*-butoxide (12.9 g, 0.115 mol) was dissolved in *tert*-butanol (115 mL), and diethyl succinate (19.1 mL, 20.0 g, 0.115 mol) was added. The solution was stirred at room temperature for 30 min, before acetone (12.8 mL, 10.0 g, 0.172 mol) was added. The solution immediately changed from pale yellow to orange. The solution was heated at reflux overnight. The reaction was cooled to room temperature and aqueous HCl was added (100 mL, 2 M). The solution was extracted with diethyl ether (3 × 100 mL), and the organic layers were combined, dried over $MgSO_4$, filtered and concentrated to give a brown oil. This oil was dissolved in ethanol (300 mL) and concentrated sulfuric acid (8 mL) was added. The solution was heated at reflux overnight. After cooling to room temperature, saturated aqueous sodium bicarbonate solution (300 mL) was added. The organic solvents were removed under reduced pressure, and the aqueous solution was extracted with diethyl ether (3 × 100 mL). The organic layers were

combined, washed with brine, dried over $MgSO_4$, filtered and concentrated to give a brown oil. Vacuum distillation (0.2 mbar, 35–40 °C) yielded the product as a pale-yellow oil (7.45 g, 30%) with the other major fractions being starting material which can be recycled, and a mixture of product and starting material which can be redistilled. NMR δ_H (400 MHz, $CDCl_3$): 4.18 (4H, q, $^3J_{HH}$ 7.1, $–OCH_2CH_3$), 4.13 (4H, q, $^3J_{HH}$ 7.1, $–OCH_2CH_3$), 3.36 (2H, s, $–CH_2–$), 2.14 (3H, s, $–CH_3$), 1.86 (3H, s, $–CH_3$), 1.27 (3H, t, $^3J_{HH}$ 7.1, $–OCH_2CH_3$), 1.24 (3H, t, $^3J_{HH}$ 7.1, $–OCH_2CH_3$).

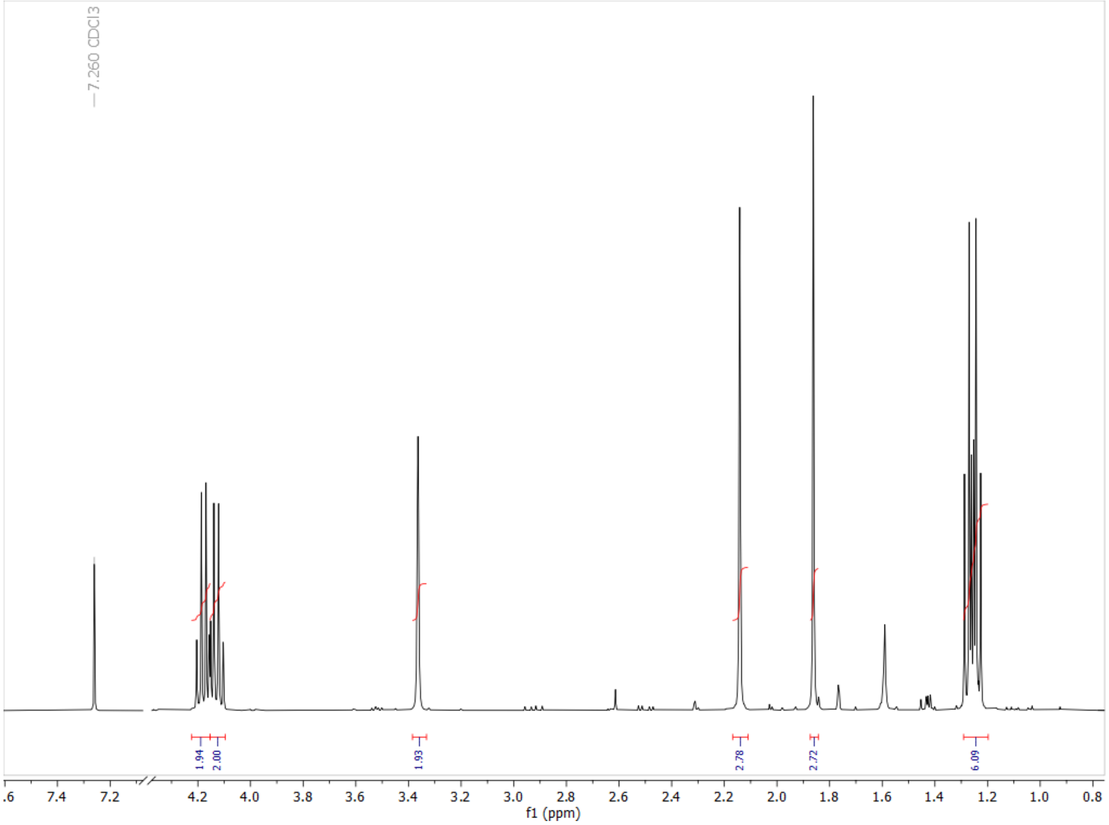

Figure S24. ^{1}H NMR spectrum of **2** (400 MHz, $CDCl_3$).

2-Methyl-1-(2-methyl-1H-indol-3-yl)propan-1-one (4)

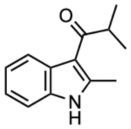

2-Methyl-1*H*-indole (5.0 g, 38 mmol) was dissolved in *N,N*-dimethylisobutyramide (6.4 mL, 50 mmol) and cooled in an ice bath. Phosphorus oxychloride (4.4 mL, 48 mmol) was added slowly. The cooling bath was removed, and the reaction mixture was stirred at 120 °C for 2 h, at which point

TLC showed complete consumption of starting material. The reaction mixture was cooled and added to water (100 mL) and dichloromethane (150 mL). The layers were separated, and the organic layer was washed with water (100 mL). The aqueous layers were combined and washed with diethyl ether (1 × 100 mL). Aqueous sodium hydroxide solution (20% w/w) was added to the aqueous layer, giving a bright yellow precipitate. Ethyl acetate (100 mL) was added to dissolve the precipitate and the layers were separated. The aqueous layer was extracted with further ethyl acetate (3 × 100 mL). The ethyl acetate extracts were combined, dried over $MgSO_4$, filtered and concentrated, to give an orange solid, which was recrystallized from ethanol to give the desired product as a yellow crystalline solid (5.3 g, 84%). TLC R_f: 0.81 (SiO_2, 1:1 petrol:ethyl acetate); NMR δ_H (400 MHz, $CDCl_3$): 8.98 (1H, br s, NH), 7.97 (1H, d, $^3J_{HH}$ 8.0, ArH), 7.36 –7.37 (1H, m, ArH), 7.27–7.18 (2H, m, ArH), 3.53 (1 H, sept, $^3J_{HH}$ 6.8, –C*H*(CH_3)$_2$), 2.77 (3H, s, –CH_3), 1.29 (6H, d, $^3J_{HH}$ 6.8, –CH(C*H*$_3$)$_2$); δ_C (101 MHz, $CDCl_3$): 203.4, 144.6, 131.1, 127.2, 122.3, 122.1, 120.1, 113.4, 111.2, 37.6, 19.0, 15.8; MS (ESI$^+$) *m/z* = 202.2 [M+H]$^+$, 224.2 [M+Na]$^+$.

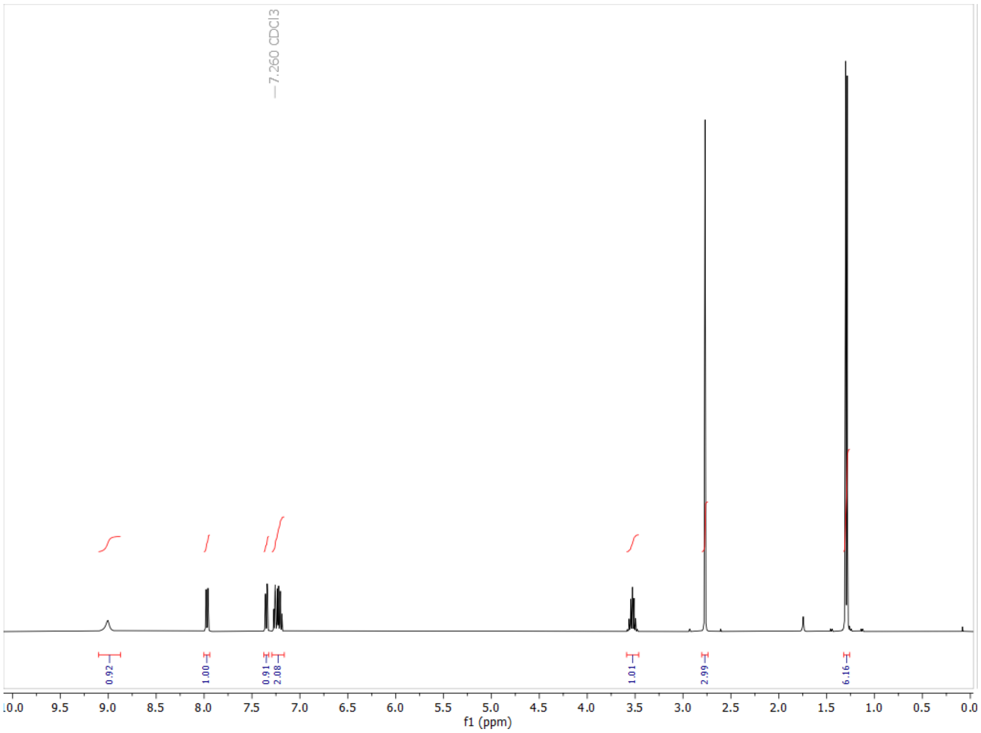

Figure S25. ^{1}H NMR spectrum of **4** (400 MHz, $CDCl_3$).

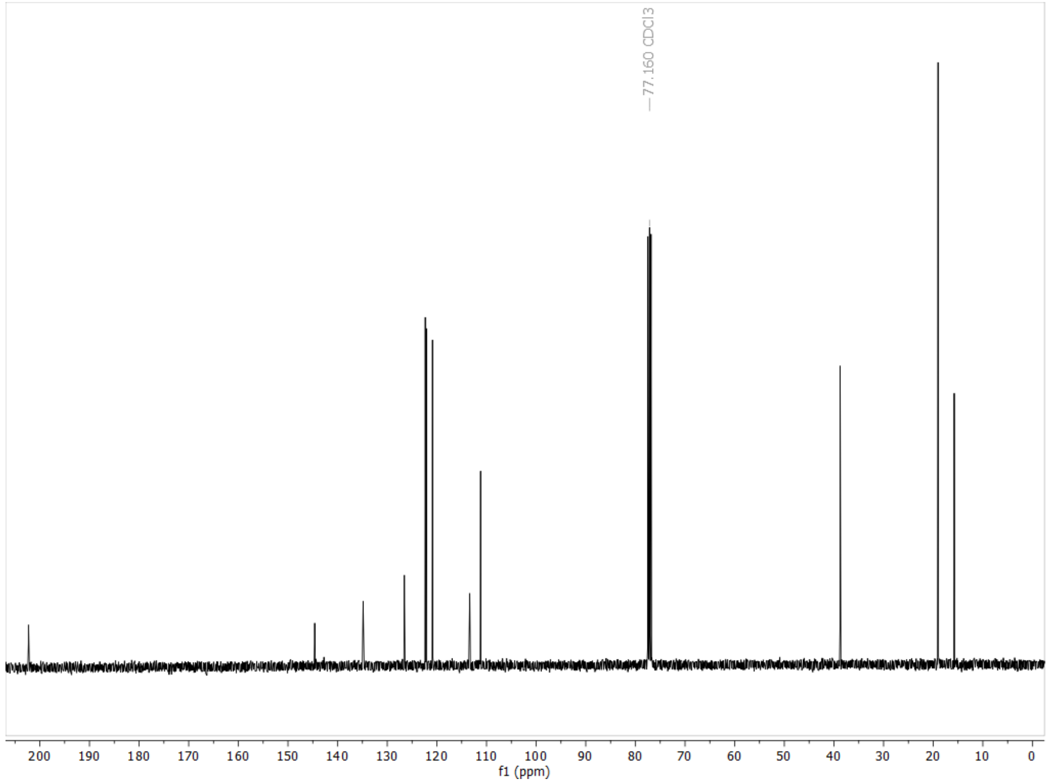

Figure S26. ^{13}C NMR spectrum of **4** (101 MHz, $CDCl_3$).

2-Methyl-1-(2-methyl-1-tosyl-1*H*-indol-3-yl)propan-1-one (5)

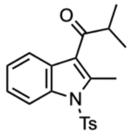

4 (2.70 g, 13.4 mmol), tosyl chloride (5.11 g, 26.8 mmol) and potassium hydroxide (1.51 g, 26.8 mmol) were stirred in dichloromethane (100 mL) at room temperature for 24 h. Water (100 mL) was added and the layers separated. The aqueous layer was extracted with dichloromethane (3 × 100 mL). The organic layers were combined, and washed with brine (100 mL), dried over $MgSO_4$, filtered, and concentrated *in vacuo*. The crude yellow oil was purified by silica gel column chromatography (petroleum ether to 10% ethyl acetate in petroleum ether), to give the product as a white solid (3.75 g, 89%). TLC R_f: 0.36 (SiO_2, 9:1 petrol:ethyl acetate); NMR δ_H (400 MHz, $CDCl_3$) 8.28 (1H, dd, $^3J_{HH}$ 7.6, $^4J_{HH}$ 1.6, ArH_{indole}), 7.73–7.68 (3H, m, ArH_{indole} and ArH_{tosyl} overlapping), 7.36–7.28 (2H, m, ArH_{indole}), 7.24 (2 H, d, $^3J_{HH}$ 8.3, ArH_{tosyl}), 3.35 (1H, sept, $^3J_{HH}$ 6.8, –C*H*(CH_3)$_2$), 2.80 (3H, s, indole CH_3), 2.36 (3H, s, tosyl CH_3), 1.18

(6H, d, $^3J_{HH}$ 6.8, –CH(CH_3)$_2$); δ_C (101 MHz, $CDCl_3$): 199.1, 145.0, 140.2, 136.6, 134.5, 131.0, 127.0, 126.7, 124.7, 124.3, 120.7, 120.3, 115.5, 40.0, 21.7, 18.7, 14.2.

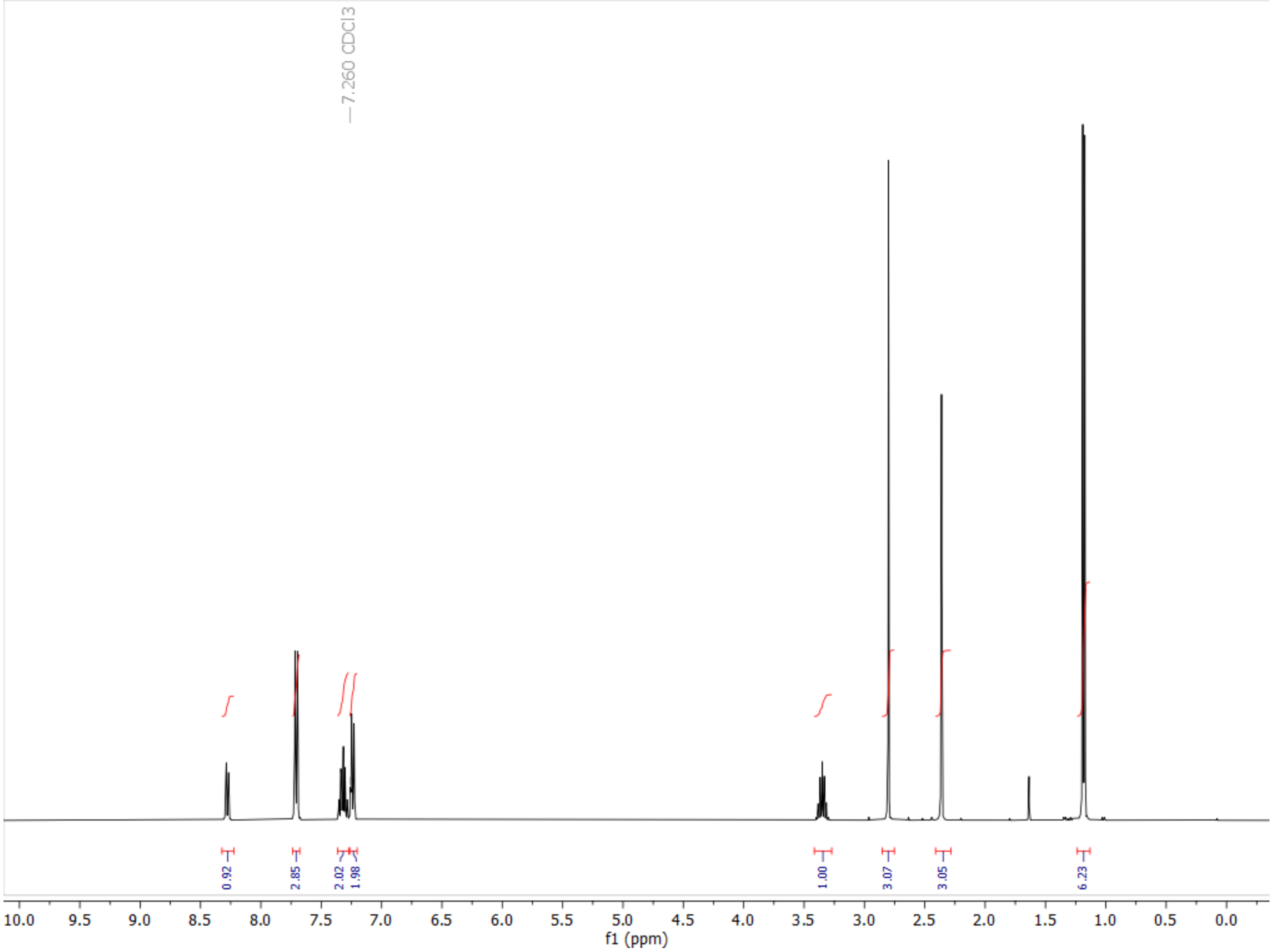

Figure S27. ^{1}H NMR spectrum of **5** (400 MHz, $CDCl_3$).

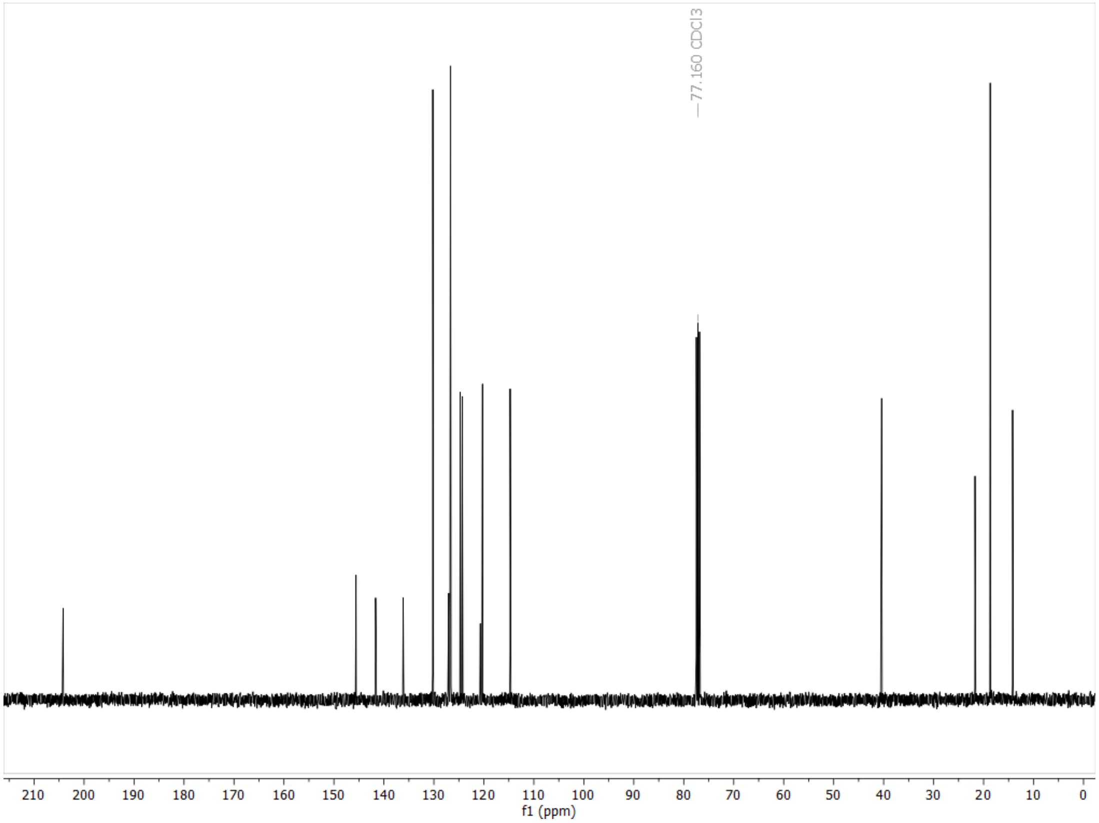

Figure S28. ^{13}C NMR spectrum of **5** (101 MHz, $CDCl_3$).

(*E*)-3-(Ethoxycarbonyl)-5-methyl-4-(2-methyl-1-tosyl-1*H*-indol-3-yl)-2-(propan-2-ylidene)hex-3-enoic acid (6)

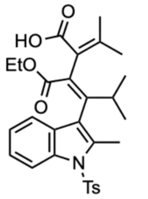

Diisopropylamine (3.95 mL, 28.0 mmol) was dissolved in anhydrous tetrahydrofuran (15 mL) and cooled to –78 °C. *n*-BuLi (17.5 mL of a 1.6 M solution in hexanes, 28.0 mmol) was added and the solution stirred for 10 min at –78 °C. **2** (5.45 g, 25.4 mmol) was added as a solution in anhydrous tetrahydrofuran (10 mL). The resulting solution was stirred at –78 °C for 30 min, at which point **5** (6.02 g, 16.9 mmol) was added as a solution in anhydrous tetrahydrofuran (10 mL). The cooling bath was removed and once the reaction had reached room temperature, it was further heated at reflux for 3 days. After cooling to room temperature, aqueous HCl (300 mL, 2 M) was added, and the solution extracted with diethyl ether (3 × 300 mL). The organic layers were combined, washed with brine, dried over $MgSO_4$, filtered and concentrated. The crude mixture was purified by silica gel column

chromatography (80:20 petroleum ether:ethyl acetate) and the product recrystallized from acetonitrile to give **6** as a white solid (2.88 g, 29%). TLC R_f: 0.13 (SiO_2, 8:2 petrol:ethyl acetate); NMR δ_H (400 MHz, $CDCl_3$) 8.03 (1H, d, $^3J_{HH}$ 8.0, ArH_{indole}), 7.57 (2H, d, $^3J_{HH}$ 8.4, ArH_{tosyl}), 7.24 (1H, d, $^3J_{HH}$ 8.0, ArH_{indole}), 7.19 (2H, d, $^3J_{HH}$ 8.4, ArH_{tosyl}), 7.16–7.14 (1H, m, ArH_{indole}), 7.10–7.06 (1H, m, ArH_{indole}), 4.35 (2H, q, $^3J_{HH}$ 7.2, $–OCH_2CH_3$), 3.55 (1H, sept, $^3J_{HH}$ 6.8, $–CH(CH_3)_2$), 2.37 (3H, s, tosyl CH_3), 2.32 (3H, s, indole CH_3), 1.74 (3H, s, $=C(CH_3)_2$), 1.63 (3H, s, $=C(CH_3)_2$), 1.36 (3H, t, $^3J_{HH}$ 7.2, $–OCH_2CH_3$), 1.04 (3H, d, $^3J_{HH}$ 6.8, $–CH(CH_3)_2$), 0.95 (3H, d, $^3J_{HH}$ 6.8, $–CH(CH_3)_2$); δ_C (101 MHz, $CDCl_3$) 169.5, 168.6, 151.3, 144.6, 136.5, 136.4, 134.3, 131.1, 130.0, 129.8, 126.5, 124.4, 124.0, 122.9, 121.0, 118.1, 114.4, 61.7, 33.7, 24.5, 22.6, 21.9, 21.6, 21.2, 15.0, 14.4; MS (ESI$^-$) m/z = 522.1 $[M–H]^-$.

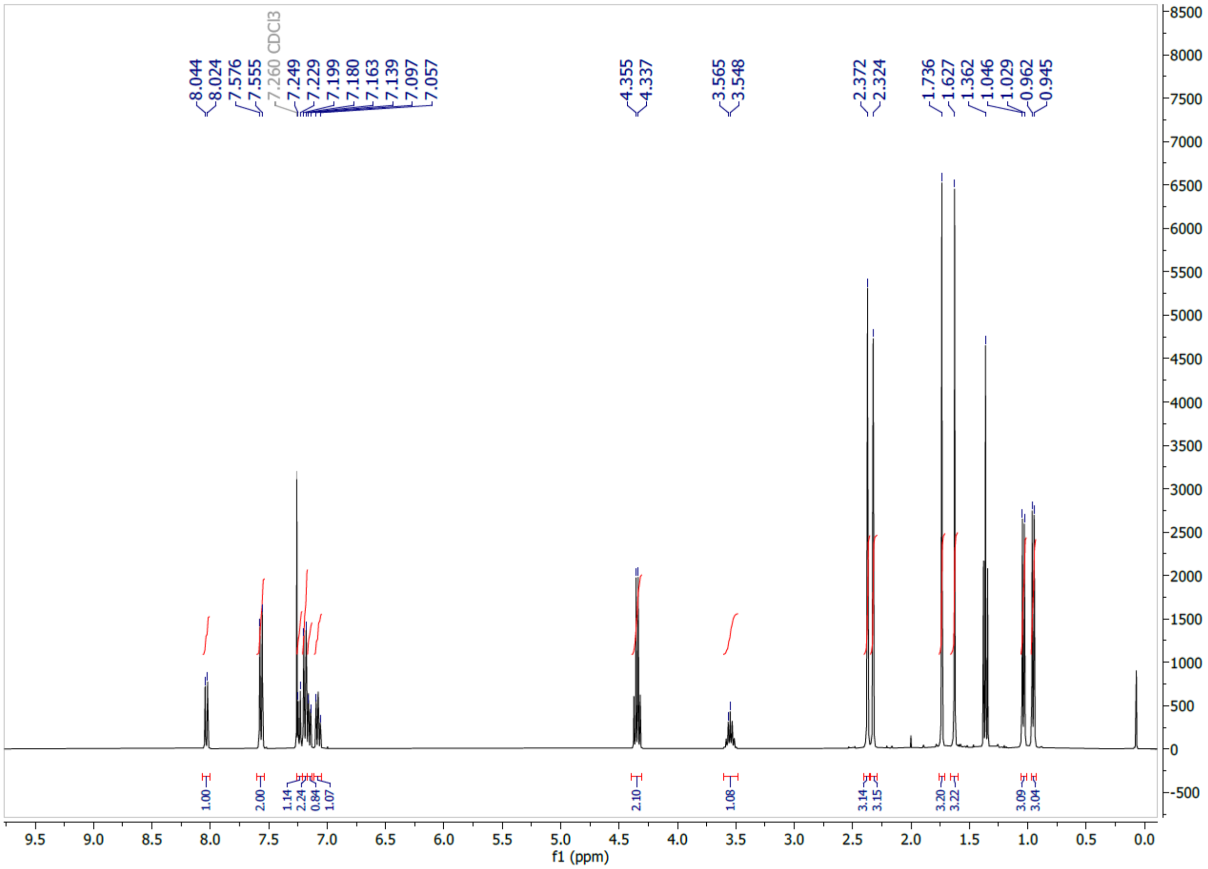

Figure S29. ^1H NMR spectrum of **6** (400 MHz, $CDCl_3$).

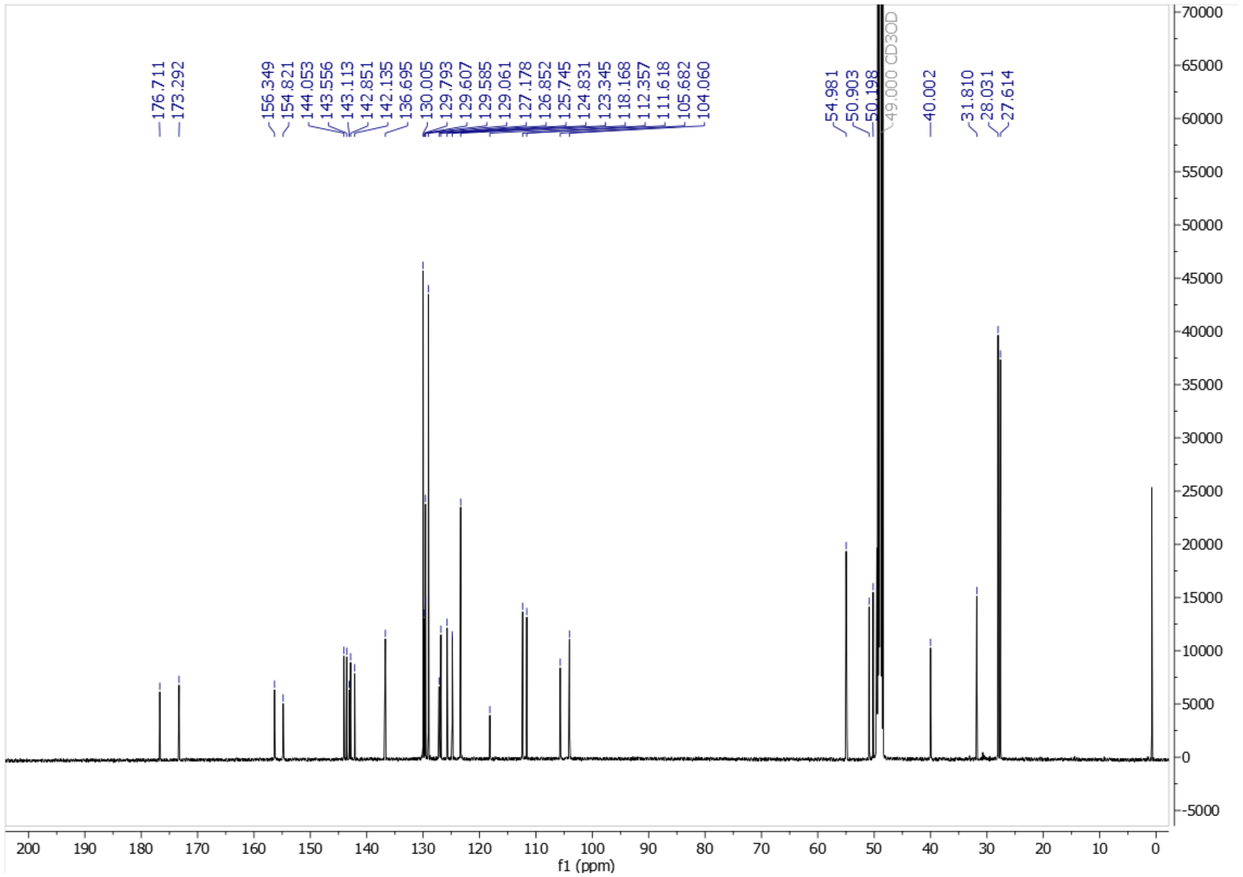

Figure S30. ^{13}C NMR spectrum of **6** (101 MHz, $CDCl_3$).

(*E*)-2-(2-Methyl-1-(2-methyl-1*H*-indol-3-yl)propylidene)-3-(propan-2-ylidene)succinic acid (8)

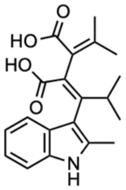

Example procedure: Half ester **6** (2.88 g, 5.50 mmol) was dissolved in ethanol (100 mL) and potassium hydroxide (1.85 g, 33.0 mmol) was added. The reaction was heated at reflux for 2 days, at which point water (5 mL) was added, and the reaction was heated at reflux for a further 2 days. The reaction was cooled to room temperature and poured into ice cold aqueous HCl (200 mL, 2 M). The solution was saturated with solid sodium chloride and extracted with ethyl acetate (3 × 200 mL). The organic layers were combined, washed with brine, dried over Na_2SO_4, filtered and concentrated to give the crude product as a brown solid (1.88 g, quant.) which was used without further purification. MS (ESI$^-$) *m/z* = 340.1 [M–H]$^-$.

Fulgide (10)

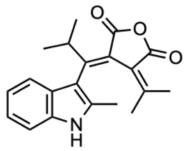

Diacid **8** (1.88 g, 5.51 mmol) and *N,N'*-dicyclohexylcarbodiimide (3.41 g, 16.5 mmol) were dissolved in anhydrous dichloromethane (30 mL) and stirred at room temperature under argon for 1 h, after which time no diacid starting material was present. The reaction mixture was filtered through Celite and concentrated under reduced pressure. The product was purified by silica gel column chromatography (neat dichloromethane) to give the fulgide **10** as a yellow-brown solid which darkens on standing in ambient light (597 mg, 79%). TLC R_f: 0.44 (SiO_2, 8:2 PE:EtOAc); NMR (open form) δ_H (400 MHz, $CDCl_3$): 8.01 (1H, br s, NH), 7.44 (1H, d, $^3J_{HH}$ 8.0, ArH$_{indole}$), 7.26 (1H, d, $^3J_{HH}$ 8.0, ArH$_{indole}$), 7.19–7.11 (2H, m, ArH$_{indole}$), 4.11 (1H, sept., $^3J_{HH}$ 6.8, –C*H*(CH_3)$_2$), 2.16 (3H, s, =C(C*H*$_3$)$_2$), 2.13 (3H, s, =C(C*H*$_3$)$_2$), 1.42 (3H, d, $^3J_{HH}$ 6.8, –CH(C*H*$_3$)$_2$), 1.02 (3H, s, indole CH_3), 1.01 (3H, d, $^3J_{HH}$ 6.8, –CH(C*H*$_3$)$_2$); HPLC R_t = 18.5 min; UV/Vis (CH_2Cl_2): λ_{max} (open) = 381 nm, λ_{max} (closed) = 534 nm.

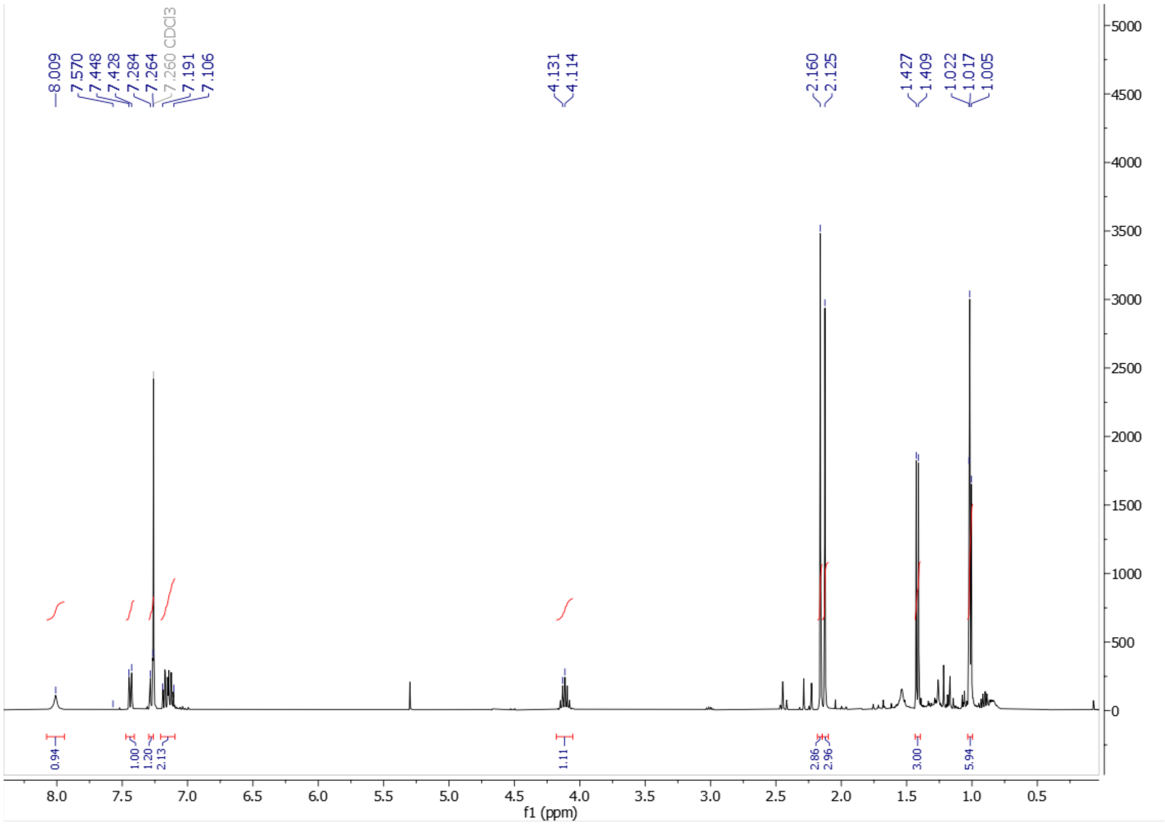

Figure S31. ^{1}H NMR spectrum of **10** (400 MHz, $CDCl_3$).

3-((2-(*Tert*-butoxy)-2-oxoethyl)carbamoyl)-4-isopropyl-1,1,9a-trimethyl-9,9a-dihydro-1*H*-carbazole-2-carboxylic acid (11, closed form)

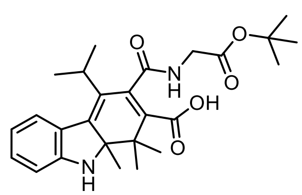

Open fulgide **10** (600 mg, 1.86 mmol) was dissolved in anhydrous acetonitrile (15 mL). The solution was irradiated with a 405 nm LED with stirring until no open form **1o** was visible by TLC. *Tert*-butyl glycinate hydrochloride (933 mg, 5.57 mmol) and *N*,*N*-diisopropylethylamine (1.94 mL, 11.1 mmol) were added and the reaction mixture stirred overnight at room temperature. All solvents were removed at room temperature, and the crude material was used in the next step without further purification. Attempts to work up resulted in significant amounts of starting material being recovered despite having been consumed in the initial reaction.

FULG (closed form)

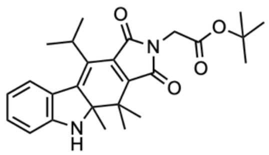

Mixed acid/amide **11** (674 mg, 1.48 mmol, known to contain unreacted fulgide **10**) was dissolved in toluene (50 mL) and acetic anhydride (12.1 g, 11.2 mL, 119 mmol) was added. The reaction was stirred at room temperature overnight after which time **11** had been consumed (monitored by TLC). The reaction mixture was washed with water (50 mL). The aqueous layer was extracted with toluene (3 × 50 mL). The organic layers were combined, washed with brine, dried over Na_2SO_4, filtered and concentrated. The crude material was roughly separated by silica gel column chromatography (1:1 petrol ether:CH_2Cl_2 to neat CH_2Cl_2). The fraction containing product was concentrated and redissolved in acetonitrile (10 mL), then shaken with 1 M aq. NaOH (10 mL). The aqueous layer was extracted with dichloromethane (3 × 20 mL). All organic layers were combined and concentrated. The resulting residue was passed through a plug of silica with dichloromethane as the eluent. The purple fraction was concentrated and the residue triturated with pentane. The product **13** was collected as a red solid (35 mg, 5%). NMR (closed form) δ_H (600 MHz, CD_3CN): 7.56 (1H, d, $^3J_{HH}$ 8.4, ArH), 7.15 (1H, dd, $^3J_{HH}$ 7.6, $^4J_{HH}$ 1.2, ArH), 6.75–6.71 (2H, m, 2 ×

ArH), 4.58 (1H, br s, NH), 4.08 (2H, s, CH_2), 3.55 (1H, sept., $^3J_{HH}$ 7.2, ^{i}Pr CH), 1.48 (3H, s, CH_3), 1.44 (3H, d, $^3J_{HH}$ 6.7, ^{i}Pr CH_3), 1.42 (9H, s, ^{t}Bu CH_3), 1.14 (6H, overlapping singlet and doublet, ^{i}Pr CH_3 and CH_3); δ_C (151 MHz, CD_3CN): 170.4, 169.9, 168.1, 157.5, 149.6, 142.8, 140.4, 132.2, 127.6, 127.4, 124.4, 119.5, 112.1, 82.9, 71.2, 40.4, 38.5, 28.6, 28.1, 20.5, 20.4, 20.0, 19.9, 19.2; UV/Vis (MeCN): λ_{max} (ε) = 523 nm (4140 mol^{-1} dm^3 cm^{-1}).

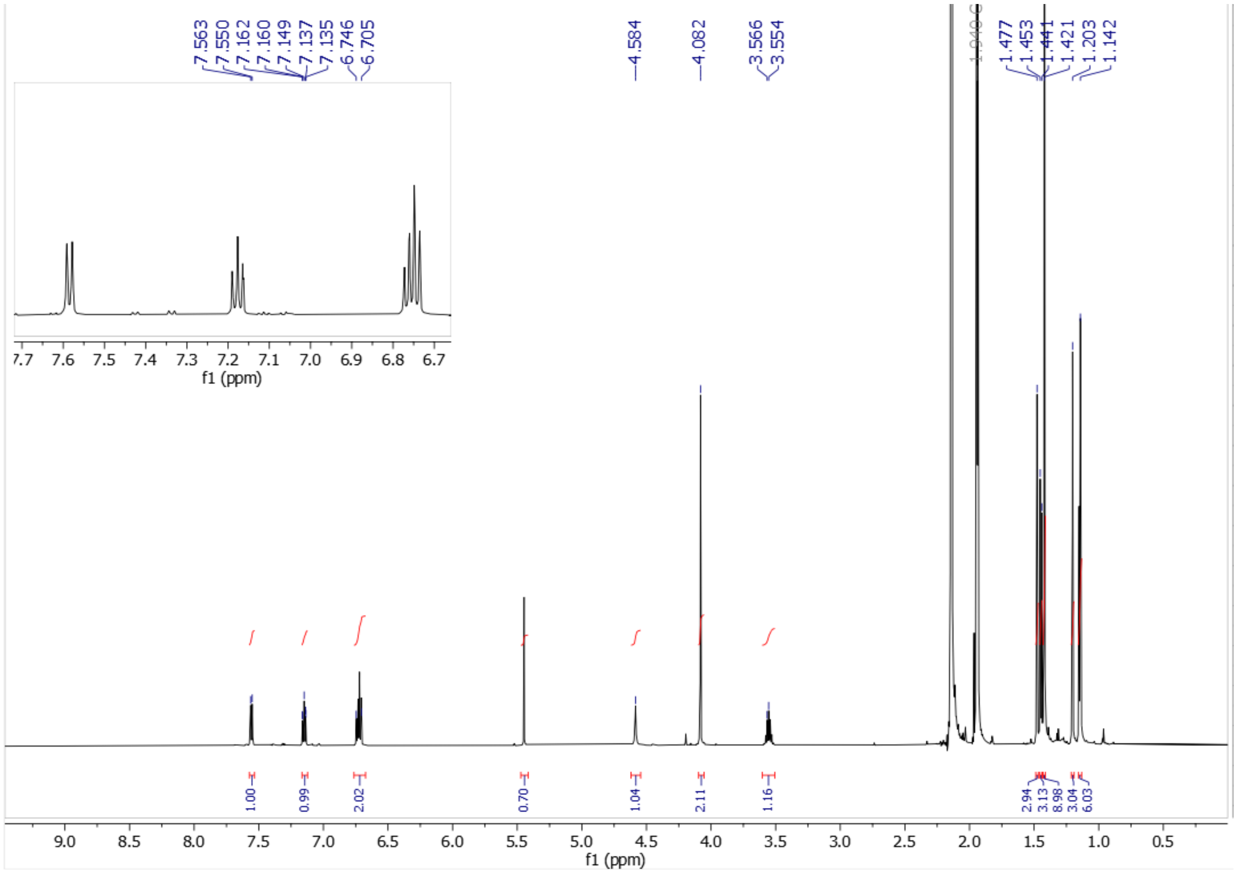

Figure S32. ^{1}H NMR spectrum of **FULG** in the closed form (400 MHz, CD_3CN).

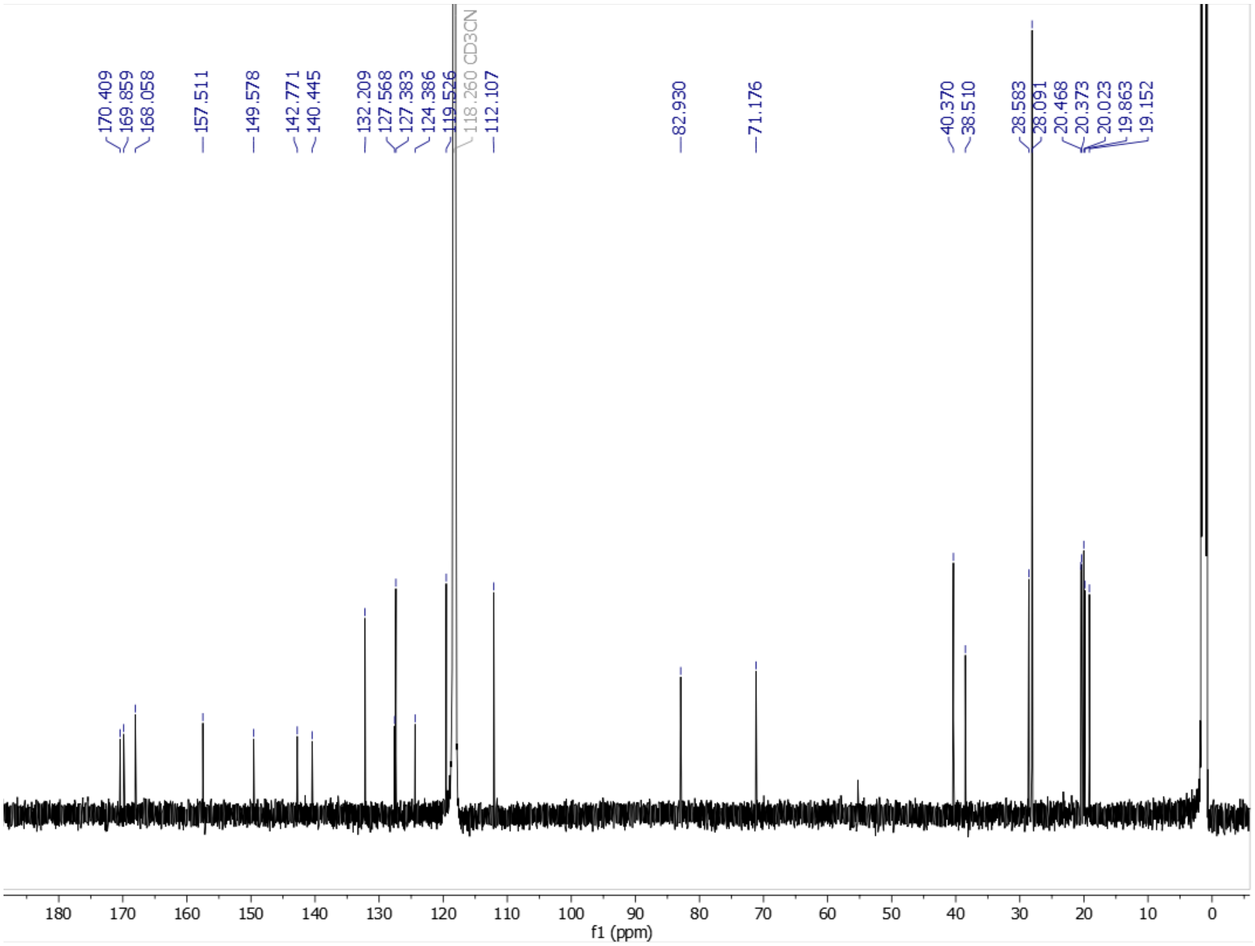

Figure S33. ^{13}C NMR spectrum of **FULG** in the closed form (151 MHz, CD_3CN).

NMR (open form) δ_H (600 MHz, CD_3CN): 9.30 (1H, br s, NH), 7.40 (1H, d, $^3J_{HH}$ 7.8, ArH), 7.31 (1H, d, $^3J_{HH}$ 7.8, ArH), 7.09 (1H, td, $^3J_{HH}$ 7.8, $^4J_{HH}$ 1.2, ArH), 7.03 (1H, td, $^3J_{HH}$ 7.8, $^4J_{HH}$ 1.2, ArH), 4.23 (1H, sept., $^3J_{HH}$ 7.2, ^{i}Pr CH), 4.19 (2H, s, CH_2), 2.11 (3H, s, CH_3), 2.03 (3H, s, CH_3), 1.45 (9H, s, ^{t}Bu), 1.32 (3H, d, $^3J_{HH}$ 7.2 ^{i}Pr CH_3), 0.96 (6H, overlapping singlet and doublet, ^{i}Pr CH_3 and CH_3); δ_C (151 MHz, CD_3CN): 168.6, 168.2, 167.9, 156.1, 149.5, 136.7, 134.1, 128.0, 126.3, 124.7, 122.0, 120.8, 120.6, 114.0, 111.6, 82.9, 40.4, 33.2, 28.1, 26.7, 23.6, 21.7, 21.3, 13.1; λ_{max} (ε) = 356 nm (3740 mol^{-1} dm^3 cm^{-1}).

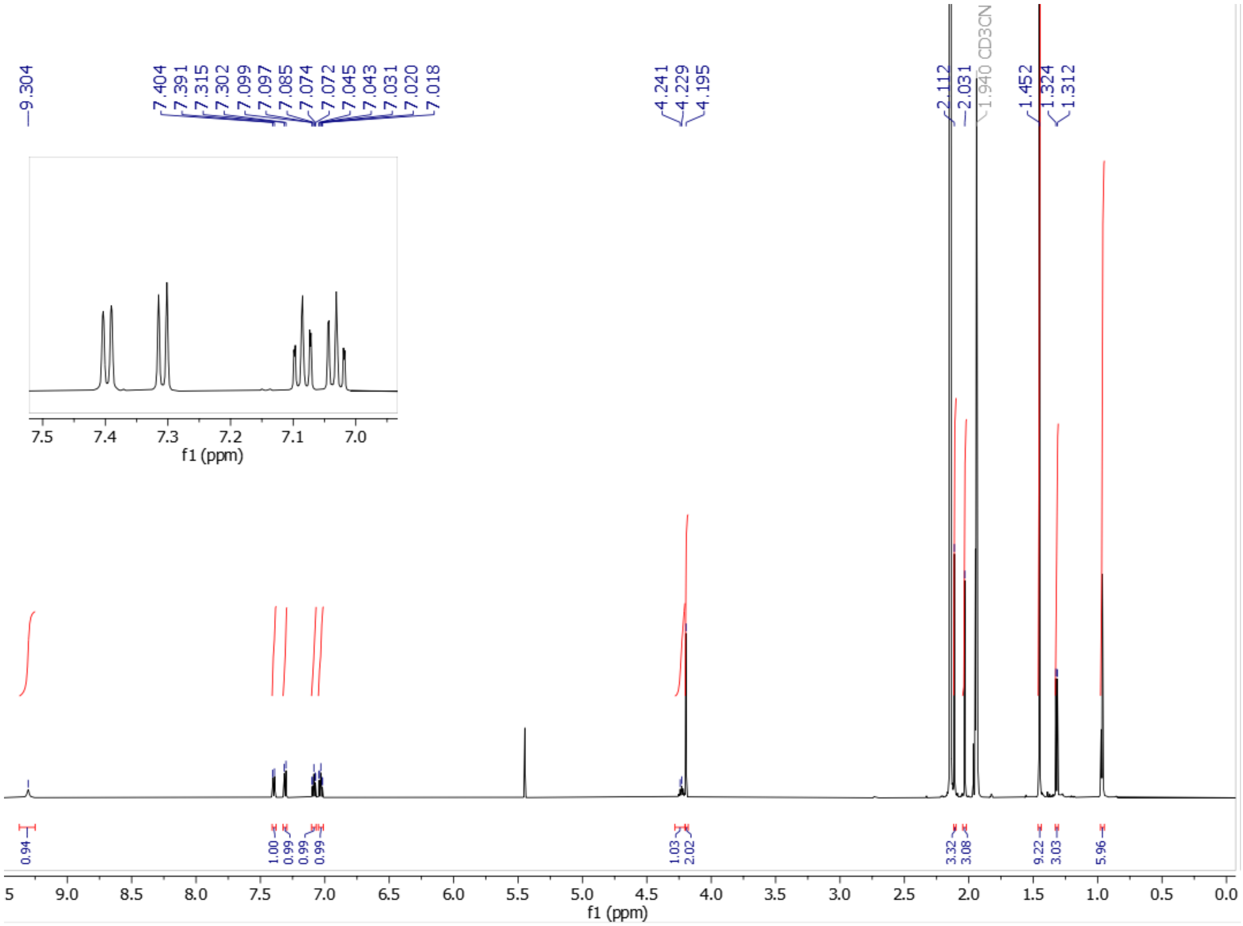

Figure S34. ^{1}H NMR spectrum of **FULG** in the open form (600 MHz, CD_3CN).

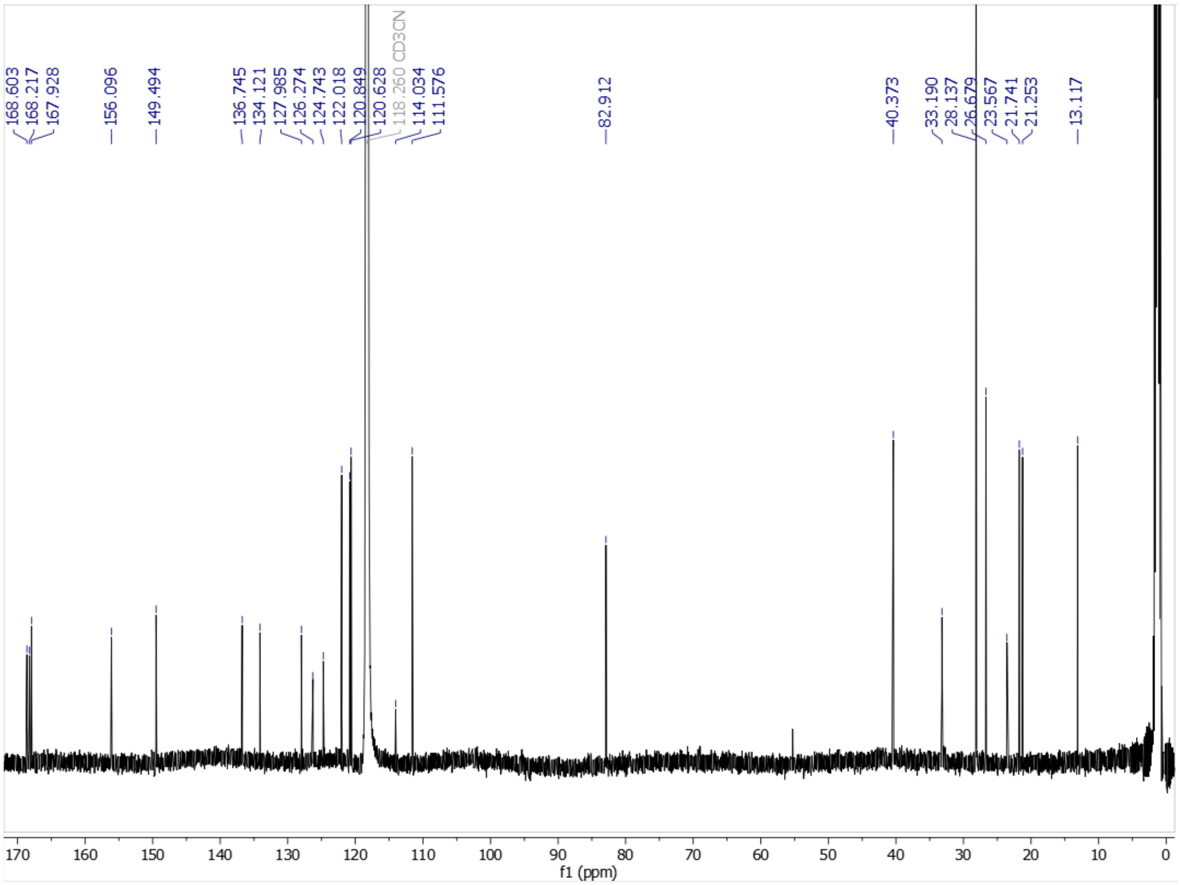

Figure S35. ^{13}C NMR spectrum of **FULG** in the open form (151 MHz, CD_3CN).

Fulgimide acid (FULG-acid, closed form)

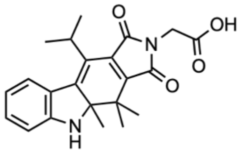

Fulgimide ^{t}Bu ester **FULG** (22 mg, 51 µmol) was dissolved in anhydrous dichloromethane (0.9 mL) and trifluoroacetic acid (100 µL) and triethylsilane (20.5 µL, 14.9 mg, 128 µmol) were added. The solution was stirred in the dark for 2 days, after which time TLC showed the starting material had been consumed. The solvent was removed under high vacuum and the residue was redissolved in dichloromethane (1 mL). This drying was repeated six times, after which the yellow brown solution returned to purple. The crude material was used without further purification. NMR δ_H (400 MHz, CD_3CN): 7.57 (1H, d, $^3J_{HH}$ 8.4, ArH), 7.50–7.44 (2H, m, 2 × ArH), 7.28 (1H, t, $^3J_{HH}$ ArH), 4.37 (1H, br s, NH), 4.24 (2H, s, CH_2), 2.50 (3H, s, CH_3), 1.26 (3H, s, CH_3), 1.10 (3H, d, $^3J_{HH}$ 6.7, ^{i}Pr CH_3), 0.99 (3H, d, $^3J_{HH}$ 6.7, ^{i}Pr CH_3), 0.90 (3H, s, CH_3), isopropyl CH is not visible.

FULG-RhoX

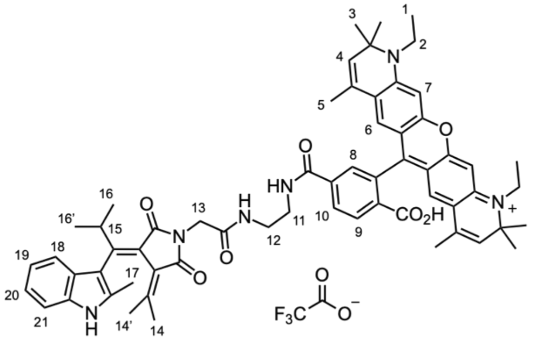

6'-Atto590-*N*-Boc-protected linker (2020 chem sci) (39 mg, 51 µmol) was dissolved in anhydrous dichloromethane (0.9 mL) and trifluoroacetic acid (0.1 mL) was added. The reaction was stirred at room temperature under argon until all starting material had been consumed (by TLC, 9:1 CH_2Cl_2:CH_3OH). The solvent was removed under high vacuum and the residue redissolved in dichloromethane (1 mL). The evaporation of solvent and redissolving was repeated five times, and the blue residue was then dried under vacuum.

Fulgimide acid **FULG-acid** (22 mg, 58 µmol) was dissolved in anhydrous DMF (1 mL). Diisopropylethylamine (180 µL, 1.04 mmol) and HBTU (22 mg, 58 µmol) were added, and the mixture was stirred at room temperature under argon for 1 h. The blue residue (see above) was dissolved in the minimum amount of DMF and transferred into the reaction, along with further DIPEA (200 µL). The reaction was stirred at room temperature for a further 30 h, at which time methanol (2 mL) was added and the reaction was left to stir for 30 min. The solvents were removed under reduced pressure and the residue was dissolved in dichloromethane (10 mL), washed with 2% aq. HCl (2 × 10 mL) and brine (10 mL). The organic layer was concentrated under reduced pressure and the crude product was subjected to column chromatography (SiO_2, CH_2Cl_2 to 8:2 CH_2Cl_2:CH_3OH). The fractions containing product were further purified by semi-preparative HPLC (water/methanol + TFA) to yield the product as a blue solid (5.3 mg, 9%). NMR δ_H (600 MHz, CD_3OD): 8.36 (1H, d, $^3J_{HH}$ 8.0, H^9), 8.21 (1H, dd, $^3J_{HH}$ 8.0, $^4J_{HH}$ 1.6, H^{10}), 7.88 (1H, d, $^4J_{HH}$ 1.6, H^8), 7.36 (1H, d, $^3J_{HH}$ 7.2, $H^{18/21}$), 7.25 (1H, d, $^3J_{HH}$ 7.2, $H^{18/21}$), 7.05 (1H, dt, $^3J_{HH}$ 7.2, $^4J_{HH}$ 1.2, $H^{19/20}$), 6.99 (1H, dt, $^3J_{HH}$ 7.2, $^4J_{HH}$ 1.2, $H^{19/20}$), 6.84 (2H, s, H^6), 6.75 (2H, s, H^7), 5.62 (2H, s, H^4), 4.25 (2H, s, H^{13}), 4.20 (1H, sept., $^3J_{HH}$ 7.0, H^{15}), 3.74 (4H, q, $^3J_{HH}$ 6.9, H^2), 3.55–3.49 (4H, m, H^{11} and H^{12}), 2.11 (3H, s, H^{17}), 2.00 (3H, s, H^{14}), 1.74 (6H, s, H^5), 1.50 (12H, s, H^3), 1.37 (6H, t, $^3J_{HH}$ 6.9, H^1), 1.31 (3H, d, $^3J_{HH}$ 7.0, $H^{16/16'}$), 0.96 (3H, s, $H^{14'}$), 0.94 (3H, d, $^3J_{HH}$ 7.0, $H^{16/16'}$); δ_C (151 MHz, CD_3OD): 169.9, 169.8, 169.1,

168.4, 167.9, 159.5, 157.7, 156.8, 153.7, 150.2, 139.5, 137.4, 135.4, 135.1, 134.2, 132.6, 130.7, 130.2, 128.3, 126.5, 126.1, 125.2, 125.1, 122.9, 122.1, 121.0, 120.7, 115.0, 114.4, 111.7, 96.9, 61.6, 41.2, 41.0, 40.9, 40.1, 33.7, 29.5, 27.0, 23.9, 22.0, 21.4, 18.2, 13.6, 13.1; UV/Vis (MeOH): λ_{max} = 583 nm; fluorescence (MeOH): λ_{ex} = 550 nm; λ_{em} = 612 nm; Φ_{fl} = 68%; HRMS (ESI$^+$) *m/z* 995.5074 [M]$^+$ ($C_{61}H_{67}O_7N_6$ requires 995.5066).

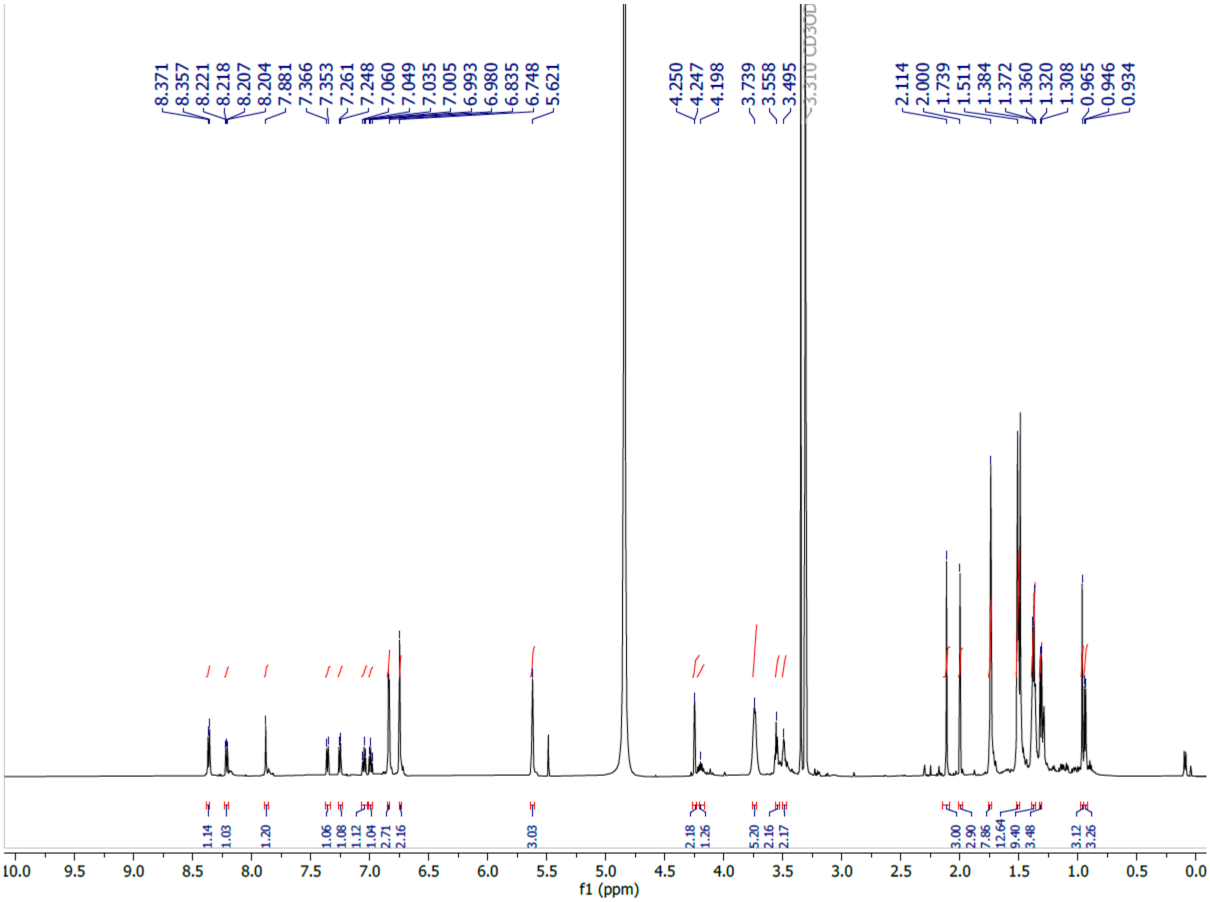

Figure S36. ^{1}H NMR spectrum of **FULG-RhoX** (600 MHz, CD_3OD).

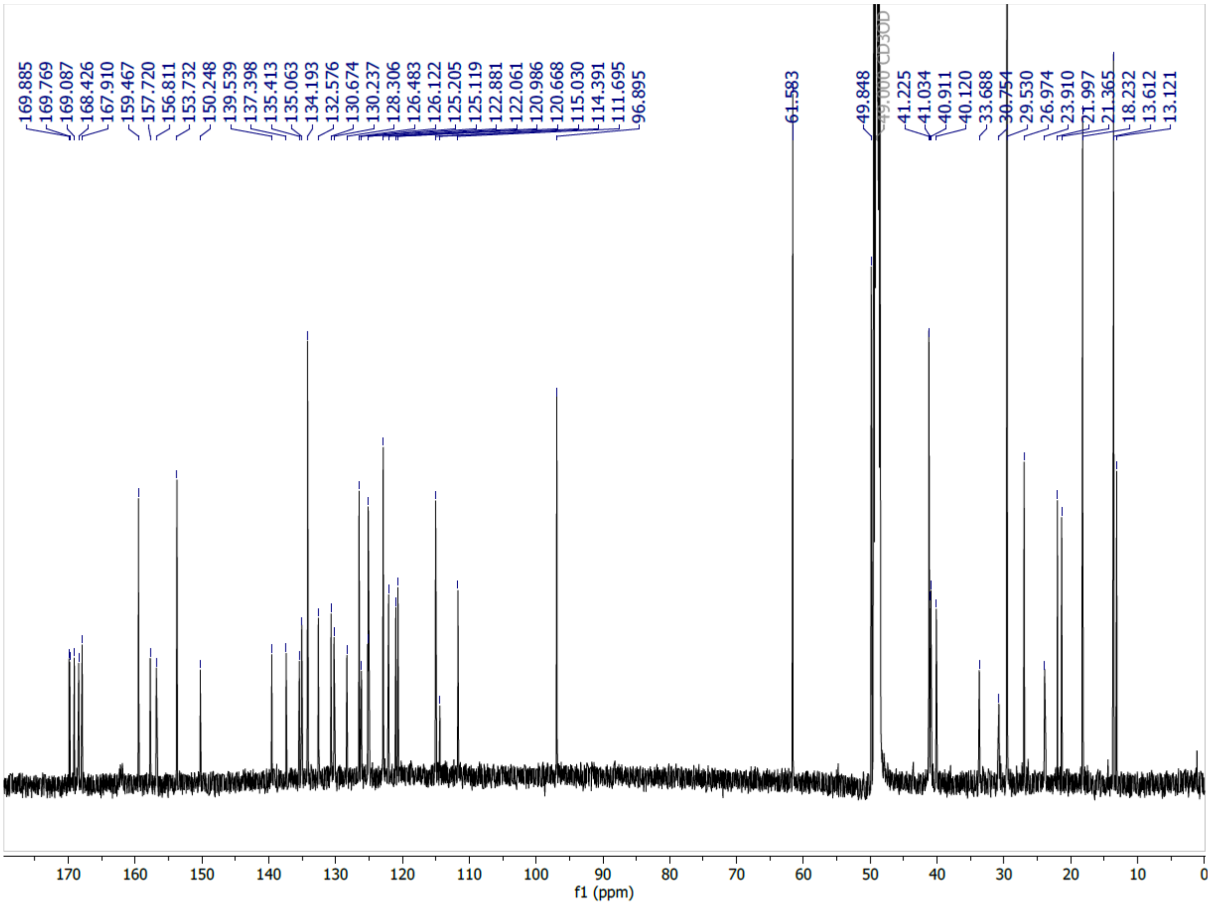

Figure S37. ^{13}C NMR spectrum of **FULG-RhoX** (600 MHz, CD_3OD).

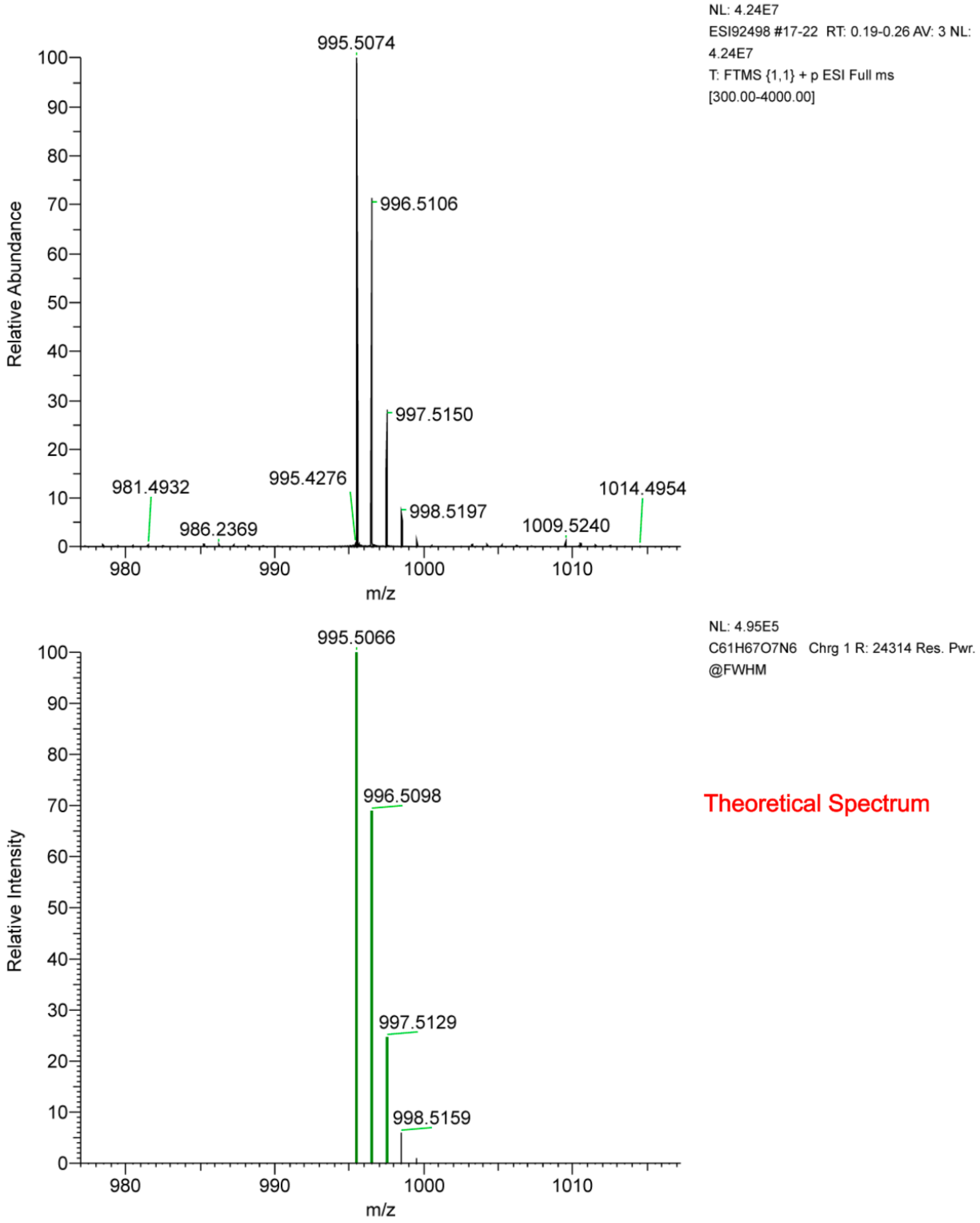

Figure S38. Experimental HRMS (ESI$^+$) pattern of **FULG-RhoX** and the theoretical pattern.

2,3,3-Trimethyl-1-(pent-4-yn-1-yl)-3*H*-indol-1-ium iodide (14)

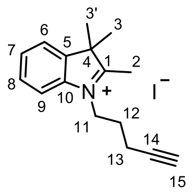

The compound was synthesized following a procedure developed by Gerowska and co-workers with modification in the purification method.[11] 2,3,3-Trimethylindolenine (7.8 mL, 49 mmol) and 5-chloro-1-pentyne (8.0 mL, 76 mmol, 1.6 equiv.) were dissolved in acetonitrile (50 mL). The reaction mixture was heated at reflux for 18 h before being allowed to cool to room temperature. The precipitate was collected by filtration and then washed with Et_2O. The crude product was dissolved in nitromethane and precipitated with EtOAc twice to yield the product as a pale pink solid (3.0 g, 18%). NMR δ_H (400 MHz, DMSO): 7.98 (1H, m, H^9), 7.84 (1H, m, H^6), 7.62 (2H, m, H^7 and H^8), 4.46 (2H, t, $^3J_{HH}$ 7.5, H^{11}), 2.84 (3H, s, H^2), 2.23 (2H, t, $^3J_{HH}$ 7.2, H^{15}), 1.84 (2H, m, H^{12}), 1.61–1.49 (8H, m, H^{14}, H^3 and $H^{3'}$), 1.43 (2H, m, H^{13}); δ_C (101 MHz, DMSO): 196.5, 174.3, 141.9, 141.1, 129.4, 128.9, 123.5, 115.5, 54.2, 47.4, 33.4, 26.9, 25.4, 24.0, 22.0; HRMS (ESI+) m/z: $[M]^+$ calcd. for $C_{17}H_{24}NO_2$ 274.1802, found 274.1791.

Hemicyanine-alkyne (15)

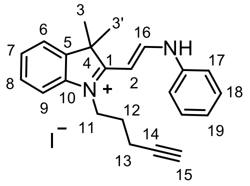

114(2.50 g, 7.08 mmol) and *N*,*N'*-diphenylformamidine (1.53 g, 7.78 mmol, 1.1 equiv.) were suspended in ethanol (40 mL). Triethylorthoformate (1.15 g, 7.78 mmol, 1.1 equiv.) was then added and the suspension was heated at reflux for 15 h. The reaction mixture was cooled to room temperature. The remaining oil was dissolved in EtOH and precipitated with Et_2O to yield the product as a dark red solid (3.2 g, 99%). NMR δ_H (500 MHz, CD_3CN): 11.26 (1H, s, NH), 8.61 (1H, d, $^3J_{HH}$ 12.5, H^2), 7.56–7.25 (9H, m, ArH), 6.97 (1H, d, $^3J_{HH}$ 12.5, H^{16}), 4.16 (2H, t, $^3J_{HH}$ 7.7, H^{11}), 2.46 (2H, td, $^3J_{HH}$ 7.7, $^3J_{HH}$ 2.7, H^{13}), 2.33 (1H, t, $^3J_{HH}$ 2.7, H^{15}), 2.04 (2H, appr. p., H^{12}), 1.70 (6H, s, H^3 & $H^{3'}$); δ_C (126 MHz, CD_3CN): 179.6, 152.7, 142.8, 142.3, 139.3, 130.8, 129.7, 127.5, 127.0, 123.5, 119.0, 112.5, 92.0, 83.8, 71.2, 57.9, 50.8, 44.8, 28.6, 26.7, 18.7, 16.7; HRMS (ESI^+) *m/z*: $[M^+]$ calcd. for $C_{23}H_{25}N_2^+$ 363.2012; found 329.2000.

Cy3-alkyne

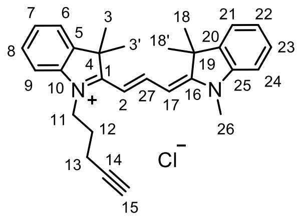

15 (3.20 g, 7.01 mmol), 1,2,3,3-tetramethyl-3*H*-indolium iodide (3.17 g, 10.5 mmol, 1.5 equiv.) and acetic anhydride (6.6 mL, 70.1 mmol, 10 equiv.) were dissolved in pyridine (40 mL). The reaction mixture was stirred at 50 °C for 18 h before being cooled to room temperature and precipitated with diethyl ether (500 mL, 4 °C) overnight. The supernatant liquid layer was decanted, and the remaining thick dark-brown oil was redissolved in chloroform. The chloroform solution was subsequently washed with HCl (aq. 0.5 M) and brine, dried ($MgSO_4$) and concentrated in vacuo. The crude product was purified by flash column chromatography (CH_2Cl_2, CH_2Cl_2 1:1 EtOAc, CH_2Cl_2 1:2 EtOAc and 1–5% MeOH in CH_2Cl_2 to yield the product as a shiny green solid (2.20 g, 59%). NMR δ_H (500 MHz, CD_3OD): 8.56 (1H, appr. t, H^{27}), 7.58–7.26 (8H, m, $H^{6–9}$ & $H^{21–24}$), 6.56–6.43 (2H, m, H^2 & H^{17}), 4.27 (2H, t, ${}^3J_{HH}$ 7.6, H^{11}), 3.70 (3H, s, H^{26}), 2.51 (1H, t, ${}^3J_{HH}$ 2.6, H^{15}), 2.43–2.38 (2H, td, ${}^3J_{HH}$ 6.8, ${}^3J_{HH}$ 2.6, H^{13}), 2.04 (2H, appr. p, H^{12}), 1.79–1.75 (12H, two singlets overlapping, H^3, $H^{3'}$, H^{18} & $H^{18'}$); δ_C (126 MHz, CD_3OD): 176.9, 176.0, 152.2, 144.1, 143.4, 142.2, 142.1, 130.0, 129.8, 126.9, 126.7, 123.6, 123.41, 112.4, 112.2, 104.0, 103.6, 83.7, 71.4, 50.7, 50.6, 44.1, 31.9, 28.4, 28.1, 27.3, 23.8; HRMS (ESI$^+$) *m/z*: [M$^+$] calcd. for $C_{29}H_{33}N_2^+$ 409.2638; found 409.2626; UV-Vis (water): λ_{max} (ε) = 541 nm (154,000 mol^{-1} dm^3 cm^{-1}); fluorescence (water): λ_{em} = 556 nm; $\Phi_{fl.}$ = 2.9%.

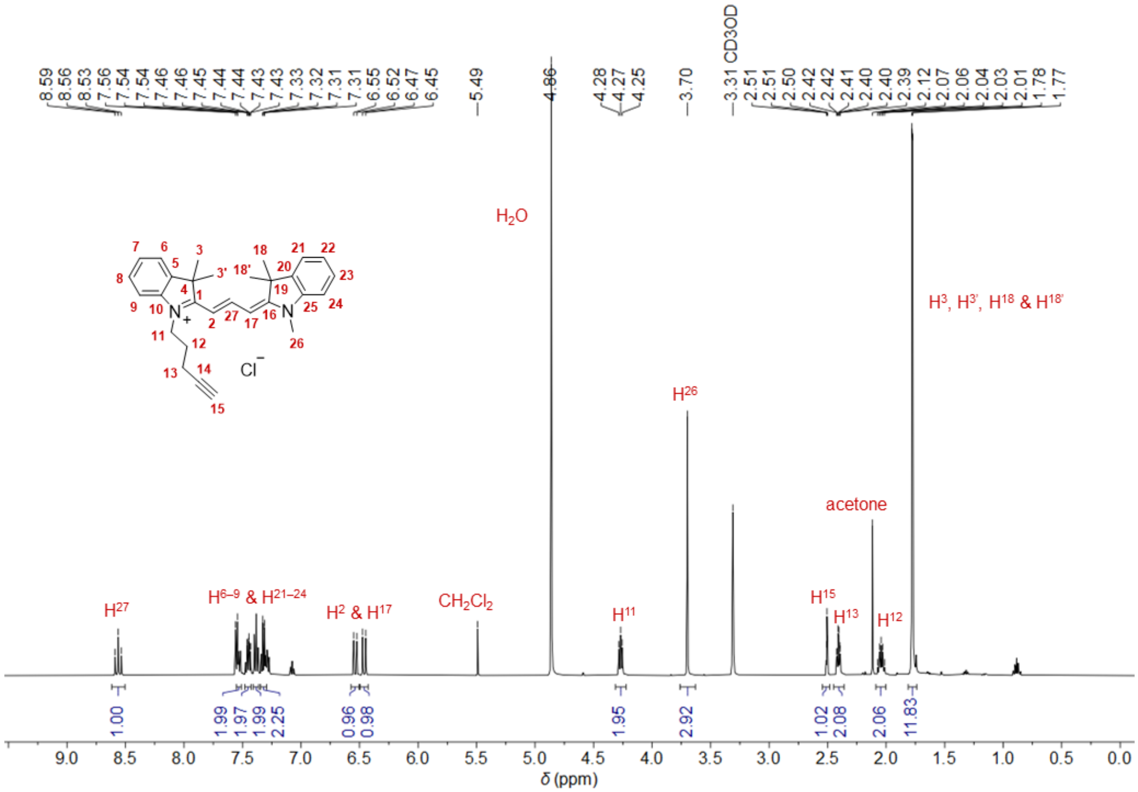

Figure S39. ^{1}H NMR spectrum (500 MHz, CD_3OD) of **Cy3-alkyne**.

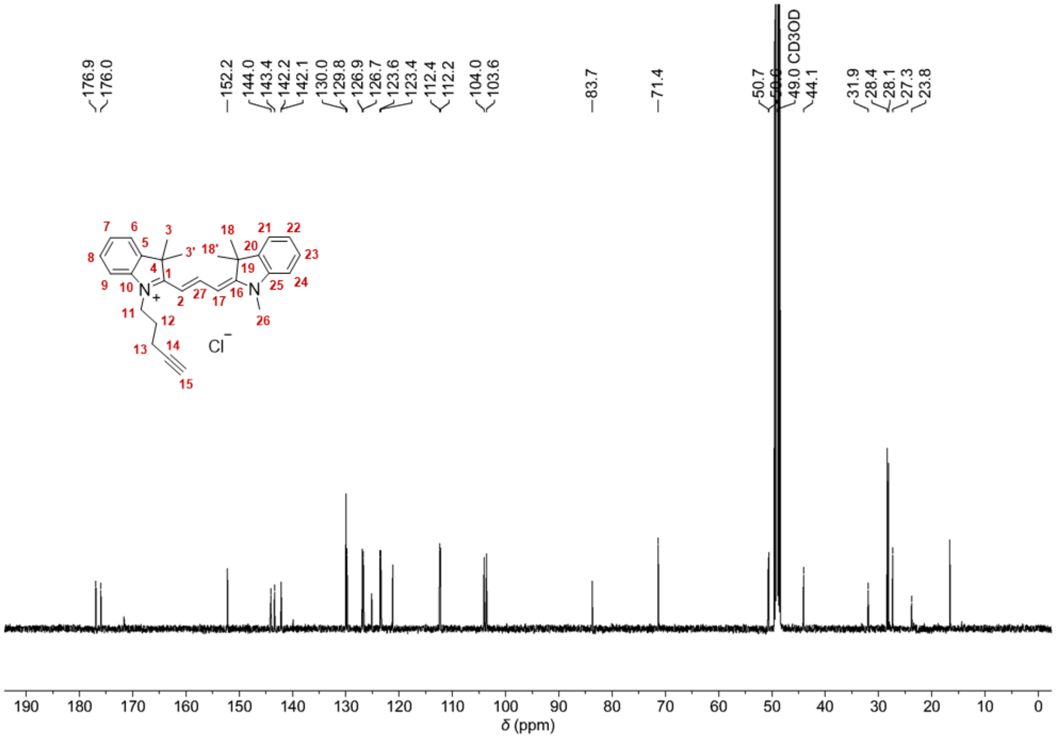

Figure S40. ^{13}C NMR spectrum (126 MHz, CD_3OD) of **Cy3-alkyne**.

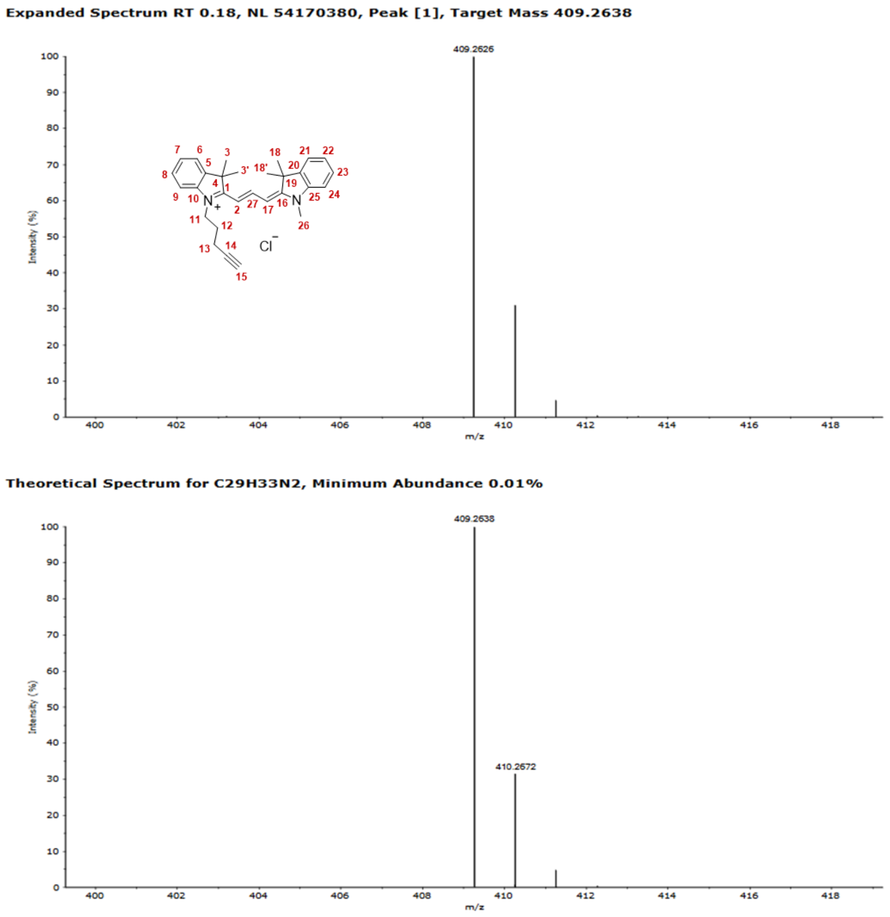

Figure S41. Experimental HRMS (ESI+) pattern of **Cy3-alkyne** and the theoretical pattern.

Cy3-iodo

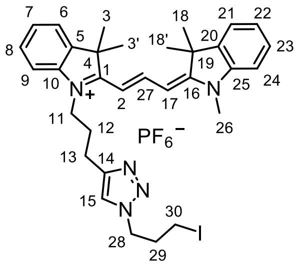

Cy3-alkyne (500 mg, 1.12 mmol), 1-azido-3-iodopropane (2.37 g, 11.2 mmol, 10 equiv.), copper(I) iodide (107 mg, 562 mmol, 0.5 equiv.) and DIPEA (200 µL, 1.15 mmol, 1 equiv.) were dissolved in CH_2Cl_2 (100 mL). The reaction mixture was heated at 30 °C for 40 h and concentrated in vacuo. The crude product was purified by flash column chromatography (CH_2Cl_2 1:1 EtOAc, then 1–5% MeOH in CH_2Cl_2). The purified compound was dissolved in CH_2Cl_2 and washed with an aqueous solution of potassium hexafluorophosphate (5% w/v) to exchange the counterion to hexafluorophosphate. The solution was concentrated in vacuo to yield the final compound as a shiny green solid (250 mg, 30%). NMR δ_H (500 MHz, CD_3OD): 8.54 (1H, appr. t, ${}^3J_{HH}$ 13.5, H^{27}), 7.86 (1H, s, H^{15}), 7.58–7.28 (8H, m, $H^{6–9}$ & $H^{21–24}$), 6.50–6.43 (2H, 2×d, ${}^3J_{HH}$ 13.5, H^2 & H^{17}), 4.49 (2H, t, ${}^3J_{HH}$ 6.7, H^{30}), 4.23 (2H, t,

$^3J_{HH}$ 7.6, H[11]), 3.73 (3H, s, H[26]), 3.18 (2H, t, $^3J_{HH}$ 6.7, H[28]), 2.92 (2H, t, $^3J_{HH}$ 7.6, H[13]), 2.38 (2H, appr. p., $^3J_{HH}$ 6.7, H[29]), 2.25 (2H, appr. p, $^3J_{HH}$ 7.6, H[12]), 1.78–1.75 (12H, 2×s, H[3], H[3'], H[18] & H[18']); δ_C (126 MHz, CD_3OD): 176.8, 175.9, 152.1, 147.7, 144.1, 143.3, 142.1, 130.0, 129.9, 126.8, 126.7, 124.1, 123.6, 123.4, 112.4, 112.3, 104.1, 103.8, 54.8, 51.5, 50.7, 50.6, 44.6, 34.9, 32.0, 30.7, 28.3, 28.2, 27.7, 23.4. HRMS (ESI$^+$) *m/z*: [M$^+$] calcd. for $C_{32}H_{39}IN_5^+$ 620.2245; found 620.2237.

DTE-py-Cy3

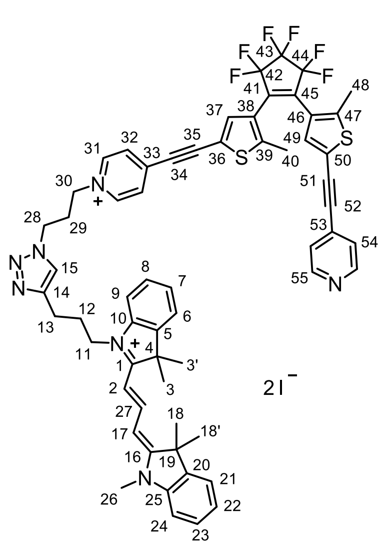

Cy3-iodo (100 mg, 134 µmol, 1 equiv.) and **DTE-dipy** (763 mg, 10 equiv.) were dissolved in DMF (20 mL). The reaction mixture was stirred at 45 °C for 45 h. Upon confirmation of full consumption of **Cy3-iodo** and the formation of the dyad by analytical HPLC (monitored at 360 nm corresponding to DTE absorption and 550 nm corresponding to cyanine absorption), the reaction mixture was concentrated in vacuo and purified by flash column chromatography (CH_2Cl_2 1:1 EtOAc, then 1–10% MeOH in CH_2Cl_2) to remove excess **DTE-dipy** used in the reaction. The purified product was dissolved in CH_2Cl_2 and the solution was washed with an aqueous solution of sodium iodide (10% w/v). The washed organic layer was concentrated in vacuo to yield the product as a black solid (55 mg, 31%). The compound was analyzed by analytical HPLC to confirm full removal of **DTE-dipy** and directly used in the next step. HPLC t_R = 14.4 min (in both 360 nm and 550 nm channels).

DTE-Cy3

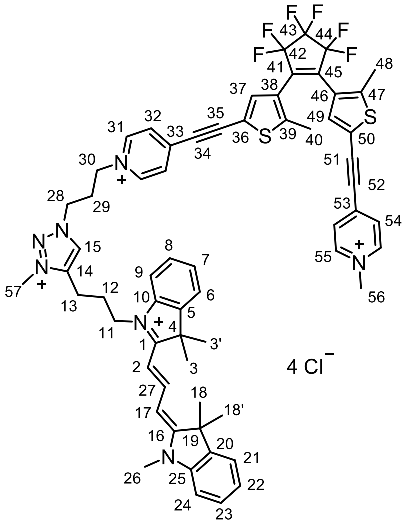

DTE-py-Cy3 (30 mg, 14.8 μmol) was dissolved in a mixture of iodomethane (5 mL) and CH_2Cl_2 (5 mL). The reaction mixture was stirred at 20 °C for 3 days. Upon full consumption of the starting material **DTE-py-Cy3** and conversion into a new dyad species verified by analytical HPLC, the reaction mixture was concentrated *in vacuo*. The crude product was purified by reverse phase flash column chromatography (10–80% MeOH in water). The purified product was dissolved in MeCN (1 mL) and added to an aqueous solution of potassium hexafluorophosphate (5% w/v, 50 mL). The precipitate (**DTE-Cy3** as an hexafluorophosphate salt) was collected and washed with water. The resulting solid was dissolved in MeCN (1 mL) and added to a solution of tetrabutylammonium chloride in acetone (10% w/v, 20 mL). The mixture was stirred at room temperature for 18 h to exchange the counterion to chloride. The precipitate was collected to yield the product as a black solid (15 mg, 82%). NMR δ_H (600 MHz, CD_3OD): 9.05 (2H, d, $^3J_{HH}$ 7.0, H^{31}), 8.86 (2H, d, $^3J_{HH}$ 6.8, H^{55}), 8.80 (1H, s, H^{15}), 8.58 (1H, appr. t, $^3J_{HH}$ 13.5, H^{27}), 8.15 (2H, d, $^3J_{HH}$ 7.0, H^{32}), 8.11 (2H, d, $^3J_{HH}$ 6.8, H^{54}), 7.69–7.66 (2H, 2×s, H^{37} & H^{49}), 7.57–7.29 (8H, m, $H^{6–9}$ & $H^{21–24}$), 6.69–6.61 (2H, 2×d, $^3J_{HH}$ 13.5, H^2 & H^{17}), 4.79 (m, overlapping with water peak, confirmed with COSY, H^{30}), 4.41–4.29 (10H, m incl. 2×s, H^{11}, H^{28}, H^{56} & H^{57}), 3.73 (3H, s, H^{26}), 3.19 (2H, t, $^3J_{HH}$ 8.3, H^{13}), 2.77 (2H, appr. p, $^3J_{HH}$ 7.7, H^{29}), 2.34 (2H, appr. p, $^3J_{HH}$ 8.3, H^{12}), 2.07–2.05 (6H, 2×s, H^{40} & H^{48}), 1.82–1.75 (12H, 2×s, H^3, $H^{3'}$, H^{18} & $H^{18'}$); δ_C (151 MHz, CD_3OD): 177.2, 175.7, 152.4, 150.1, 149.9, 146.6, 146.0, 145.5, 144.0, 143.3, 142.2, 142.1, 141.5, 140.7, 137.2, 137.0, 130.4, 130.2, 130.1, 130.0, 129.9, 127.1, 126.8, 126.7, 123.6, 123.4, 120.4, 120.3, 112.5, 112.2, 104.7, 103.7, 96.9, 96.2, 91.1, 90.9, 59.0, 51.4, 50.8, 50.5, 44.2, 38.4, 32.1, 31.4, 28.5, 28.1, 25.8, 24.8, 24.2, 21.9, 20.7, 14.8, 14.8, 13.9; HRMS (ESI$^+$) *m/z*: [M^{4+}] calcd. for $C_{63}H_{61}F_6N_7S_2^{4+}$ 273.3584; found 273.3580; HPLC: t_R = 12.6 min (in both 360 nm and 560 nm channels); UV-Vis (water): $\lambda_{max,\ DTE}$ (ε) = 377 nm (71200 mol^{-1} dm^3 cm^{-1}); $\lambda_{max,\ cyanine}$ (ε) = 541 nm (154,000 mol^{-1} dm^3 cm^{-1}); fluorescence (water): λ_{em} = 556 nm; $\Phi_{fl.}$ = 2.5%.

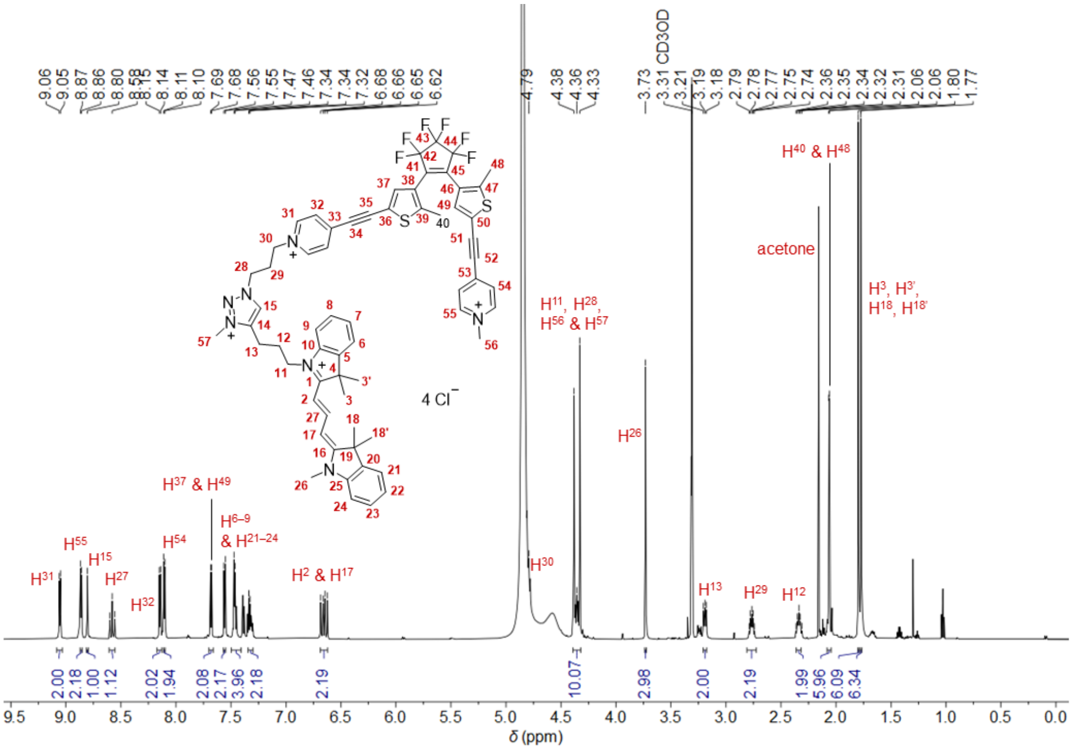

Figure S42. ^{1}H NMR spectrum (600 MHz, CD_3OD) of **DTE-Cy3**.

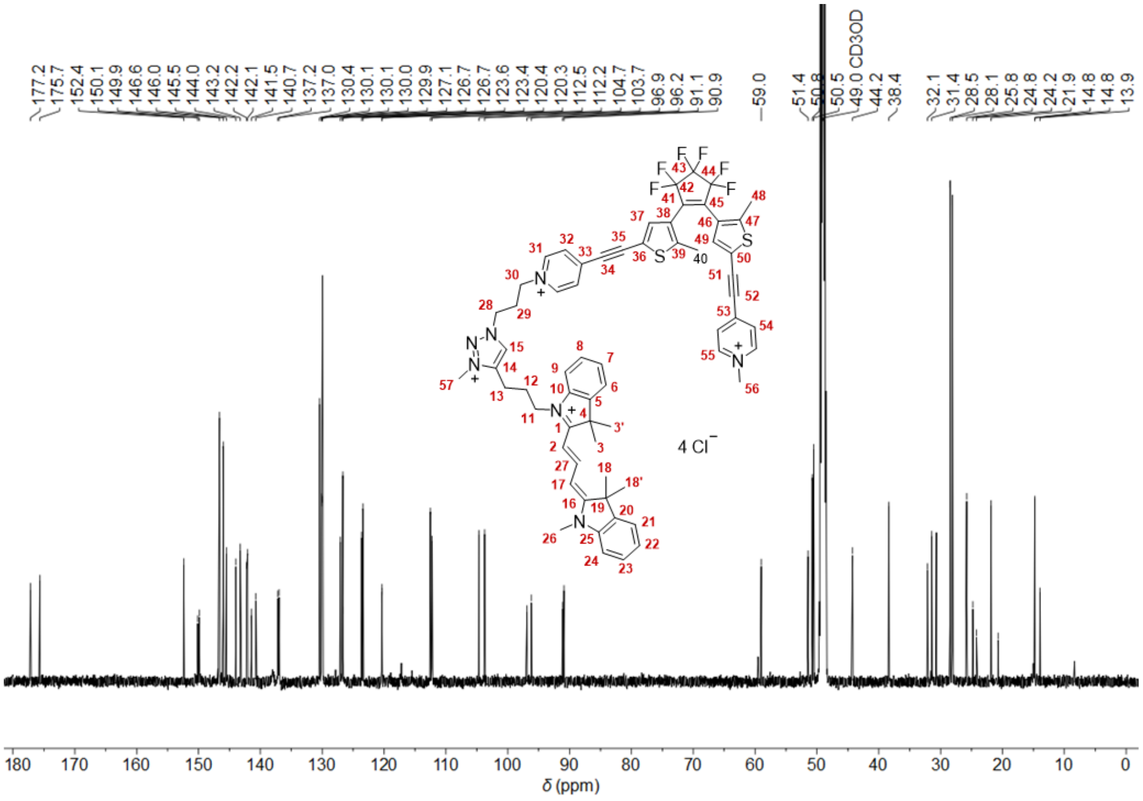

Figure S43. ^{13}C NMR spectrum (151 MHz, CD_3OD) of **DTE-Cy3**.

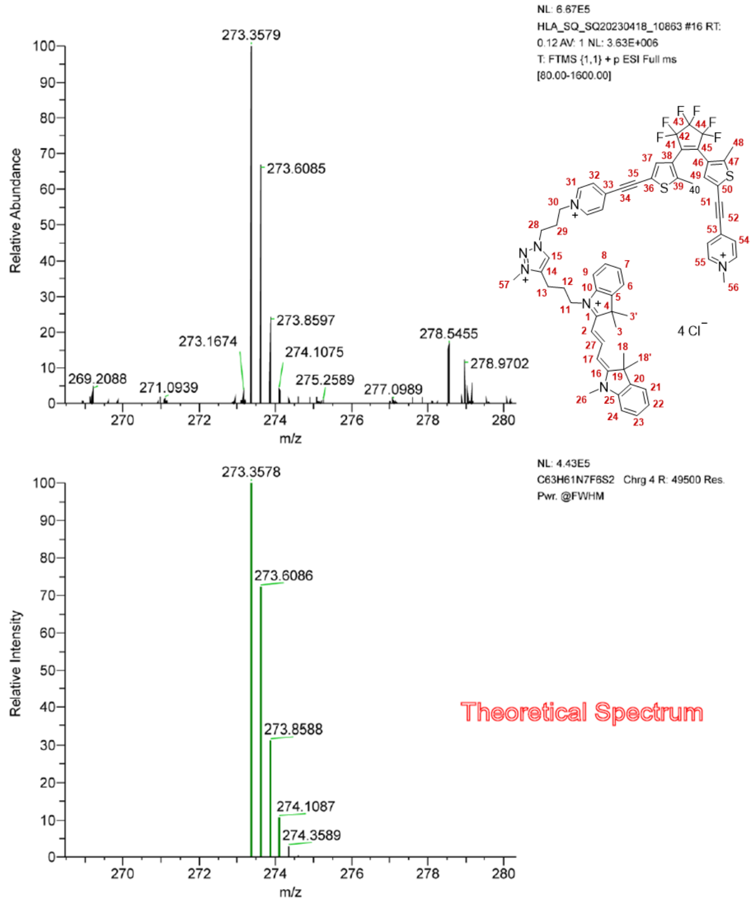

Figure S44. Experimental HRMS (ESI⁺) pattern of **DTE-Cy3** and the theoretical pattern.

2-(4-(Carboxymethyl)phenyl)hydrazin-1-ium chloride (17)

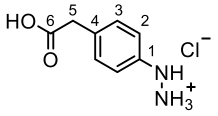

The compound was synthesized following the procedure developed by Ahlström and co-workers.[7] 2-(4-Aminophenyl)acetic acid (10.0 g, 66.2 mmol) was dissolved in aqueous hydrochloric acid (37%, 100 mL). The slurry was heated to 120 °C until a clear solution was formed. The solution was rapidly cooled down to −5 °C. A solution of $NaNO_2$ (5.48 g, 79.4 mmol, 1.2 equiv.) in water (5 mL) was then added dropwise and the mixture was stirred at −5 °C for 15 min. A solution of $SnCl_2{\cdot}2H_2O$ (29.9 g, 132 mmol, 2 equiv.) in HCl (37%, aq., 10 mL) was further added to the reaction mixture at −20 °C. After stirring at −20°C for 25 min and at −5°C for another 25 min, the precipitate was collected by filtration and successively washed with ethanol to yield the product as an off-white solid (12.1 g, 91%). NMR δ_H (500 MHz, DMSO-d_6): 10.07 (s, br, COOH and NH_3^+), 8.15 (1H, s, NH), 7.17 (2H, d, $^3J_{HH}$ 8.7, H^3), 6.90 (2H, d, $^3J_{HH}$ 8.7, H^2), 3.48 (2H, s, H^5); HRMS (ESI⁺) *m/z*: $[M]^+$ calcd. for $C_8H_{11}N_2O_2^+$ 167.0815; found 167.0813.

2-(2,3,3-Trimethyl-3H-indol-5-yl)acetic acid (18)

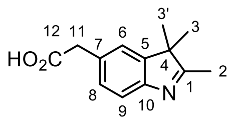

17 (12.0 g, 59.2 mmol), 3-methylbutan-2-one (12.8 g, 148 mmol, 2.5 equiv.) and potassium acetate (11.6 g, 118 mmol, 2 equiv.) were dissolved acetic acid (200 mL). The reaction mixture was heated at 90 °C for 3 h before being allowed to cool to room temperature. The reaction mixture was concentrated in vacuo, dissolved in CH_2Cl_2 and washed with water and brine. The organic layer was dried ($MgSO_4$), filtered and concentrated in vacuo to yield the product as a pale red solid (6.50 g, 51%). No purification was required. NMR δ_H (500 MHz, DMSO-d$_6$): 12.28 (1H, s, br, COOH), 7.34 (1H, d, $^3J_{HH}$ 7.8, H^8), 7.29 (1H, s, H^6), 7.15 (1H, d, $^3J_{HH}$ 7.8, H^9), 3.57 (2H, s, H^{11}), 2.19 (3H, s, H^2), 1.23 (6H, s, H^3 & $H^{3'}$); δ_C (126 MHz, DMSO-d$_6$): 187.4, 172.9, 152.4, 146.0, 131.6, 128.5, 122.7, 118.9, 53.1, 40.7, 22.6, 15.0. HRMS (ESI$^+$) m/z: [M+H]$^+$ calcd. for $C_{13}H_{16}NO_2^+$ 218.1176; found 218.1170.

1-(2-(1,3-Dioxolan-2-yl)ethyl)-5-(carboxymethyl)-2,3,3-trimethyl-3H-indol-1-ium iodide (19)

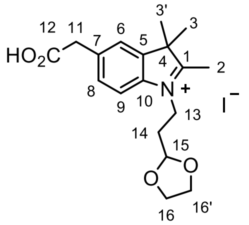

18 (2.80 g, 12.9 mmol), 2-(2-bromoethyl)-1,3-dioxolane (11.7 g, 64.4 mmol, 5 equiv.) and potassium iodide (6.42 g, 38.7 mmol, 3 equiv.) were dissolved in acetonitrile (50 mL). The reaction mixture was heated at 80 °C for 18 h before being allowed to cool to room temperature. The reaction mixture was concentrated in vacuo. The resulting slurry was dissolved in CH_2Cl_2 and then filtered to remove inorganic salts. After concentrating the solution in vacuo, the remaining oil was dissolved in MeCN and precipitated with EtOAc to yield the product as a dark red solid (1.8 g, 31%). NMR δ_H (500 MHz, DMSO-d$_6$): 7.85 (1H, d, J_{HH} 8.2, H^9), 7.72 (1H, s, H^6), 7.51 (1H, d, J_{HH} 8.2, H^8), 4.99 (1H, t, J_{HH} 4.3, H^{15}), 4.56 (2H, t, J_{HH} 6.6, H^{13}), 3.84–3.70 (8H, m, incl. a singlet at 3.74, H^{16}, $H^{16'}$ & H^2), 2.79 (2H, s, H^{11}), 2.24 (2H, m, H^{14}), 1.51 (6H, s, H^3 & $H^{3'}$); δ_C (126 MHz, DMSO-d$_6$): 172.2, 141.7, 139.7, 136.8, 130.2, 124.5, 115.1, 101.4, 64.2, 59.7, 54.1, 43.3, 30.5, 21.8, 20.8, 14.1; HRMS (ESI$^+$) m/z: [M$^+$] calcd. for $C_{18}H_{24}NO_4^+$ 318.1700; found 318.1696.

1-(2-(1,3-Dioxolan-2-yl)ethyl)-2,3,3-trimethyl-3*H*-indol-1-ium iodide (21)

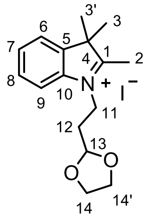

The compound was synthesized following a patent assigned to SFC Co., Ltd. with longer reaction time and modification in the purification method.[12] 2-(2-Bromoethyl)-1,3-dioxolane (4.43 g, 24.5 mmol, 1.3 equiv.) and potassium iodide (9.38 g, 3 equiv.) were stirred in acetonitrile (100 mL) at 50 °C for 1 hour. To the reaction mixture was added 2,3,3-trimethylindolenine (3.00 g, 18.8 mmol, 1 equiv.). The resulting reaction mixture was stirred at 85 °C for 36 h before being cooled to room temperature. The insoluble inorganic salt was filtered off and the filtrate was concentrated in vacuo. The crude product was redissolved in acetonitrile (15 mL) and precipitated with CH_2Cl_2 (7.5 mL) to remove the last trace of inorganic salt. The filtrate was concentrated in vacuo, further redissolved in acetonitrile (10 mL) and precipitated with EtOAc (50 mL) overnight at 4 °C. The supernatant was decanted and the remaining solid was dried to yield the final product as a pale brown solid (2.73 g, 38%). δ_H (500 MHz, CD_3CN): 7.80–7.75 (1H, m, H^9), 7.74–7.70 (1H, m, H^6), 7.66–7.58 (2H, m, H^7 & H^8), 4.99 (1H, t, J_{HH} 4.1, H^{13}), 4.54 (2H, t, J_{HH} 6.6, H^{11}), 3.84–3.74 (4H, m, H^{14} & $H^{14'}$), 2.76 (3H, s, H^2), 2.35–2.30 (2H, td, J_{HH} 4.1, J_{HH} 6.6, H^{12}), 1.54 (6H, s, H^3 & $H^{3'}$); δ_C (126 MHz, CD_3CN): 197.9, 142.9, 142.1, 130.8, 130.2, 124.41, 116.4, 102.4, 65.6, 55.6, 44.6, 31.3, 22.5, 15.3. HRMS (ESI$^+$) *m/z*: $[M]^+$ calcd. for $C_{16}H_{22}NO_2^+$ 260.1645; found 260.1639.

(*E*)-1-(2-(1,3-dioxolan-2-yl)ethyl)-3,3-dimethyl-2-(2-(phenylamino)vinyl)-3*H*-indol-1-ium iodide (22)

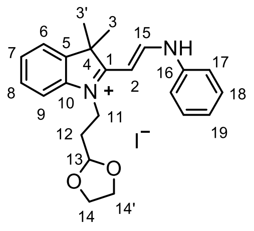

21 (2.35 g, 6.07 mmol) and *N*,*N*'-diphenylformamidine (1.31 g, 6.67 mmol, 1.1 equiv.) were suspended in ethanol (40 mL). Triethylorthoformate (989 mg, 6.67 mmol, 1.1 equiv.) was then added and the suspension was heated at reflux for 15 h. The reaction mixture was cooled to room temperature and concentrated in vacuo. The remaining oil was dissolved in EtOH and precipitated with Et_2O to yield the product as a dark red solid (2.40 g, 81%). NMR δ_H (500 MHz, CD_3CN): 8.59 (1H, d, J_{HH} 12.5, H^{15}), 7.56–7.27 (9H, m, ArH), 6.82 (1H, d, J_{HH} 12.5, H^2), 5.05 (1H, t, J_{HH} 4.0, H^{13}), 4.21 (2H, t, J_{HH} 7.3, H^{11}), 3.93–3.75 (4H, m, H^{14} & $H^{14'}$), 2.22 (2H, m, H^{12}), 1.69 (6H, s, H^3 & $H^{3'}$); δ_C (126 MHz, CD_3CN): 179.5, 152.5, 142.7, 142.1, 139.4, 130.8, 129.6, 127.3, 126.91, 123.3, 118.9, 112.8, 102.5, 92.0, 65.7, 50.9, 40.9, 31.2, 22.4; HRMS (ESI$^+$) *m/z*: $[M^+]$ calcd. for $C_{23}H_{27}N_2O_2^+$ 363.2057; found 363.2067.

1-(2-(1,3-Dioxolan-2-yl)ethyl)-2-((*E*)-3-((*E*)-1-(2-(1,3-dioxolan-2-yl)ethyl)-3,3-dimethylindolin-2-ylidene)prop-1-en-1-yl)-5-(carboxymethyl)-3,3-dimethyl-3H-indol-1-ium hexafluorophosphate (23)

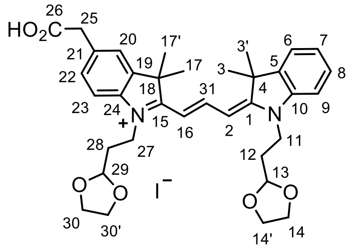

22 (2.50 g, 5.10 mmol) and **20** (2.72 g, 6.12 mmol, 1.2 equiv.) were dissolved in a mixture of pyridine (30 mL), acetic acid (30 mL) and acetic anhydride (6.67 mL) (pyridine : acetic acid : acetic anhydride = 4.5 : 4.5 : 1). The reaction mixture was heated at 50 °C for 18 h before being allowed to cool to room temperature. The reaction mixture was precipitated with cold ether. The precipitate was purified by flash column chromatography (DCM, DCM/EtOAc 1:1, DCM/EtOAc 1:2, 1%–5% MeOH in DCM) to yield the product as a shiny green solid (1.7 g, 46%). NMR δ_H (500 MHz, CD_3CN): 8.45 (1H, dd, appr. t, J_{HH} 13.6, H^{31}), 7.54–7.21 (7H, m, H^6, H^7, H^8, H^9, H^{20}, H^{22} & H^{23}), 6.35–6.28 (2H, d × 2, J_{HH} 13.6, H^2 & H^{16}), 4.98 (2H, t, J_{HH} 4.0, H^{13} & H^{29}), 4.17 (4H, t × 2, appr. q, H^{11} & H^{27}), 3.95–3.79 (8H, m, H^{14}, $H^{14'}$, H^{30} & $H^{30'}$), 3.68 (2H, s, H^{25}), 2.22–2.11 (H^{12} & H^{28} under water peak), 1.72–1.69 (12H, s × 2, H^3, $H^{3'}$, H^{17} & $H^{17'}$); δ_C (126 MHz, CD_3CN): 175.6, 173.0, 151.3, 143.1, 142.1, 142.1, 141.9, 133.2, 130.8, 129.6, 126.4, 124.6, 123.3, 112.3, 112.2, 103.5, 103.5, 102.6, 79.1, 65.7, 50.3, 40.6, 40.4, 40.3, 31.4, 31.4, 28.0; HRMS (ESI$^+$) *m/z*: [M$^+$] calcd. for $C_{35}H_{43}N_2O_6^+$ 587.3104; found 587.3116.

Cy3B(chloride)

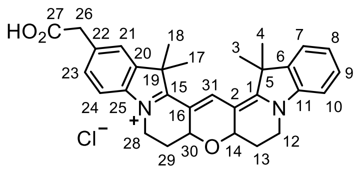

To a solution of **23** (1.20 g, 1.68 mmol) in chloroform (100 mL) was added aqueous sulfuric acid (50%, 20 mL). The reaction mixture was stirred at 25 °C for 30 min. The reaction mixture was then diluted with chloroform (100 mL), washed with water, and then washed with 0.5 M aqueous HCl to exchange the counterion to chloride. The organic layer was collected, dried ($MgSO_4$) and concentrated in vacuo. The crude product was purified by flash column chromatography (CH_2Cl_2 to 5% MeOH in CH_2Cl_2) to yield the product as a shiny green solid (600 mg, 57%). NMR δ_H (500 MHz, CD_3CN): 8.10 (1H, s, H^{31}), 7.51 (1H, d, J_{HH} 7.4, H^{10}), 7.45–7.40 (2H, m, H^8 & H^{21}), 7.34–7.27 (2H, m, H^9 & H^{24}), 7.23 (1H, d, J_{HH} 7.7, H^7), 7.18 (1H, d, J_{HH} 7.9, H^{23}), 4.60 (2H, m, H^{14} & H^{30}), 4.22 (2H, m, equatorial H^{12} & H^{28}), 3.86 (2H, m, axial H^{12} & H^{28}), 3.68 (2H, s, H^{26}), 2.52 (2H, m, equatorial H^{13} & H^{29}), 1.98 (2H, m, axial H^{13} & H^{29}), 1.72 (12H, m, H^3, H^4, H^{17} & H^{18}); δ_C (126 MHz, CD_3CN): 173.2, 169.4, 169.3, 143.0, 142.1, 142.2, 141.9, 138.9, 133.5, 131.0, 129.7, 126.5, 124.6, 123.3, 111.7, 111.5, 110.7, 110.6, 70.7, 70.6, 49.7, 42.3,

42.2, 40.8, 28.3, 28.2, 27.7, 27.3, 27.2; HRMS (ESI$^+$) *m/z*: [M$^+$] calcd. for $C_{31}H_{33}N_2O_3^+$ 481.2486; found 481.2474; UV-Vis (water): λ_{max} (ε) = 553 nm (156,000 mol^{-1} dm^3 cm^{-1}); fluorescence (water): λ_{em} = 564 nm; $\Phi_{fl.}$ = 63%.

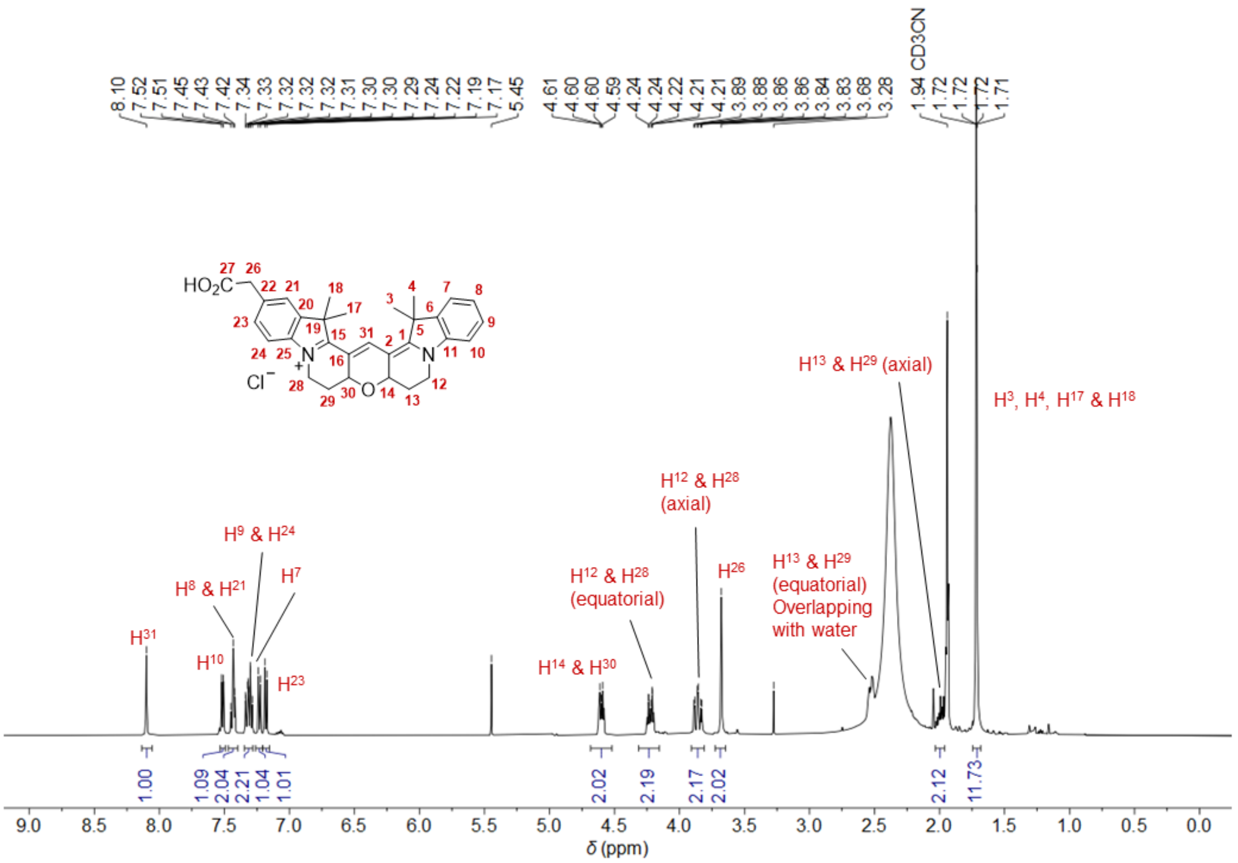

Figure S45. ^{1}H NMR spectrum (500 MHz, CD_3CN) of **Cy3B**.

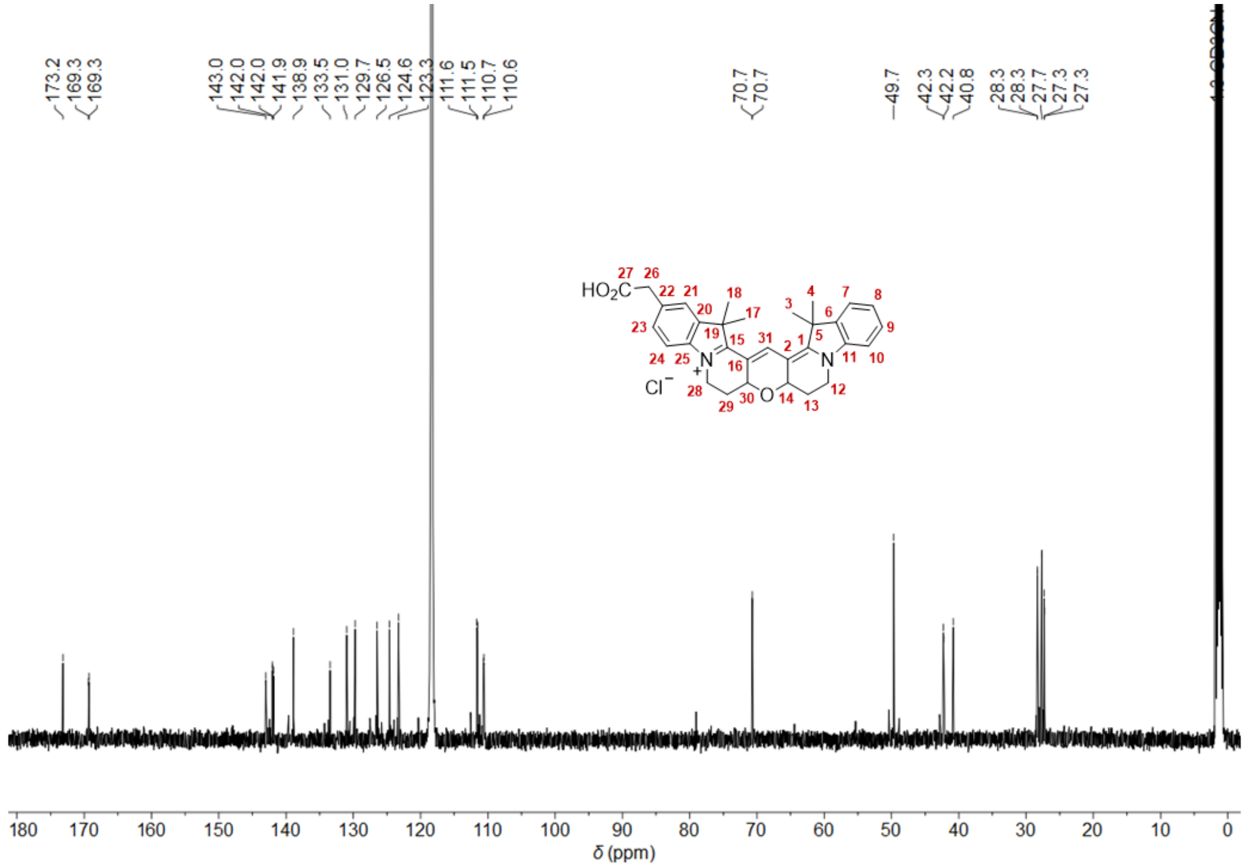

Figure S46. ^{13}C NMR spectrum (126 MHz, CD_3CN) of **Cy3B**.

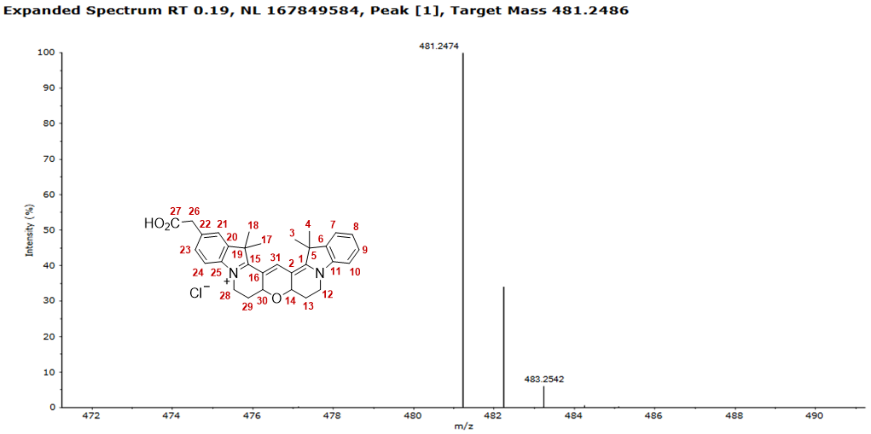

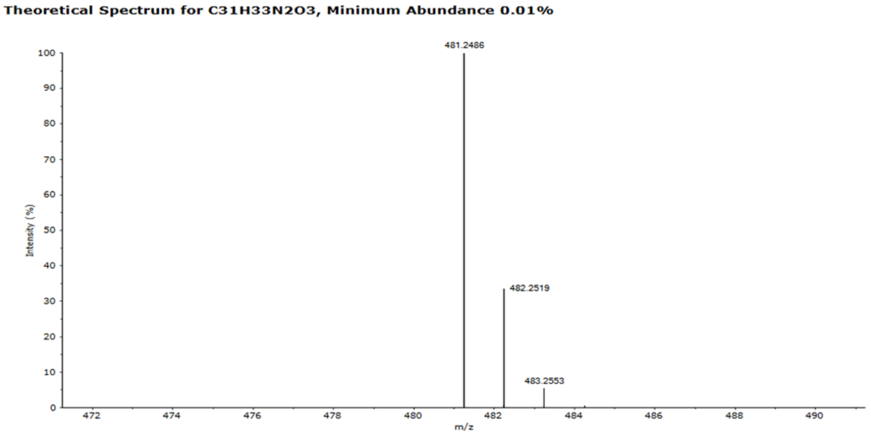

Figure S47. Experimental HRMS (ESI+) pattern of **Cy3B** and the theoretical pattern.

Cy3B-NHS ester (hexafluorophosphate)

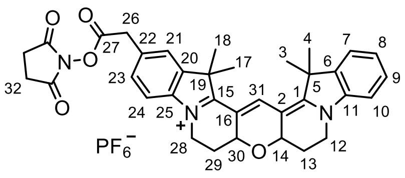

Cy3B (chloride, 275 mg, 534 μmol) was dissolved in MeCN and added to an aqueous solution of potassium hexafluorophosphate (5%) to exchange the counter ion to hexafluorophosphate. The precipitate (320 mg, 96%) was filtered and used in further reactions. To a solution of **Cy3B** (hexafluorophosphate, 200 mg, 319 μmol) in CH_2Cl_2 (5 mL) was added DIPEA (110 μL, 638 μmol, 2 equiv.) and *N*,*N*'-disuccinimidyl carbonate (98 mg, 383 μmol, 1.2 equiv.). The reaction mixture was stirred at 25 °C for 2 h. Upon the completion verified by analytical HPLC (method A), the reaction mixture was diluted (with 50 mL CH_2Cl_2), washed with water, and then washed with an aqueous solution of KPF_6 (5% w/v). The organic layer was separated and concentrated in vacuo. The crude product was purified by

flash column chromatography (gradient from CH_2Cl_2 to 5% MeOH in CH_2Cl_2) to yield the product as a dark brown solid (180 mg, 78%). NMR δ_H (400 MHz, CD_3CN): 8.11 (1H, s, H^{31}), 7.54–7.19 (7H, m, H^{7-10}, H^{21}, H^{23} & H^{24}), 4.69 (2H, m, H^{14} & H^{30}), 4.23 (2H, m, equatorial H^{12} & H^{28}), 4.04 (2H, s, H^{26}), 3.87 (2H, m, axial H^{12} & H^{28}), 2.77 (4H, s, H^{32}), 2.52 (2H, m, equatorial H^{13} & H^{29}), 2.04–1.96 (2H, m, axial H^{13} & H^{29}), 1.76–1.69 (12H, m, H^{3}, H^{4}, H^{17} & H^{18}); δ_C (126 MHz, CD_3CN): 171.0, 169.7, 169.1, 168.4, 143.0, 142.6, 142.3, 141.9, 139.1, 130.9, 130.6, 129.8, 126.6, 124.5, 123.3, 111.8, 111.7, 111.0, 110.6, 70.7, 70.7, 49.8, 49.6, 42.3, 42.3, 37.9, 28.3, 28.3, 27.7, 27.6, 27.3, 27.2, 26.4; HRMS (ESI^+) *m/z*: [M^+] calcd. for $C_{35}H_{36}N_3O_5^+$ 578.2650; found 578.2631; Analytical HPLC (Method A): t_R = 12.3 min (in 560 nm channel).

Cy3B-NHBoc (hexafluorophosphate)

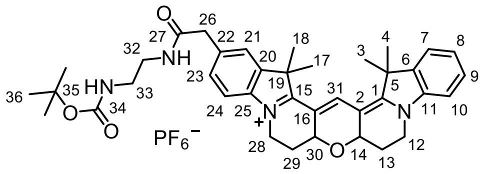

To a solution of **Cy3B-NHS ester** (hexafluorophosphate, 270 mg, 373 µmol) in CH_2Cl_2 was added a solution of *N*-Boc-ethylenediamine (63.0 mg, 392 µmol, 1.05 equiv.). The reaction mixture was stirred at 20° C for 18 h and concentrated *in vacuo*. The reaction mixture was diluted with CH_2Cl_2 (50 mL) and the solution was washed with water (150 mL) and an aqueous solution of KPF_6 (4% w/v, 100 mL). The organic layer was dried ($MgSO_4$) and concentrated in vacuo. The crude product was purified by flash column chromatography (0–7% MeOH in CH_2Cl_2) to yield the product as a black solid (200 mg, 70%). NMR δ_H (400 MHz, CD_3CN): 8.10 (1H, s, H^{31}), 7.54–7.15 (7H, m, H^{7-10}, H^{21}, H^{23} & H^{24}), 6.66 (1H, s, br, carbamate NH), 5.42 (1H, s, br, amide NH), 4.60 (2H, m, H^{14} & H^{30}), 4.22 (2H, m, equatorial H^{12} & H^{28}), 3.85 (2H, m, axial H^{12} & H^{28}), 3.51 (2H, s, H^{26}), 3.24–3.06 (4H, m, H^{32} & H^{33}), 2.53 (2H, m, equatorial H^{13} & H^{29}), 2.04–1.96 (2H, m, axial H^{13} & H^{29}), 1.74–1.70 (12H, m, H^{3}, H^{4}, H^{17} & H^{18}), 1.40 (9H, s, H^{36}); δ_C (101 MHz, CD_3CN): 171.4, 168.9, 168.8, 142.6, 141.6, 141.4, 138.4, 134.5, 130.2, 129.3, 126.0, 124.0, 122.9, 111.2, 111.1, 110.3, 110.1, 79.0, 70.3, 70.2, 54.9, 49.3, 49.2, 43.0, 41.9, 41.8, 40.7, 40.1, 28.2, 27.9, 27.9, 27.3, 27.3, 26.9; HRMS (ESI^+) *m/z*: [M^+] calcd. for $C_{35}H_{36}N_3O_5^+$ 578.2655; found 578.2648; Analytical HPLC (Method A): t_R = 12.5 min (in 560 nm channel).

DTE-COO^{t}Bu

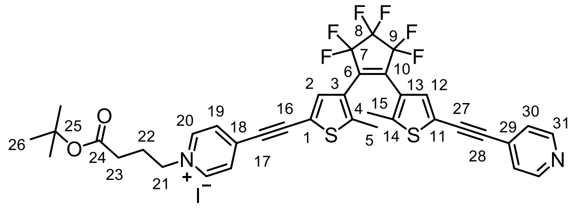

To a solution of *t*-butyl-4-bromobutanoate (450 mg, 2.02 mmol, 1 equiv.) and potassium iodide (502 mg, 1.5 equiv.) in MeCN (50 mL) was added a solution of **DTE-dipy** (1.73 g, 3.03 mmol, 1.5 equiv.) in CH_2Cl_2 (10 mL). The reaction mixture was stirred at 65 °C for 60 h before being cooled to room temperature and concentrated in vacuo. The resulting solid was dissolved in MeCN and precipitated with CH_2Cl_2 twice to remove inorganic salts. The crude product was purified by flash column chromatography (CH_2Cl_2 1:1 EtOAc, then 1–10% MeOH in CH_2Cl_2) to yield the product as an off-white solid (310 mg, 18%). NMR δ_H (400 MHz, CD_3CN): 8.64 (2H, d, J_{HH} 6.3, H^{20}), 8.60 (2H, d, J_{HH} 4.7, H^{31}), 7.98 (2H, d, J_{HH} 6.3, H^{19}), 7.61 (1H, s, H^2), 7.42–7.38 (3H, m, H^{12} & H^{30}), 4.52 (2H, t, J_{HH} 7.5, H^{21}), 2.34 (2H, t, J_{HH} 7.2, H^{23}), 2.19 (2H, appr. p, H^{22}), 2.06 (3H, s, H^5), 2.02 (3H, s, H^{14}), 1.42 (9H, s, H^{26}); HRMS (ESI$^+$) *m/z*: [M$^+$] calcd. for $C_{37}H_{31}F_6N_2O_2S_2^+$ 713.1731; found 713.1738; Analytical HPLC (Method A): t_R = 13.5 min (in 360 nm channel).

DTE-Cy3B

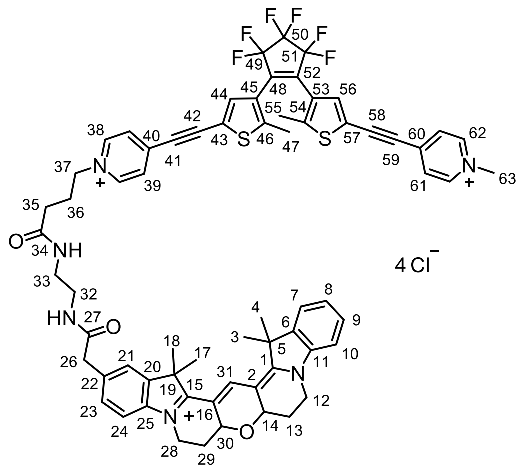

Deprotection of **DTE-COO^{t}Bu**: **DTE-COO^{t}Bu** (iodide, 20 mg, 24 µmol) was dissolved in a 9:1 mixture CH_2Cl_2/TFA (1 mL) and the resulting solution was stirred at 20 °C for 18 h. The solvent was evaporated under reduced pressure and the residue was co-distilled with dichloromethane (5 × 5 mL). The pale green solid and was used immediately without further purification. Analytical HPLC (Method A): t_R = 11.8 min (in 360 nm channel).

Deprotection of **Cy3B-NHBoc**: **Cy3B-NHBoc** (hexafluorophosphate, 19 mg, 24 µmol, 1 equiv.) was dissolved in a 9:1 mixture CH_2Cl_2/TFA (1 mL) and the resulting solution was stirred at 20 °C for 1.5 h. The solvent was evaporated under reduced pressure and the residue was co-distilled with dichloromethane (5 × 5 mL). Analytical HPLC (Method A): t_R = 9.5 min (in 560 nm channel).

The deprotected **DTE-COO^{t}Bu**, HBTU (10 mg, 26 μmol, 1.1 equiv.) and DIPEA (33 μL, 190 μmol, 8 equiv.) were mixed in DMF (0.5 mL). The reaction mixture for activating the carboxylic acid group was stirred at 20° C for 30 min and then transferred to the solution of deprotected **Cy3B-NHBoc** in DMF. The amide coupling reaction mixture was stirred at 20 °C for 1.5 h. Upon completion of the amide coupling reaction verified by analytical HPLC, excess iodomethane (10 mL) was added to the reaction mixture to methylate the DTE pyridine and quench the remaining DIPEA. The methylation reaction mixture was stirred at 20 °C for 40 h and then concentrated in vacuo. The resulting compound was purified by preparative HPLC (method B). The purified compound was dissolved in MeCN (1 mL) and added to an aqueous solution of KPF_6 (4% w/v, 30 mL) and stirred for 30 min. The precipitate was collected, dissolved in MeCN (1 mL) and then added to a solution of tetrabutylammonium chloride in acetone (10% w/v, 20 mL). The ion exchange reaction was stirred at 20 °C for 18 h. The precipitate was collected to yield the final product as a black solid (16 mg, 51%). NMR δ_H (600 MHz, CD_3CN): 8.74 (2H, d, J_{HH} 6.9, H^{38}), 8.58 (2H, d, J_{HH} 6.9, H^{62}), 8.09 (1H, s, H^{31}), 7.99–7.90 (4H, m, H^{39} & H^{61}), 7.81–7.71 (2H, m, NH), 7.64–7.58 (2H, 2 × s, H^{44} & H^{56}), 7.54–7.10 (7H, m, H^{7-10}, H^{21}, H^{23}, H^{24} & H^{25}), 4.59 (2H, m, H^{14} & H^{30}), 4.53 (2H, t, J_{HH} 7.1, H^{37}), 7.27–7.16 (5H, m incl. s, H^{63} & equatorial H^{12} & H^{28}), 3.85 (2H, m, axial H^{12} & H^{28}), 3.54 (2H, s, H^{26}), 3.21 (4H, m, H^{32} & H^{33}), 2.52 (2H, m, equatorial H^{13} & H^{29}), 2.27 (2H, t, J_{HH} 7.1, H^{35}), 2.22 (2H, appr. p, J_{HH} 7.1, H^{36}), 2.05–2.03 (6H, 2 × s, H^{47} & H^{55}), 2.00–1.96 (2H, m, axial H^{13} & H^{29}), 1.73–1.68 (12H, 4 × s, H^3, H^4, H^{17} & H^{18}); δ_C (151 MHz, CD^3CN) 172.3, 171.9, 169.4, 169.2, 160.7, 160.4, 150.1, 146.2, 145.7, 143.1, 142.0, 141.8, 141.7, 140.5, 140.3, 138.8, 136.8, 136.7, 135.4, 130.6, 129.9, 129.7, 126.4, 126.1, 124.4, 123.3, 119.8, 118.7, 116.8, 111.6, 111.4, 110.7, 110.5, 96.4, 96.3, 90.8, 90.7, 70.7, 70.7, 61.9, 49.7, 49.6, 49.1, 43.4, 42.3, 42.2, 40.0, 39.9, 34.5, 32.5, 28.4, 27.7, 27.3, 26.5, 25.8, 15.1; HRMS (ESI$^+$) *m/z*: [M^{3+}] calcd. for $C_{67}H_{63}F_6N_6O_3S_2^{3+}$ 392.4764, found 392.4757; Analytical HPLC (Method A): t_R = 12.8 min (in both 360 nm and 560 nm channels); UV-Vis (water): $\lambda_{max,\ DTE}$ (ε) = 377 nm (69500 mol^{-1} dm^3 cm^{-1}); $\lambda_{max,\ cyanine}$ (ε) = 555 nm (158,000 mol^{-1} dm^3 cm^{-1}); fluorescence (water): λ_{em} = 564 nm; $\Phi_{fl.}$ = 61%.

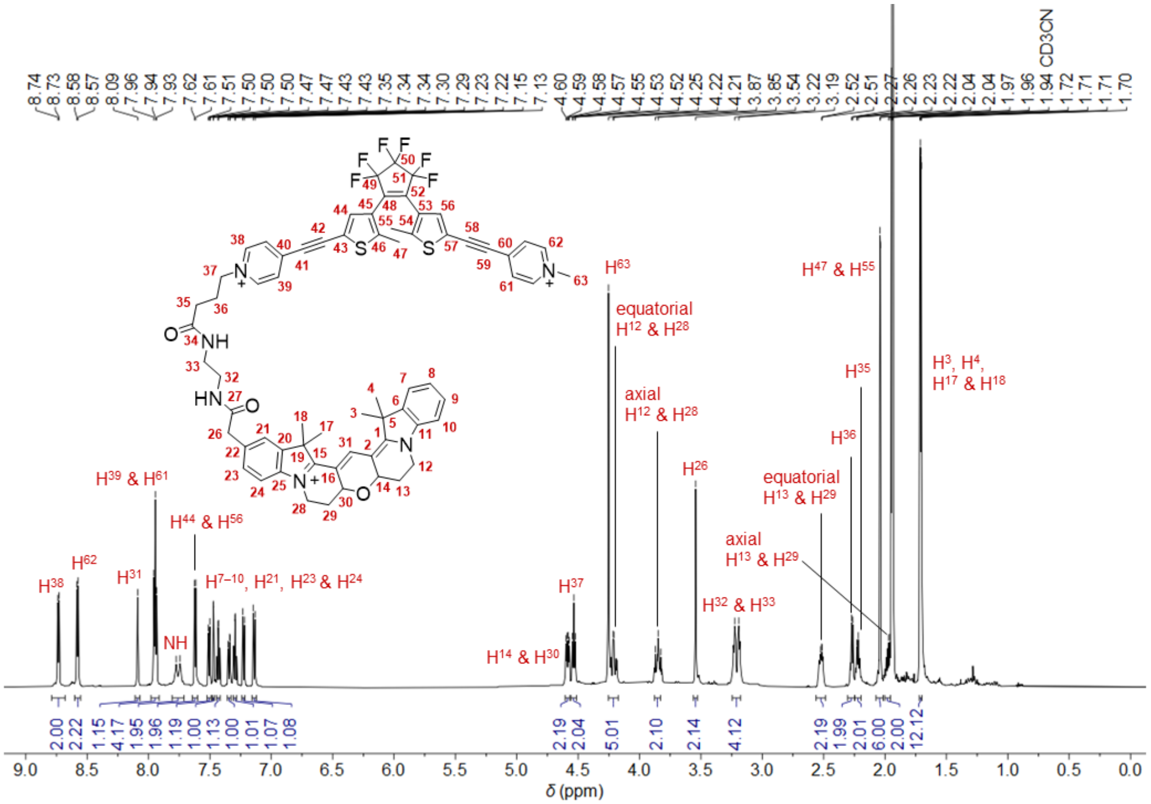

Figure S48. ^{1}H NMR spectrum (600 MHz, CD_3CN) of **DTE-Cy3B**.

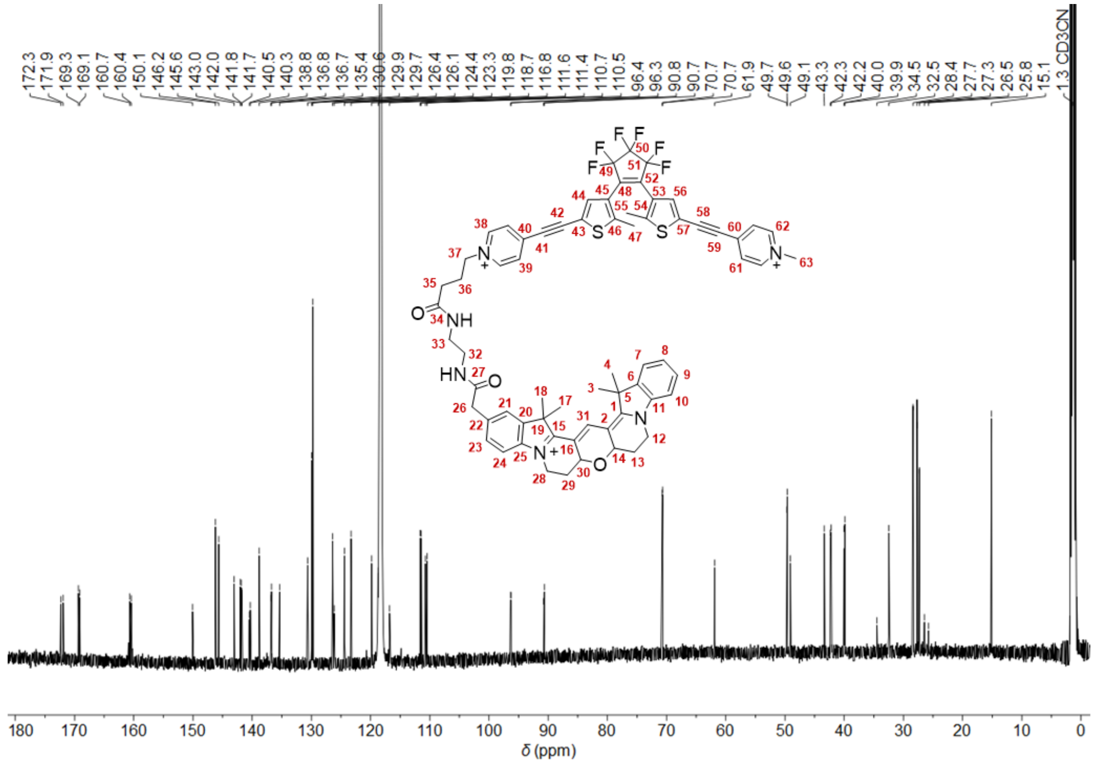

Figure S49. ^{13}C NMR spectrum (151 MHz, CD_3CN) of **DTE-Cy3B**.

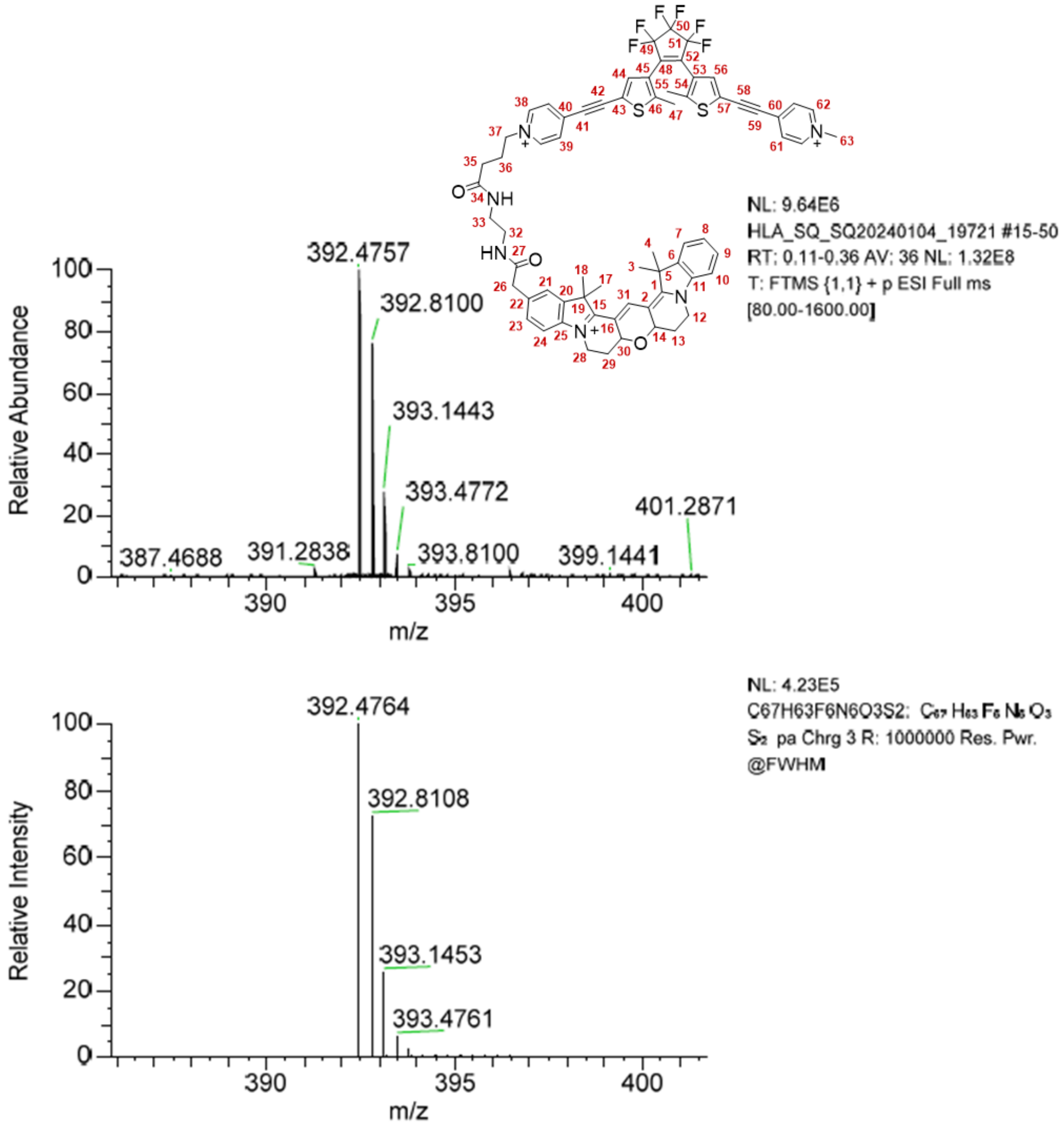

Figure S50. Experimental HRMS (ESI+) pattern of **DTE-Cy3B** and the theoretical pattern.

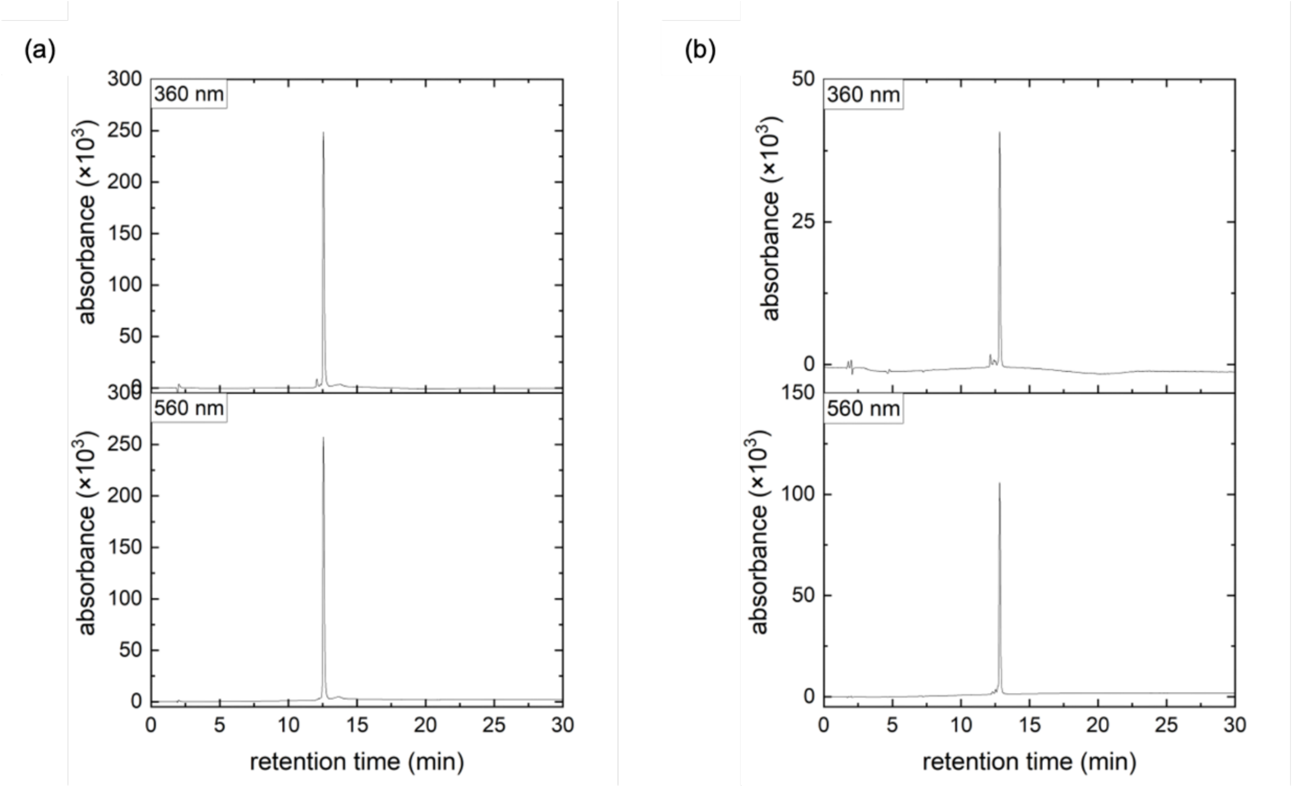

Figure S51. Analytical HPLC trace (method A) of (a) **DTE-Cy3** and (b) **DTE-Cy3B** monitored at 360 nm (DTE absorption range) and 560 nm (Cy3 absorption range).

References

1. M. S. Maier, K. Hull, M. Reynders, B. S. Matsuura, P. Leippe, T. Ko, L. Schaffer and D. Trauner, *J. Am. Chem. Soc.*, 2019, **141**, 17295–17304.
2. D. Lachmann, C. Studte, B. Mannel, H. Hubner, P. Gmeiner and B. Konig, *Chem. Eur. J.*, 2017, **23**, 13423–13434.
3. N. A. Simeth, L. M. Altmann, N. Wossner, E. Bauer, M. Jung and B. Konig, *J. Org. Chem.*, 2018, **83**, 7919–7927.
4. D. Lachmann, R. Lahmy and B. König, *Eur. J. Org. Chem.*, 2019, **2019**, 5018–5024.
5. S. Qiu, A. T. Frawley, K. G. Leslie and H. L. Anderson, *Chem. Sci.*, 2023, **14**, 9123–9135.
6. L. M. Hall, M. Gerowska and T. Brown, *Nucleic Acids Res.*, 2012, **40**, e108.
7. M. M. Ahlström, M. Ridderström, I. Zamora and K. Luthman, *J. Med. Chem.*, 2007, **50**, 4444–4452.
8. A.S. Waggoner and R. B. Mujumdar,*US Pat.,* US20030224391A1, 2003.
9. R. F. Kubin and A. N. Fletcher, *J. Lumin.*, 1982, **27**, 455–462.
10. D. N. Rao, X. Ji and S. C. Miller, *Chem. Sci.*, 2022, **13**, 6081–6088.
11. M. Gerowska, L. Hall, J. Richardson, M. Shelbourne and T. Brown, *Tetrahedron*, 2012, **68**, 857–864.
12. J. Song, S. Min, D. Lee, K. Park, M. Hwang, B. Shin and J. Je, *US Pat.*, US10473666B2, 2019.
